# First-in-Class Inhibitors of the Ribosomal Oxygenase MINA53

**DOI:** 10.1021/acs.jmedchem.1c00605

**Published:** 2021-11-29

**Authors:** Radosław P. Nowak, Anthony Tumber, Eline Hendrix, Mohammad Salik Zeya Ansari, Manuela Sabatino, Lorenzo Antonini, Regina Andrijes, Eidarus Salah, Nicola Mautone, Francesca Romana Pellegrini, Klemensas Simelis, Akane Kawamura, Catrine Johansson, Daniela Passeri, Roberto Pellicciari, Alessia Ciogli, Donatella Del Bufalo, Rino Ragno, Mathew L. Coleman, Daniela Trisciuoglio, Antonello Mai, Udo Oppermann, Christopher J. Schofield, Dante Rotili

**Affiliations:** † Botnar Research Centre, Nuffield Orthopaedic Centre, University of Oxford, Headington OX3 7LD, U.K.; ‡ Chemistry Research Laboratory, Department of Chemistry and the Ineos Oxford Institute for Antimicrobial Research, 12, Mansfield Road, University of Oxford, Oxford OX1 3TA, U.K.; § Institute of Cancer and Genomic Sciences, University of Birmingham, Edgbaston, Birmingham B15 2TT, U.K.; ∥ Institute of Molecular Biology and Pathology (IMBP), National Research Council (CNR) c/o Department of Biology and Biotechnology “Charles Darwin” Sapienza University of Rome, Via degli Apuli 4, Rome 00185, Italy; ⊥ Rome Center for Molecular Design, Department of Chemistry and Technology of Drugs, ″Sapienza″ University of Rome, Piazzale Aldo Moro 5, Rome 00185, Italy; # Department of Chemistry and Technology of Drugs, ″Sapienza″ University of Rome, Piazzale Aldo Moro 5, Rome 00185, Italy; ∇ Chemistry - School of Natural and Environmental Sciences, Newcastle University, Newcastle upon Tyne NE1 7RU, U.K.; ○ TES Pharma S.r.l. Via P. Togliatti 20, Corciano, Perugia 06073, Italy; ◆ Preclinical Models and New Therapeutic Agents Unit, IRCCS-Regina Elena National Cancer Institute, Via Elio Chianesi 53, Rome 00144, Italy

## Abstract

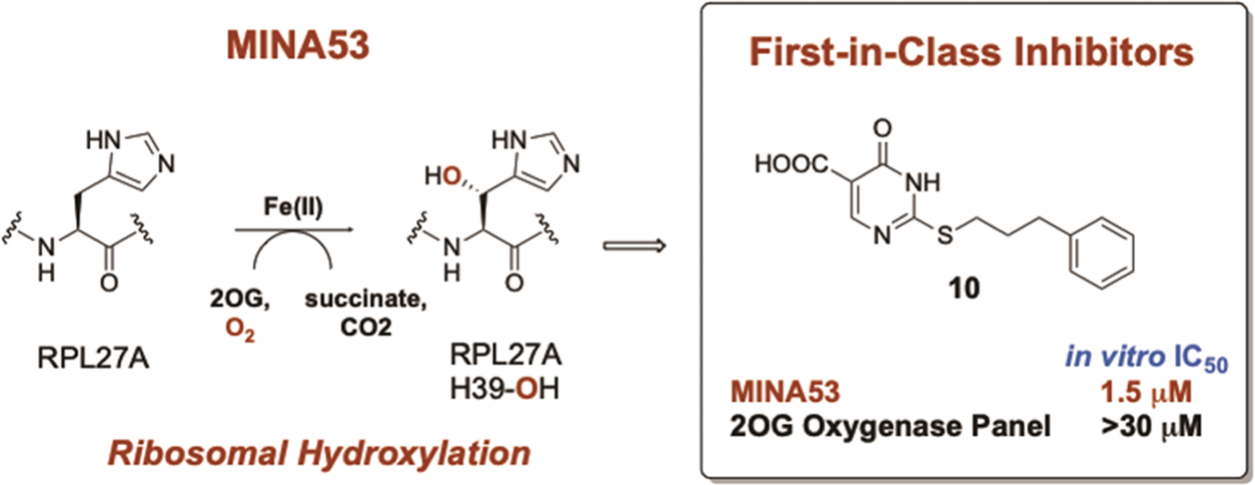

MINA53 is a JmjC domain 2-oxoglutarate-dependent oxygenase that catalyzes ribosomal hydroxylation and is a target of the oncogenic transcription factor *c*-MYC. Despite its anticancer target potential, no small-molecule MINA53 inhibitors are reported. Using ribosomal substrate fragments, we developed mass spectrometry assays for MINA53 and the related oxygenase NO66. These assays enabled the identification of 2-(aryl)alkylthio-3,4-dihydro-4-oxoypyrimidine-5-carboxylic acids as potent MINA53 inhibitors, with selectivity over NO66 and other JmjC oxygenases. Crystallographic studies with the JmjC demethylase KDM5B revealed active site binding but without direct metal chelation; however, molecular modeling investigations indicated that the inhibitors bind to MINA53 by directly interacting with the iron cofactor. The MINA53 inhibitors manifest evidence for target engagement and selectivity for MINA53 over KDM4–6. The MINA53 inhibitors show antiproliferative activity with solid cancer lines and sensitize cancer cells to conventional chemotherapy, suggesting that further work investigating their potential in combination therapies is warranted.

## Introduction

MYC-induced nuclear antigen (MINA53), also known as mineral dust-induced gene (Mdig) and ribosomal oxygenase 2 (RIOX2), is a JmjC (Jumonji-C) domain-containing 2-oxoglutarate (2OG)-dependent oxygenase localizing to the nucleolus,^1^ which is transcriptionally stimulated by the oncoprotein *c*-MYC.^2^ MINA53 upregulation is linked to solid and hematological tumors, including colon, lung, esophageal, gastric, pancreatic, renal, and hepatocellular carcinomas, breast cancer, leukemias, lymphomas/multiple myelomas, neuroblastomas, and glioblastomas. Elevated *MINA53* expression is reported as a poor prognostic indicator, and there is evidence that MINA53 downregulation impairs the proliferation and survival of cancer cells.^3−11^*MINA53* expression is induced by silica particles, suggesting a role for MINA53 in allergen-induced inflammation,^12^ and, importantly, in the differentiation of proinflammatory TH17 cells.^13^ MINA53 is proposed as an important regulator in inflammation and oncology; however, the underlying molecular mechanisms by which MINA53 is linked to disease are unclear.^11^

**Figure 1:**
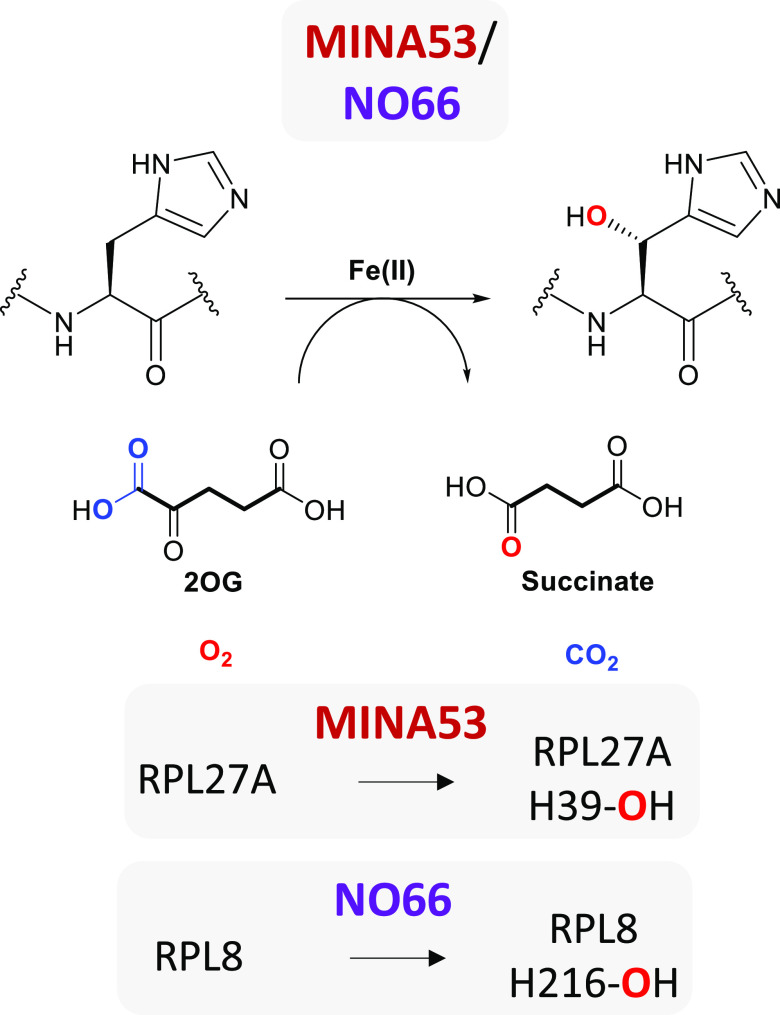
Ribosomal protein hydroxylation reactions catalyzed by the 2-oxoglutarate (2OG)-dependent histidine-residue C-3 hydroxylases MINA53 and NO66.

In early cellular studies, MINA53 was reported to cause demethylation of H3K9me3,^14^ but this catalytic activity has not been validated with isolated MINA53 under conditions where other JmjC lysine demethylases (KDMs) are active,^15^ and the MINA53 structure is not supportive of its proposed role as a canonical KDM.^16^ More recently, MINA53 has been shown to catalyze hydroxylation of a histidine residue in the ribosomal protein RPL27A in studies with both isolated components and in cells, suggesting its function in ribosomal regulation (Figure 1).^15−17^ NO66 (nucleolar protein 66), which has significant sequence homology with MINA53, was also initially reported as a histone KDM specific for H3K4Me3 and H3K36Me3,^18^ but, like MINA53, this activity is not validated. NO66 (or RIOX1 (ribosomal oxygenase 1)) catalyzes histidinyl hydroxylation of the ribosomal protein RPL8 (Figure 1).^15−17,19,20^

In addition to roles in skeletal growth and bone formation,^17,18,20,21^ NO66 is linked to cancer, though less well than MINA53; its gene is overexpressed in some tumor cell lines, such as colorectal and nonsmall-cell lung carcinomas, where NO66 downregulation impairs proliferation, survival, and migration.^17,20,22^

Despite its strong cancer links, to date, there are no reported MINA53 inhibitors; such compounds would enable a better understanding of the biological roles of MINA53 and its therapeutic potential as a target, especially for oncology. Both isolated MINA53 and NO66 lack KDM activity,^15^ and no specific antibodies are available for their hydroxylated ribosomal products. To identify potent and selective (including over NO66) MINA53 inhibitors, we thus developed a medium-throughput mass spectrometry-based assay,^23^ employing hydroxylation of synthetic ribosomal fragments specific for MINA53 and NO66. Following assay optimization and validation with known broad-spectrum 2OG oxygenase inhibitors, we screened for new types of 2OG oxygenase inhibitors acting on MINA53. The results led to the identification of 2-substituted-3,4-dihydro-4-oxopyrimidine-5-carboxylic acids as a novel class of 2OG oxygenase inhibitors, which manifest selectivity for MINA53.

## Results and Discussion

### Assay Development

**Figure 2:**
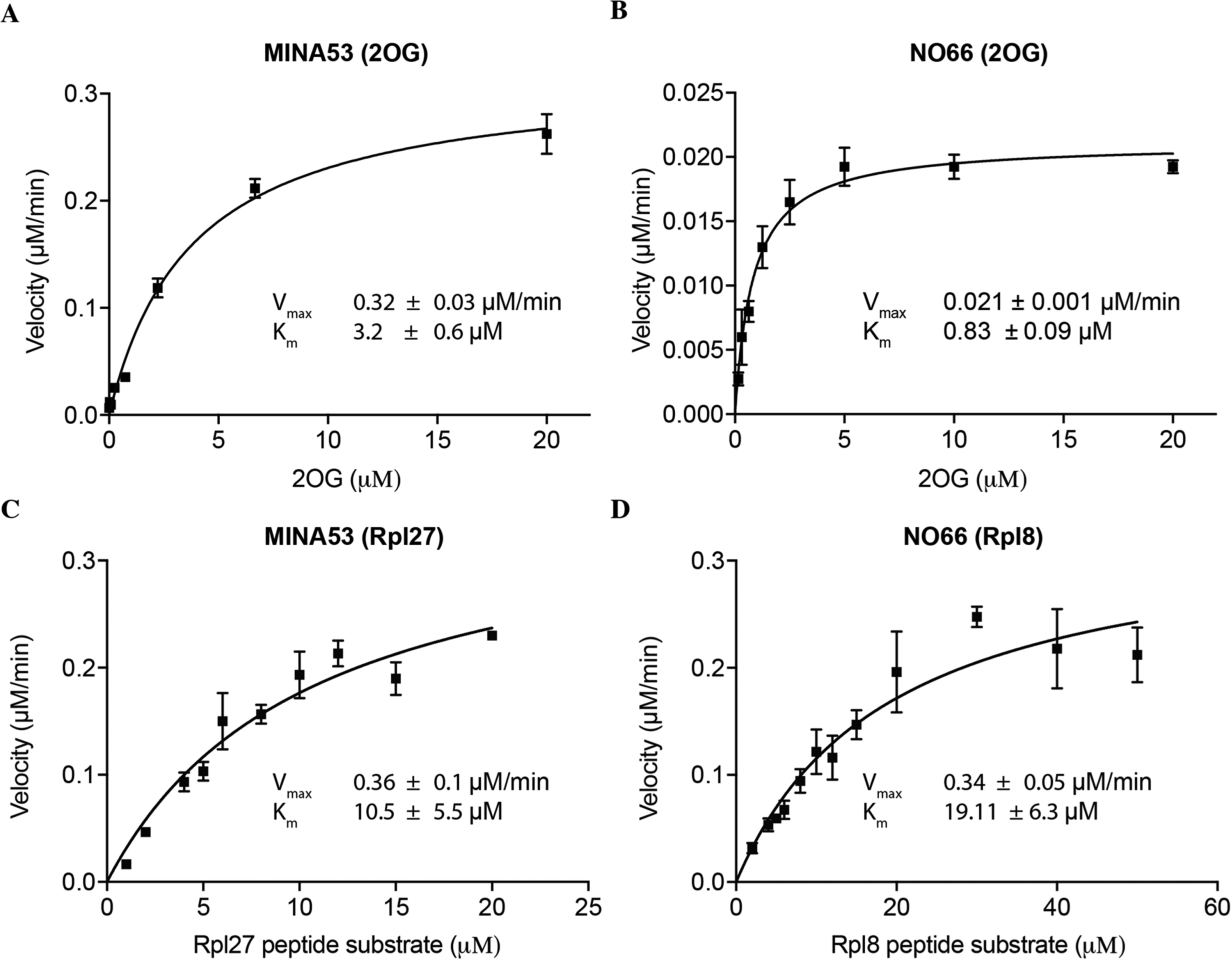
Michaelis–Menten characterization of MINA53 and NO66 for the 2OG cosubstrate and ribosomal peptide substrates. (A) Determination of the *K*_M_ for 2OG with MINA53 (Met26-Val464) and (B) NO66 (Ser183-Asn641). Values are means ± 95% confidence, *n* = 4. (C) Determination of *K*_M_ for the peptide substrate with MINA53 (Met1-Val464) and (D) NO66 (Gln116-Asn641). Values are means ± 95% confidence, *n* = 3. See Table S4 for assay details.

A solid-phase extraction-linked to the MS (RapidFire) assay based on the simultaneous detection of the disappearance of substrates and formation of the hydroxylated products Rpl27a-His39OH and Rpl8-His216OH for MINA53 and NO66, respectively, was developed (Figures 1 and S1). For MINA53, the RPL27A fragment used was G_31_RGNAGGL**H**HHRINFDKYHP_49_, and for NO66, it was RPL8 N_205_PVEHPFGGGN**H**QHIGKPST_224_. Kinetic parameters for the peptide substrates, 2OG, and Fe^2+^ and assay sensitivity to dimethyl sulfoxide (DMSO) were evaluated for both enzymes (Figures 2, S2, and S3); buffer composition, pH, and temperature were optimized (Figures S4–S6 and Table S4).

**Figure 3:**
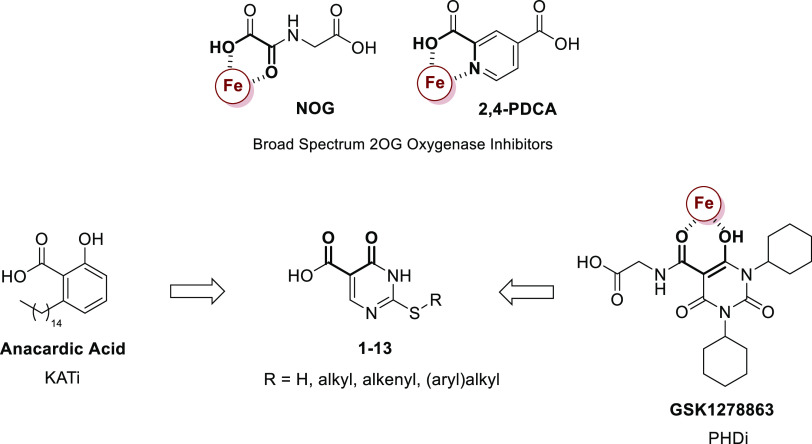
Initial approach to the MINA53 inhibitors employed in the study. *N*-Oxalylglycine (NOG) and pyridine-2,4-dicarboxylate (2,4-PDCA) are broad-spectrum 2OG oxygenase inhibitors.^24^ GSK1278863 is a prolyl hydroxylase domain (PHD) inhibitor. The observed active Fe(II) (in red) chelating groups are highlighted in bold for NOG, 2,4-PDCA, and GSK1278863 as that proposed for the 2-(aryl)alkylthio-3,4-dihydro-4-oxoypyrimidine-5-carboxylic acids. Anacardic acid is an inhibitor of histone lysine acetyltransferases (KATs).^27^

The assay was validated using known broad-spectrum 2OG oxygenase inhibitors, i.e., pyridine-2,4-dicarboxylate (2,4-PDCA, Figure 3),^24^*N*-oxalylglycine (NOG, Figure 3),^24^ and IOX-1,^25^ and the JMJD3/KDM5 inhibitor GSK-J1.^26^ GSK-J1 did not show significant inhibition of MINA53 or NO66 at 100 μM, and IOX-1 was also a poor inhibitor (IC_50_ 38.5 μM for NO66 and 101.8 μM for MINA53). By contrast, both NOG and 2,4-PDCA, which are reported as relatively broad-spectrum 2OG oxygenase inhibitors,^24^ were rather potent inhibitors of both NO66 and MINA53 (NOG IC_50_ of 3.5 μM for NO66 and 1.8 μM for MINA53, and 2,4-PDCA IC_50_ of 0.11 μM for NO66 and 1.3 μM for MINA53) (Figure S7). The IC_50_ values for NOG and 2,4-PDCA are amongst the lowest concentrations reported for the inhibition of human 2OG oxygenases by these two compounds. Thus, given that the prodrug ester forms of these compounds have been widely used in cell biology, it is possible that some of the resultant cellular observations reflect inhibition of MINA53 and/or NO66.^24^

During screening work, we tested the 2-substituted-3,4-dihydro-4-oxopyrimidine-5-carboxylic acids (**1–13**) against MINA53/NO66 (Figure 3). These molecules were initially prepared as analogues of the histone lysine acetyltransferase (KAT) inhibitor anacardic acid (AA),^27^ but were inactive versus KATs (Table S1). We envisaged that the 4-keto and the 5-carboxy groups of **1–13** might act analogously to some 4-hydroxy-5-carbonyl-substituted pyrimidine 2OG oxygenase inhibitors, e.g., the prolyl hydroxylase inhibitor GSK1278863,^28^ by chelating the active site ferrous iron (Figure 3).

Although subsequent structural studies question this proposal, our biochemical, biophysical, and cellular results reveal that 2-substituted-3,4-dihydro-4-oxoypyrimidine-5-carboxylic acids have considerable potential as potent and selective inhibitors of ribosomal oxygenases.

### Synthesis of 2-Substituted-3,4-dihydro-4-oxopyrimidine-5-carboxylic Acids

**Scheme 1:**
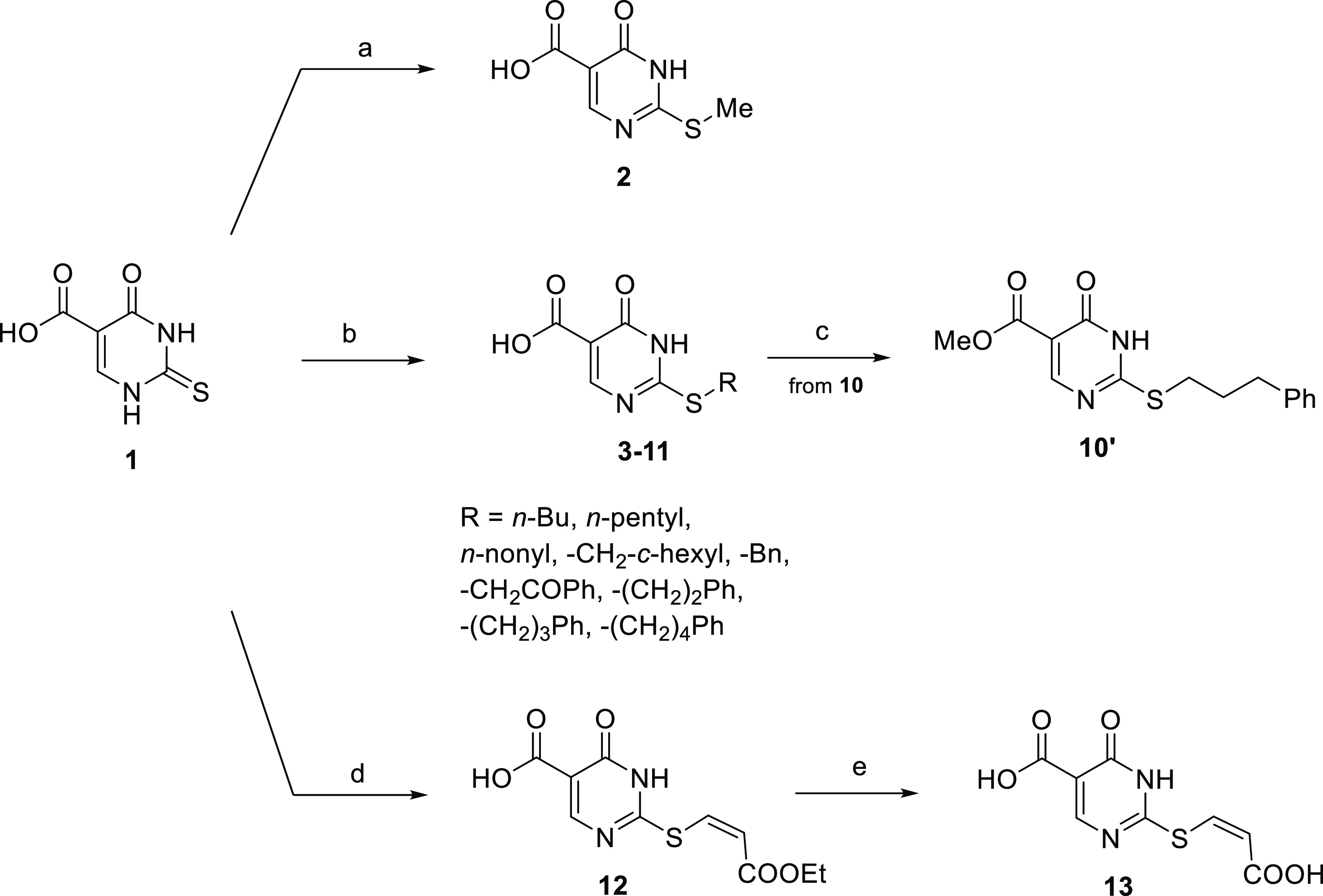
Synthesis of Compounds **2**–**13** Reagents and conditions: (a) methyl iodide, dry DMF, rt; (b) (cyclo)alkyl-/arylalkyl bromide, anhydrous potassium carbonate, dry DMF, rt; (c) (i) cesium carbonate, MeOH, rt, (ii) methyl iodide, dry DMF, 0 °C → rt; (d) ethyl propiolate, tetrabutylammonium fluoride, dry THF, rt; and (e) potassium hydroxide 2 N, ethanol, rt.

The routes used for the synthesis of 2-substituted-3,4-dihydro-4-oxopyrimidine-5-carboxylic acid derivatives **2–13** are shown in Scheme 1. **2** was obtained by alkylation of commercially available **1** with methyl iodide in dried *N*,*N*-dimethylformamide (DMF). **3**–**11** were synthesized by alkylation of **1** with the requisite (cyclo)alkyl-/arylalkyl bromide in the presence of anhydrous potassium carbonate in dried DMF. **10′** was prepared by treatment of **10** with cesium carbonate in aqueous methanol, followed by the alkylation of the resulting cesium salt with methyl iodide in dried DMF. **12** was prepared by reaction of **1** with ethyl propiolate and tetrabutylammonium fluoride in dried tetrahydrofuran (THF). Diacid **13** was prepared by the hydrolysis of **12** with 2 N potassium hydroxide in ethanol. Chemical–physical data and elemental analyses for **2–13** are reported in Tables S2 and S3 (Supporting Information), respectively; high-performance liquid chromatography (HPLC) traces for compounds **7**–**10** are reported in Figures S20–S23 (Supporting Information).

### Identification of Potent MINA53 Inhibitors

The selectivity profiles and potencies of **1–13** are presented in Table 1 (all compounds tested were inactive in AlphaScreen assay controls, Figure S8). It is important to note that the selectivity data with isolated enzymes do not necessarily reflect the in-cell situation, in part because some of the enzymes and all of the substrate peptides used are truncated constructs. Nonetheless, the results reveal the potential of 2-substituted-3,4-dihydro-4-oxopyrimidine-5-carboxylic acids for potent and, at least partially, selective MINA53 inhibition. The progenitor of the series, **1**, was a weak inhibitor of both MINA53 (IC_50_ ∼ 200 μM) and NO66; of the other tested 2OG oxygenases, it was only slightly active versus KDM3B and KDM5B (Table 1). Interestingly, simple *S*-methylation of the C-2 thioxo group of the pyrimidine increased activity toward MINA53 by about 2 orders of magnitude, conferring on **2** a MINA53 IC_50_ in the single-digit micromolar range. With the exceptions of KDM3B and KDM5B, a substantial increase of the inhibitory potency with respect to **1** was also observed with the other tested enzymes, in particular FIH (factor inhibiting hypoxia-inducible factor, HIF, which like MINA53 is a protein hydroxylase that can act on histidyl and other residues)^29,30^ and the JmjC KDM KDM4A. However, **2** still retained a ∼5-fold preference for the inhibition of MINA53 over both of these hydroxylases (albeit under different assay conditions).

**Table 1:**
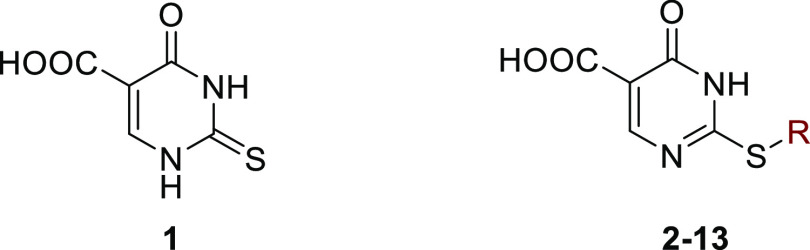

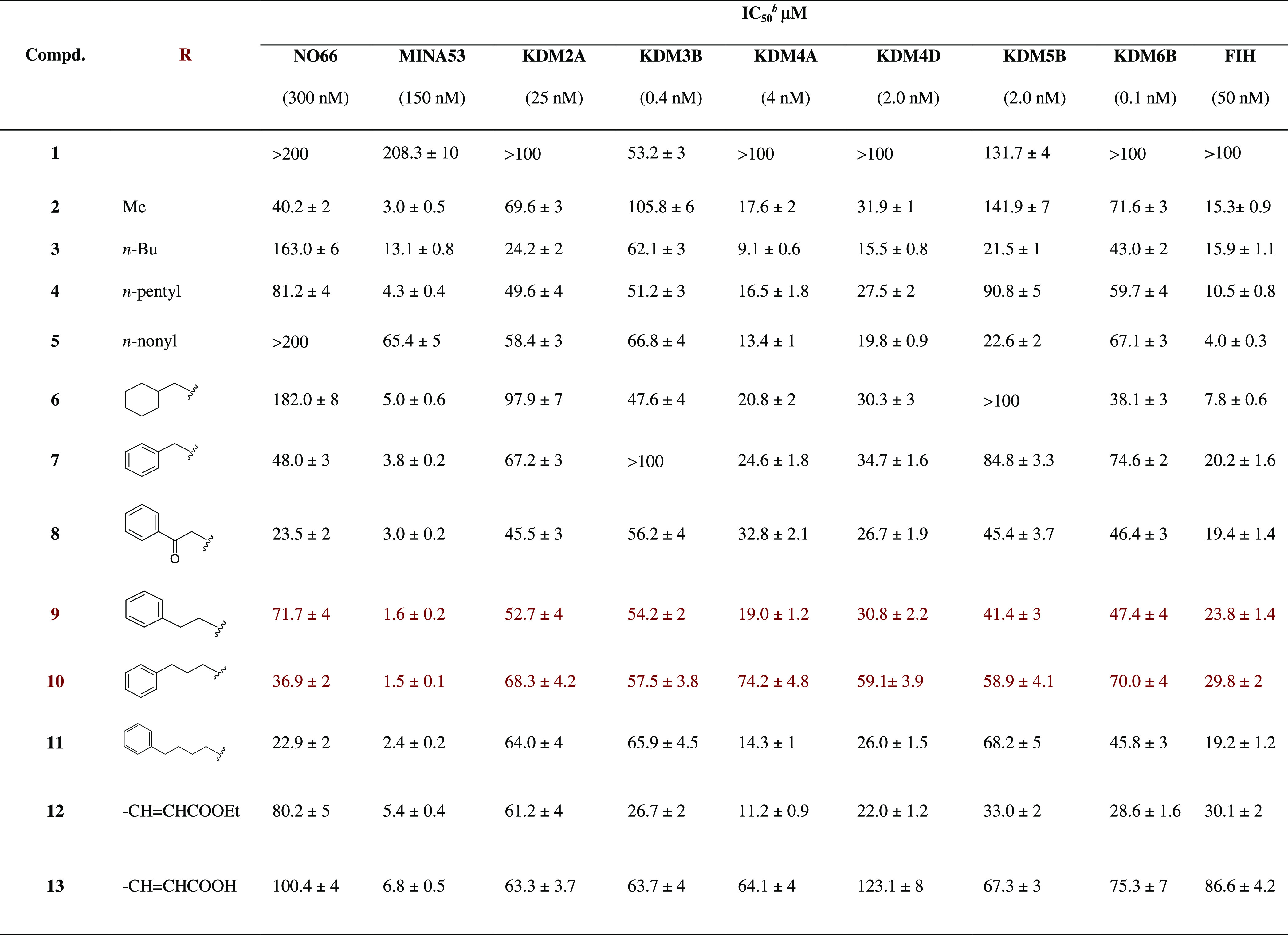
Inhibitory Activity of **1–13** against a Panel of Human 2-Oxoglutarate Oxygenases^a^

a Values are means ± standard deviation (SD) of at least three separate experiments.

b Inhibitory dose 50: dose required to inhibit the enzymatic activity by 50%; NO66, MINA53, and FIH assays are MS based, while the KDM assays were performed by the AlphaScreen method. Enzyme concentrations used in the assays are specified in brackets in the table header.

Homologation of the C-2 pyrimidine alkylthio chain by the addition of 3 or 4 methylene groups caused a decrease in potency for both MINA53 and NO66 and loss of MINA53 selective inhibition (compare **3** and **4** with **2**, Table 1). The decrease in the activity versus both MINA53 and NO66 is particularly evident with a 9 carbon unit side chain (compare **5** with **2**). While there were no substantial changes in the profile of activity toward KDMs, **5** showed a single-digit micromolar inhibition of FIH (IC_50_ of 4 μM). This observation is of interest given the lack of selective FIH inhibitors (the only available such compound is a prodrug)^31,32^ and the key role FIH has in the hypoxic response.^29^ A single-digit micromolar inhibitory potency and at least a 5-fold preferential inhibition of MINA53 over all other tested 2OG oxygenases were restored by the introduction of a benzylthio substituent at C-2 (**7**). While the saturation of the benzene ring decreased both MINA53 inhibitory activity and selectivity (compare **7** with **6**, Table 1), homologation of the C-2 side chain of **7** up to an optimal length of 2–3-methylene units (**9** and **10**, Table 1) led to the most effective observed MINA53 inhibition; at least in the case of **10**, this correlates with increased selectivity over the other tested 2OG oxygenases, including the other histidyl hydroxylases NO66^15,16,19^ and FIH^29,30^ (20-fold in the worst case). The insertion of a ketone within the aryl-alkyl side chain at C-2 of **9** to give **8** did not substantially decrease the activity toward most of the tested 2OG oxygenases with a slight decrease for MINA53 and >2-fold increase in potency for NO66 (compare **8** with **9**). The introduction of an acryloyl side chain at the same position (**12** and **13**) was detrimental in terms of both MINA53 and NO66 inhibition compared with the arylalkyl derivatives (e.g., **9** and **10**).

### Crystallization and Structure Analysis

**Figure 4:**
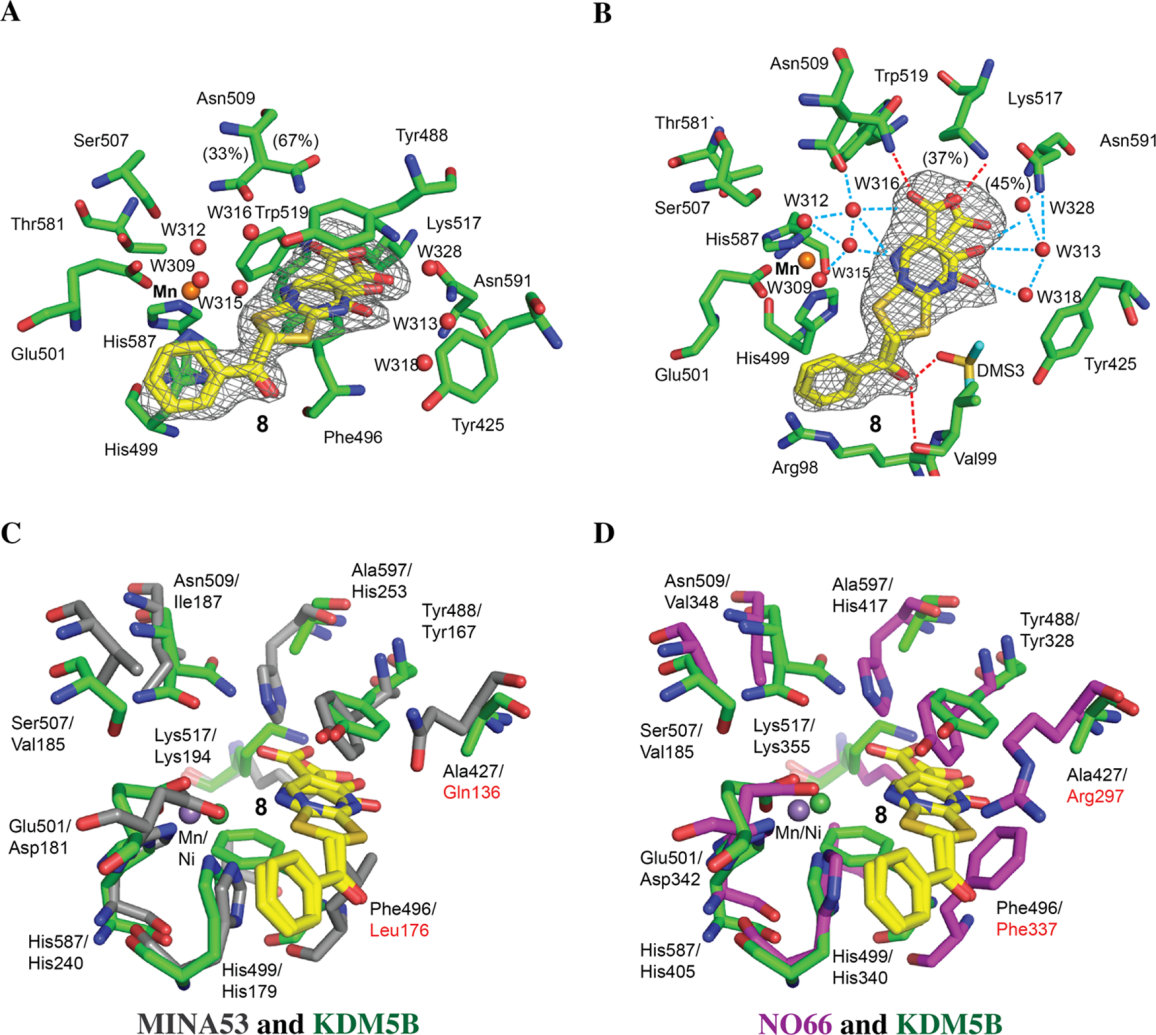
Views from a crystal structure of KDM5B in complex with **8** (PDB ID: 5FZI). (A) Residues within the 2OG binding pocket of KDM5B are in green sticks and **8** is in yellow sticks. Mn(II) that was used in crystallization is shown as an orange sphere; waters are red spheres. Hydrogen bond interactions are red dashed lines, and solvent-mediated interactions are dashed lines. Two alternative conformations with occupancies of 0.3 and 0.4 for **8** were refined to fit the density. The *F*_o_ – *F*_c_ OMIT density map is contoured at 3σ. The pyrimidine ring of **8** is positioned to make π–π interactions with Tyr488 and Phe496. Asn509 was refined in two conformations. (B) Alternative view showing the complex water network surrounding the ligand and water-mediated indirect metal interaction via W315. Tyr488 is not shown for clarity. Phe496 in B is not visible because it is obscured by the ligand. (C, D) Superimpositions of the KDM5B:**8** complex structure with structures of (C) MINA53 PDB ID: 2XDV (gray) and (D) NO66 PDB ID: 4DIQ (purple). Note that the structures imply that Phe337 of NO66 and Leu176 of MINA53 will form steric clashes with **8** if it were to bind to NO66 and MINA53 in an analogous manner to that observed for KDM5B and are highlighted in red.

Our attempts to obtain crystal structures of MINA53 in complex with the active inhibitors were unsuccessful. To investigate how the new pyrimidin-4(3*H*)-ones series binds to 2OG oxygenases, we therefore explored crystallography with other JmjC 2OG oxygenases, including KDM5B. Soaking of **2–13** into KDM5B crystals resulted in one cocrystal structure at 1.95 Å resolution with the moderate KDM5B inhibitor **8** (IC_50_ = 45 μM, Figures 4 and S9).

Analysis of the electron density for the KDM5B complex suggests that the pyrimidin-4(3*H*)-one ring of **8** adopts two conformations, and it was refined as such. Unexpectedly, **8** occupies the 2OG binding pocket of KDM5B but does not make a direct interaction with the active site metal ion; Mn(II) was used as an Fe(II) substitute to enable crystallization under aerobic conditions (note: we assume that inhibition proceeds without alteration of the Fe(II) redox state, though such a change cannot be entirely ruled out). The metal ion is coordinated by the highly conserved triad of residues amongst 2OG oxygenases (His499, His587, Glu501) and three water molecules. The carboxylic acid of **8** is positioned to interact with Lys517 and Asn509, as likely does 2OG during catalysis (Figure 4A). The binding of **8** is apparently further stabilized by a network of water molecules involving interactions with its pyrimidine nitrogen and the carboxylic acid (Figure 4B). The pyrimidine of **8** is positioned to π–π stack with the phenol ring of Tyr488, and the phenyl ring of the side chain of **8** is positioned between Arg98 and His499. The ketone oxygen in the side chain of **8**, which enhances potency versus NO66, but not KDM5B or MINA53 (Table 1), is positioned to form polar interactions with the backbone nitrogen of Val99 and a solvent-derived DMSO molecule (Figure 4B). Note that an analogously positioned solvent-derived DMSO molecule has been observed in multiple KDM5B crystal structures^33^ and may be partially responsible for the stabilization of the observed linker conformation of **8**.

Despite the conserved employment of the general JmjC-type 2OG binding mode, superimposition of the KDM5B and MINA53/NO66^16^ structures reveals considerable differences in their 2OG binding pockets (Figure 4C,D). In MINA53 (PDB ID: 2XDV), Leu176 adopts a position similar to Phe496 in KDM5B (Figure 4C); in NO66 (PDB ID: 4DIQ), Phe337 is similarly located, but its side chain points away from the metal-binding site (Figure 4D). KDM5B Phe496 apparently stabilizes binding of **8**, whereas the superimpositions imply that with NO66/MINA53 **8** will clash with the corresponding residue, i.e., conformational changes would be required for **8** to bind to NO66/MINA53 in the manner observed for KDM5B. Other differences in the 2OG binding sites likely contribute to the selectivity of **8** toward MINA53/NO66. In KDM5B, Ala427 occupies the same position as Gln136 and Arg297 in MINA53 and NO66, respectively. It is possible that these NO66/MINA53 residues form polar interactions with the 3,4-dihydro-4-oxopyrimidine-5-carboxylic acid of **8**. KDM5B Asn509, which helps form the 2OG binding pocket, occupies a position similar to MINA53 Ile187 and NO66 Val187. Even though polar interactions of **8** with MINA53 Ile187 and NO66 Val187 are not possible, it is possible that His253/417 of MINA53/NO66, directly or indirectly, mediate polar interactions with the inhibitors (Figure 4). It should also be noted that conformational changes during 2OG catalysis are likely poorly defined by the limited available crystallographic analyses and can be involved in inhibitor selectivity, as shown by work on other hydroxylases.^34^ Superimposition of structures of KDM5B complexed with **8** and of MINA53 in complex with the ribosomal peptide RPL27A led to the prediction of a steric clash between RPL27A Leu38 and the phenyl ring of **8**, suggesting mutually exclusive inhibitor and substrate binding (Figure S10). Thus, although it is reasonable to propose that **8** binds in a general manner similar to NO66/MINA53 as it does to KDM5B, there must be differences, and it cannot be ruled out that 3,4-dihydro-4-oxopyrimidines chelate the metal ion in the case of MINA53/NO66 (Figure S10).

### Molecular Modeling of Compounds **8** and **10**

There are three tautomers of **8** and **10** in which the readily exchangeable hydrogen can be either on the pyrimidine nitrogens (tautomers **8**_Tauto-1_, **8**_Tauto-2_, **10**_Tauto-1_, and **10**_Tauto-2_, Figure S11) or on the oxygen atom at the 4 position of the pyrimidine ring (**8**_Tauto-3_ and **10**_Tauto-3_, Figure S11). Quantum mechanical (QM) calculations (Figure S12) imply that the pyrimidn-4-(3*H*)-ones **8**_Tauto-1_ and **10**_Tauto-1_ are the most stable tautomers, with **8**_Tauto-2_, **8**_Tauto-3_, **10**_Tauto-2_, and **10**_Tauto-3_ being disfavored by 2.7–4.8 kcal/mol (see the Supporting Information Molecular Modeling section).

Molecular docking investigations of the tautomers of **8** and **10** with MINA53 and NO66 were performed using the program Plants^35^ and the Plp95 scoring function, as this combination was found to be the best performing in a docking assessment procedure^36^ using available crystal structures (see the Supporting Information Molecular Modeling section). In accord with the QM results, the docking results imply that tautomers **8**_Tauto-1_ and **10**_Tauto-1_ will preferentially bind with MINA53 (Table S8 and Figure S13). The proposed binding modes for **8** and **10** (Figure S13) were directly used as starting conformations for the subsequent molecular dynamics (MD) simulations.

MD simulations were performed on the three **8** tautomers with KDM5B (PDB entry code 5FZI) starting from the two different experimental poses (**8**_Pose-A_ and **8**_Pose-C_) resolved in the crystal structure (Figure 4). The analysis of six simulations (25 ns each) performed with all combinations of poses and tautomers (**8**_Pose A/Tauto-1_, **8**_Pose A/Tauto-2_, **8**_Pose A/Tauto-3_, **8**_Pose C/Tauto-1_, **8**_Pose C/Tauto-2_, **8**_Pose C/Tauto-3_) as well as MM/GBSA binding free energy calculations^37^ (see the Supporting Information Molecular Modeling section) indicates that **8**_Pose A/Tauto-1_ is the **8** preferred tautomeric state and binding pose combination (Figures S14 and S15, Table S9).

**Figure 5:**
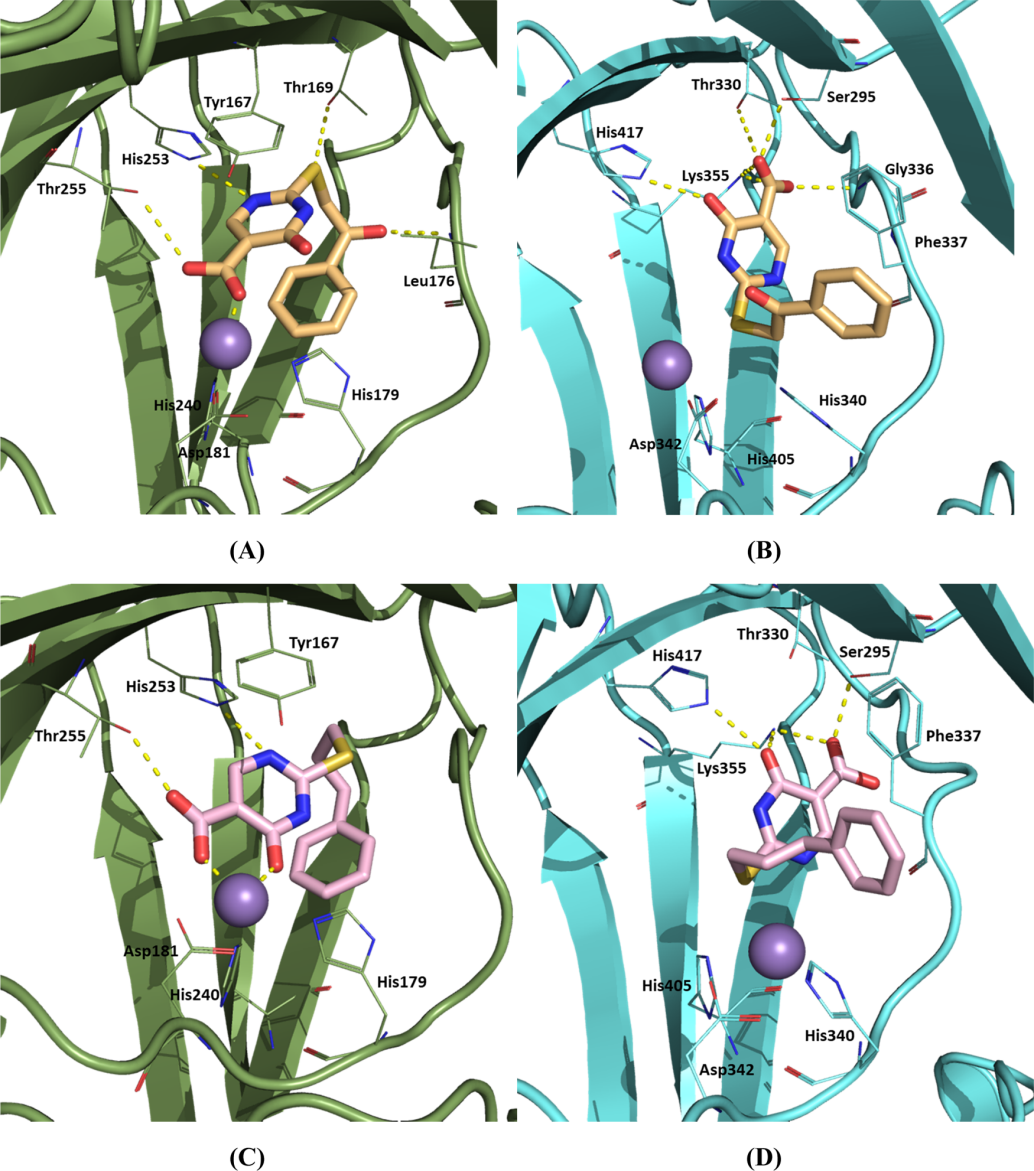
Representative MD frames depicting the proposed binding modes of compounds **8** and **10** with MINA53 and NO66. (A) **8**_Tauto-1_ BD binding mode with MINA53 (PDB ID: 4BXF); (B) **8**_Tauto-3_ LRC binding mode with NO66 (PDB ID: 4CCK); (C) **10**_Tauto-1_ BD binding mode with MINA53 (PDB ID: 4BXF); and (D) **10**_Tauto-1_ LRA binding mode with NO66 (PDB ID: 4CCK).

MD simulations were performed for MINA53 and NO66 as described for KDM5B. From the docking output, three binding poses were selected for MD investigations: the best docked (BD, the pose characterized by the lowest score) and poses presenting the lowest RMSD (LR) with respect to the crystallographically observed two conformations of compound **8** with KDM5B (PDB ID: 5FZI): A and C (LRA and LRC, respectively). This led to the following modeled complexes for MINA53 (PDB IDs: 4BXF and 2XDV) and NO66 (PDB ID: 4CCK), which were subjected to MD simulations: BD/4BXF, LRA/2XDV, LRC/2XDV, BD/4CCK, LRA/4CCK, and LRC/4CCK. The resulting MD trajectories were analyzed, and MM/GBSA binding free energies were calculated. The calculated Δ*G*s (Table S10) are consistent with the experimentally observed selectivity profile of **8** for 2-oxoglutarate oxygenases (MINA53 < NO66 < KDM5B). The large Δ*G* negative value for BD MINA53 (Table S10) substantially results from direct interaction with the active site metal ion. The lowest energy binding modes for **8** with MINA53 (BD, **8**_Tauto-1_) and NO66 (LRC, **8**_Tauto-3_) were visually inspected, taking a representative frame of the MD simulation. Interactions between **8**_Tauto-1_ and MINA53 (Figure 5A) include a hydrogen bond between His253 and the N1 nitrogen in the pyrimidine ring, a hydrogen bond between Thr255 and the carboxylate group at position 5 of the pyrimidine ring, a hydrogen bond between the Leu176 backbone and the inhibitor carbonyl oxygen, π–π stacking between Tyr167 and pyrimidine, and an interaction between the carboxylate group and the active site cation. Interactions between **8**_Tauto-3_ and NO66 (Figure 5B) include a hydrogen bond between His417 and the hydroxyl group at the pyrimidine 4 position, hydrogen bonds between Ser295, Thr330, Lys355, Gly336, and the carboxylate function, and π–π stacking between Phe337 and the phenyl ring of **8**_Tauto-3_.

Calculations to investigate the selective inhibition of MINA53 over NO66 by **10** followed the same procedure as for **8**. **10** BD, LRA, and LRC poses were merged into either MINA53 (PDB IDs: 4BXF and 2XDV) or NO66 (PDB ID: 4CCK) to provide the BD/4BXF, LRA/4BXF, LRC/2XDV, BD/4CCK, LRA/4CCK, and LRC/4CCK poses, which were subjected to MD simulation. The MM/GBSA results are consistent with the experimental observations revealing that **10** has a higher affinity for MINA53 over NO66 (Table S11). The best binding mode identified by calculations for **10** with MINA53 was BD (**10**_Tauto-1_), while for NO66, it was LRA (**10**_Tauto-1_). The large negative Δ*G* values for BD and LRA with MINA53 can substantially be ascribed to metal ion chelation. Interactions between **10**_Tauto-1_ and MINA53 include a hydrogen bond between Thr255 and the **10**_Tauto-1_ carboxylate, a π–π stacking of the **10**_Tauto-1_ pyrimidine ring with Tyr167, and chelation of the active site ion by the carboxylate and the carbonyl oxygen at pyrimidine 4 position of **10**_Tauto-1_ (Figure 5C). The predicted binding mode for **10**_Tauto-1_ with NO66 involves polar interactions involving Ser295, Lys355, and the **10**_Tauto-1_ carboxylate, hydrogen bonds of His417 and Lys355 with the carbonyl group at position 4 of the pyrimidine ring of **10**_Tauto-1_, and a π–π stacking of the phenyl ring of **10**_Tauto-1_ with Phe337 (Figure 5D). An RMSD analysis on the trajectories of the best **8** and **10** binding modes with MINA53 and NO66 (Figure S16) was performed. Ligand RMSD analysis (Figure S16C,D) clearly evidences the higher stability of the proposed binding modes of **8**_Tauto-1_ and **10**_Tauto-1_ on MINA53 over those on NO66, according to MM/GBSA calculations.

### Effects of MINA53 Inhibitors on Cancer Cell Lines

To examine the inhibition of MINA53 in a cellular context and investigate the use of MINA53 inhibitors as possible anticancer agents, we investigated: (1) the antiproliferative effect of MINA53 inhibitors in various cancer cells; (2) MINA53 targeting by substrate binding assays in cells; (3) the cellular selectivity of MINA53 inhibitors over specific KMDs; and (4) the ability of selected MINA53 inhibitors to sensitize cancer cells to conventional chemotherapy.

Since the role of MINA53 apparently depends on the cancer type,^3−11,17^ we first screened the most potent MINA53 inhibitors (**9**,**10**) and the inactive control (**1**) for antiproliferative effects against a panel of nine leukemia and lymphoma-derived cell lines (Table 2).

**Table 2:**
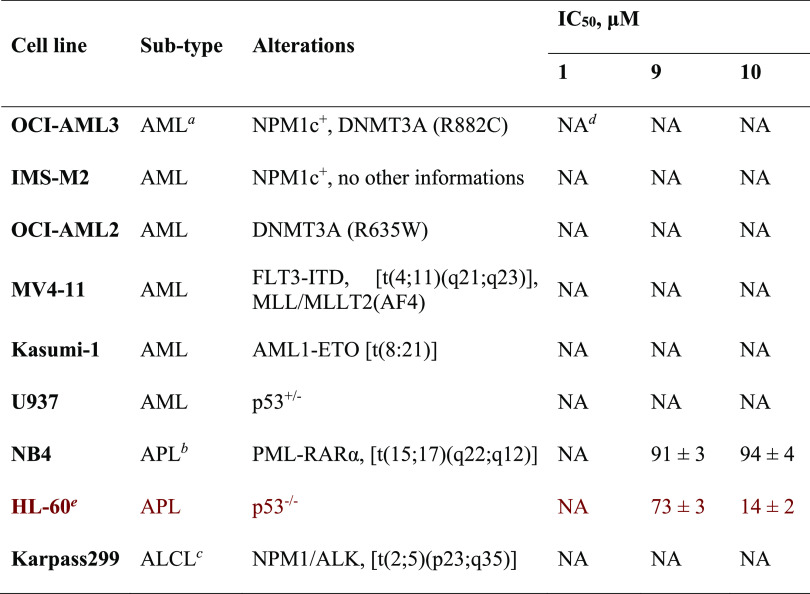
Antiproliferative Effect (IC_50_ Values) Displayed by **9** and **10** in a Panel of Leukemia and Lymphoma Cell Lines in Comparison with the Negative Control **1** Determined by the WST-1 Assay

a AML, acute myeloid leukemia.

b APL, acute promyelocytic leukemia.

c ALCL, anaplastic large cell lymphoma.

d NA, Not Active at the maximum tested dose of 100 μM.

e MINA53 regulates differentiation/proliferation of HL-60 leukemia-derived cells.^7^

**Figure 6:**
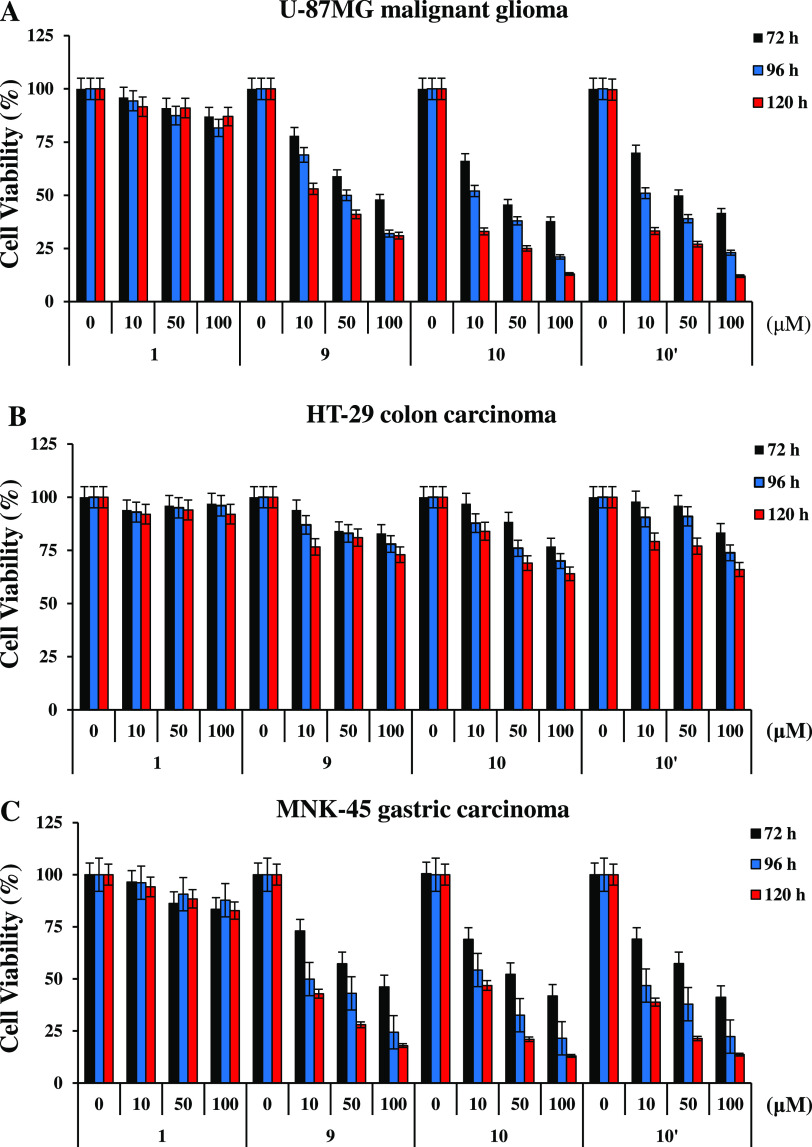
Effects of MINA53 inhibitors on the viability of solid cancer cells. U-87MG malignant glioma (A), HT-29 colon carcinoma (B), and MNK-45 gastric carcinoma (C) cell viability was determined by the MTT method after exposure for 72, 96, and 120 h to the negative control **1**, the MINA53 inhibitors **9** and **10**, and **10′**, the methyl ester of **10**. The results are reported as (viability of drug-treated cells/viability of control cells) × 100 and represent the mean ± SD of two independent experiments performed in triplicate.

**1** was inactive in all cases, as were **9** and **10** against seven of the lines. However, both **9** and **10** manifested antiproliferative activity against HL-60 cells and, to a lesser extent, NB4 acute promyelocytic leukemia-derived strains. Although further work is required to understand the determinants of sensitivity to **9** and **10**, these results are of interest because several studies have reported MINA53 upregulation in HL-60 cells.^2,7^ Because overexpression of *MINA53* has been reported in multiple solid cancers,^3,5,9,11,17^ we assessed the ability **9** and **10** to affect the cell viability of U-87MG malignant glioma, HT-29 colon, MNK-45 gastric, and HeLa cervical carcinoma cell lines. Cell viability assays, as determined by the MTT method, revealed that U-87MG (Figure 6A), MNK-45 (Figure 6C), and HeLa (Figure S17A) cells are sensitive to both **9** and **10**, whereas only a marginal effect was observed in HT-29 cells (Figure 6B). We also noted the sensitivity of human embryonic kidney cells HEK293T to both **9** and **10** (Figure S17B). To investigate whether the cellular performance of MINA53 inhibitors is affected by permeability issues, we tested the effects of the methyl ester of **10** (**10′**) on U-87MG, HT-29, and MNK-45 cell viability (Scheme 1 and Table S12). As reported in Figure 6, increasing the concentration of **10**′ reduced the cell viability of U-87MG, MNK-45, and HT-29 cells to a similar extent as that observed with **10**. Notably, the negative control **1** had no effect in any of the cell lines analyzed.

**Figure 7:**
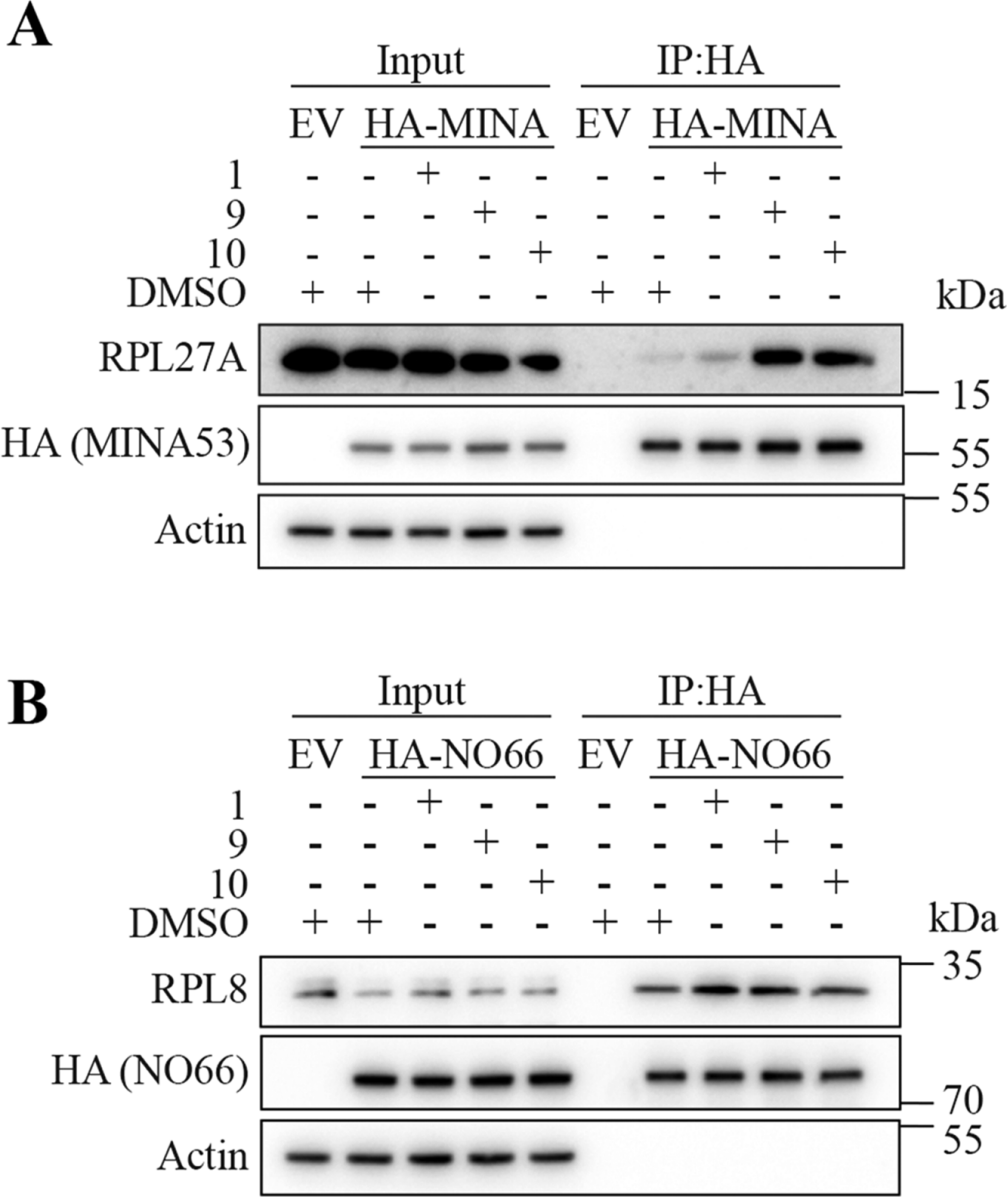
Immunoprecipitation analyses indicate MIN53 target engagement and selectivity of **9** and **10** in cells. Inhibitors **9** and **10** trigger “substrate trapping” of RPL27A by MINA53 (A) but not NO66 (B). HEK293T cells transiently expressing an empty (EV) HA-NO66 or HA-MINA53 vector were incubated with 100 μM **1**, **9**, and **10** or 0.5% (v/v) DMSO control for 18 h prior to anti-HA immunoprecipitation (IP). Anti-HA IPs were immunoblotted for the respective substrates RPL8 (28 kDa) and RPL27A (17 kDa).

Next, we explored whether **9** and **10** demonstrate any evidence for MINA53 target engagement and selectivity in cells. We first characterized the binding profile of recombinant HA-tagged MINA:RPL27A and NO66:RPL8 in several cell types in which we had observed effects of **9** and **10** on viability. The rationale underlying this approach is that modulating 2OG oxygenase activity often alters substrate interactions in cells. For example, Fe(II)-binding mutants can block the interaction with substrates,^38,39^ whereas small-molecule inhibitors can “trap” substrates.^39−41^ Interestingly, we observed that treating HEK293T (Figures 7A and S18A), HeLa (Figure S18B), and U-87MG (Figure S18C) cells with **9** and **10** led to increased binding of the endogenous RPL27A substrate to exogenous HA-tagged MINA53, as determined by immunoprecipitation analyses. These results are consistent with **9** and **10** engaging with the MINA53 active site “trapping” the substrate RPL27A. Importantly, in parallel experiments, we did not observe evidence of **9** and **10** trapping RPL8 with HA-tagged NO66 (Figure 7B), suggesting some level of selectivity for MINA53 over NO66 in cells, consistent with the analyses with isolated enzymes described above (Table 1).

**Figure 8:**
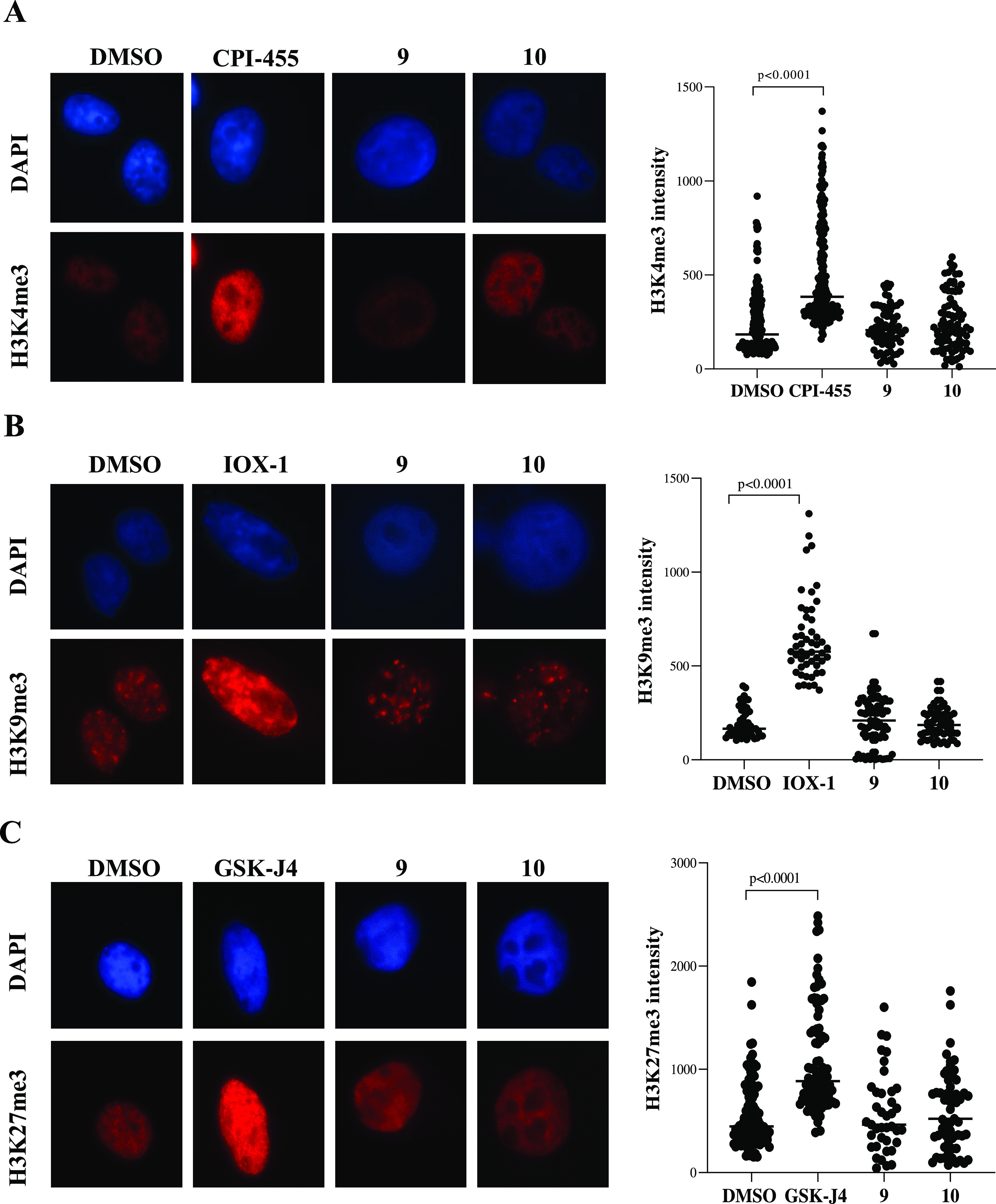
Cellular selectivity of **9** and **10** over representative KMDs. (A) Representative immunofluorescence analysis of the level of histone methyl mark H3K4me3 (left panel) and relative fluorescence intensity quantification in U-87MG cells treated with CPI-455 (25 μM, as a positive control for KDM5 inhibition) or with **9** and **10** (50 μM) for 48 h. (B) Representative immunofluorescence analysis of the level of histone methyl mark H3K9me3 (left panel) and relative fluorescence intensity quantification in U-87MG cells treated with IOX-1 (100 μM, as a positive control for KDM4 inhibition) or with **9** and **10** (50 μM) for 48 h. (C) Representative immunofluorescence analysis of the level of histone methyl mark H3K27me3 (left panel) and relative fluorescence intensity quantification in U-87MG cells treated with GSK-J4 (10 μM, as a positive control for KDM6 inhibition) or with **9** and **10** (50 μM) for 48 h. (A–C) Scatter plot (right side) illustrates the quantification of the specific histone methyl mark signal intensity in at least 50 counted cells per condition. The statistical analysis compares CPI-455 treatment vs DMSO control, IOX-1 treatment vs DMSO control, GSK-J4 treatment vs DMSO control (*p* < 0.0001, Mann Whitney test), and MINA53 inhibitor treatment vs DMSO control (no significance, Mann Whitney test).

To further investigate the cellular selectivity of **9** and **10**, we monitored their effects on levels of histone methyl marks H3K4me3 (using CPI-455 as a control for KDM5 inhibition),^42^ H3K9me3 (using IOX-1 as a control for KDM4 inhibition),^25^ and H3K27me3 (using GSK-J4 as a control for KDM6 inhibition)^26^ by immunofluorescence analysis in U-87MG cells. Following 48 h treatment with **9** and **10**, we did not observe any evidence for an increase of the methylation level of histone H3 at the specific lysine residues analyzed, suggesting that (at least under the tested conditions) neither **9** nor **10** alters histone H3 methylation status and that both possess cellular selectivity over KDM5 (Figure 8A), KDM4 (Figure 8B), and KDM6 (Figure 8C), respectively, at least in U-87MG cells.

Since silencing of MINA53 in glioblastoma cells results in DNA damage,^10^ we next tested the effects of **9**, **10**, and the inactive control **1** on H2AX serine 139 phosphorylation status (γ-H2AX) by Western Blot analysis after inhibitor treatment (Figure S19). γ-H2AX was not increased after exposure to **1**, but a strong γ-H2AX increase was observed in a time-dependent manner in U-87MG cells exposed to **9** or **10** at 20 μM.

**Figure 9:**
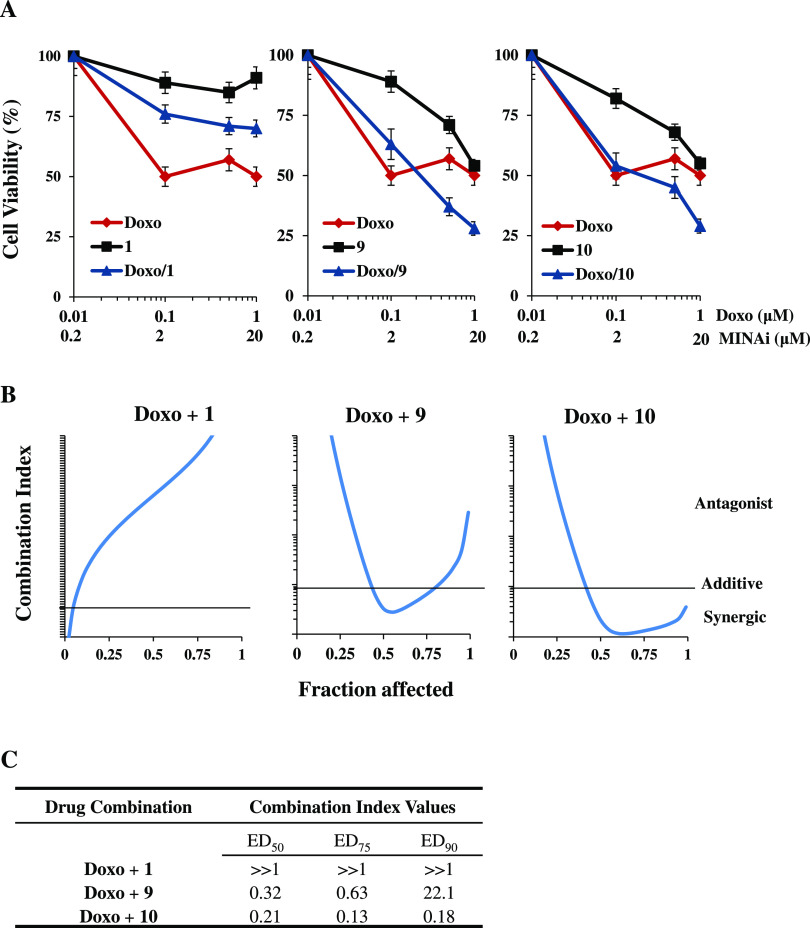
Evidence for synergism of the genotoxic drug doxorubicin in combination with **9** or **10** in human U-87MG glioma cells. (A) Analysis of cell viability by the 3-(4,5-dimethylthiazol-2-yl)-2,5-diphenyltetrazolium bromide (MTT) assay in U-87MG cells treated with doxorubicin (Doxo) and **9** and **10** alone or in combination (an inhibitor concentration ratio of 1:20) for 24 h, compared with the negative control **1**. The results are reported as (viability of drug-treated cells/viability of control cells) × 100 and represent the mean ± SD of the two independent experiments performed in triplicate. (B) Interaction between doxorubicin (Doxo) and **1**, **9**, and **10** (drug concentration ratio 1:20) evaluated on the basis of the combination index (CI), which is plotted against fractional growth inhibition. The results represent the mean of two independent experiments performed in triplicate. CI values < 0.9 (below the black line), 0.9–1.1 (around the black line), and >1.1 (above the black line) represent synergism, additivity, and antagonism effect, respectively. (C) Table showing the dose effects relationship of cell inhibition parameters and the CI value of **9** and **10** compared with **1**.

**Figure 10:**
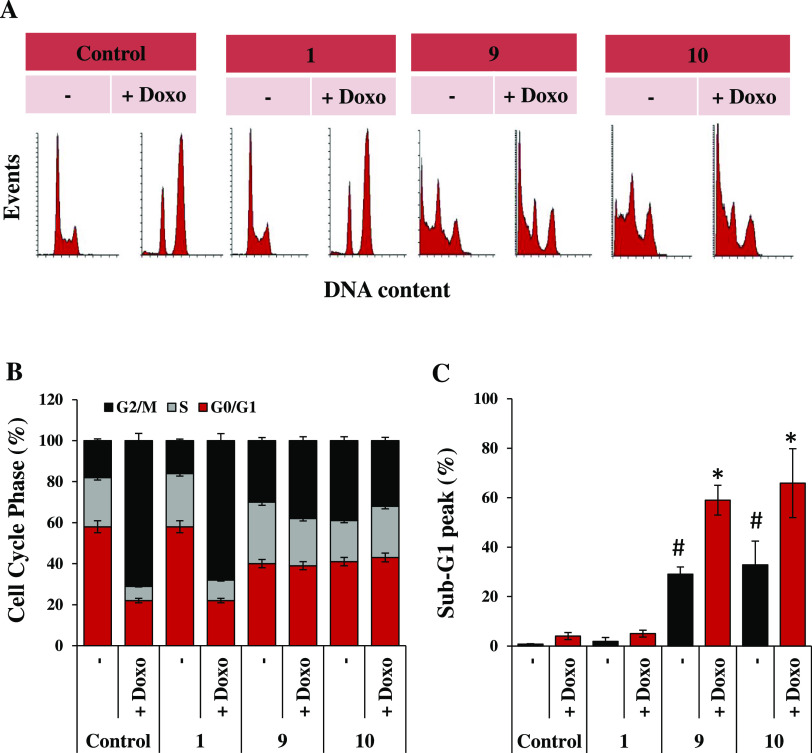
Proapoptotic effects of **9** and **10** in combination with doxorubicin on U-87MG cells. (A) Representative images of flow cytometric analysis of cell cycle distribution and the Sub-G1 peak by PI staining, in U-87MG cells untreated or treated with the negative control **1**, **9**, or **10** (10 μM), alone or in combination with doxorubicin (Doxo, 0.5 μM) for 24 h. Each panel is representative of three independent experiments with comparable results. Percentage of cells in different phases of the cell cycle (B) and at Sub-G1 peak (C) in U-87MG untreated or treated with the negative control **1**, **9**, or **10** (10 μM), alone or in combination with doxorubicin (Doxo, 0.5 μM) for 24 h. The statistical analysis compares MINA53 inhibitors vs DMSO control (# *p* < 0.05, *t*-test), and MINA53 inhibitor single treatment vs MINA53 inhibitors in combination with Doxo (* *p* < 0.05, *t*-test).

Based on these results and because MINA53 deficiency is reported to sensitize glioblastoma cells to the DNA damaging agent doxorubicin,^10^ we tested the effects of **9** or **10** in combination with doxorubicin on U-87MG cell viability, using **1** as a negative control. The results revealed dose-dependent sensitization of U-87MG cells to doxorubicin by both **9** and **10** (Figure 9). As expected, in contrast to **1**, single compound treatments of U-87MG cells with doxorubicin, **9** or **10** reduced cell viability in a dose-dependent manner. More importantly, the combination of **9** and **10** with doxorubicin provided a synergistic effect (the combination index value is less than 1). Indeed, a cell viability reduction of around 75% was observed using 1 μM doxorubicin in cotreatment with 20 μM of either **9** or **10** (Figure 9). Noteworthily, a combination index value higher than 1, indicative of an antagonist effect of the drug combination, was observed when cells were treated with the negative control **1** in combination with doxorubicin. We further characterized the biological effects of **1**, **9**, and **10** alone or in combination with doxorubicin on U-87MG cells after 24 h (Figure 10). Interestingly, treatment with 10 μM of **9** or **10**, but not **1**, induced accumulation of cells in the G2/M phase of the cell cycle (Figure 10A,B) and the appearance of a Sub-G1 peak, thus indicating the ability of **9** and **10** to induce apoptotic cell death in U-87MG (Figure 10A,C). Noteworthily, in line with cell viability response to drug combinations, the addition of doxorubicin (0.5 μM) to 10 μM of **9** or **10** doubled the percentage of apoptotic cells from about 30% to about 60%.

In summary, the cellular results presented support the proposal that **9** and **10** are cell-permeable compounds that can engage with their intended target, MINA53, in a manner that is selective over several other 2OG oxygenases, including its most closely related homologue NO66. Furthermore, our findings indicate that **9** and **10** reduce the viability of a range of tumor cell lines in isolation and in combination with the established chemotherapeutic agent doxorubicin, suggesting that further work investigating the molecular mechanisms involved is warranted.

## Conclusions

Our combined results validate the tractability of selective inhibition of the 2OG-dependent ribosomal oxygenases, and in particular, the cancer-linked enzyme MINA53, via targeting their Fe(II) containing catalytic site. Crystallographic analyses with KDM5B suggest an unprecedented mode of 2OG oxygenase inhibition in which C-2-substituted pyrimidine 5-carboxylic acids bind adjacent to the Fe(II) but do not directly chelate it; the inhibitors occupy the 2OG binding site and interact with the metal ion via water molecules. Molecular modeling investigations involving QM, molecular docking, and MD showed the pyrimidin-4-(3*H*)-one **10**_Tauto-1_ as the most likely tautomer for binding with MINA53 and NO66 (Figure 5C,D). Molecular docking and MD analysis indicated that pyrimidin-4-(3*H*)-ones **8**_Tauto-1_ and **10**_Tauto-1_ bind to MINA53 in a manner involving the direct metal ion interaction, while for both NO66 and KDM5B, this binding mode is not favored. These differences in the binding mode could account for the selective inhibition of MINA53 over NO66 by **10** in agreement with docking scores (Table S8) and the GB/SA predicted binding free energies (Table S11).

Further work is required to define the precise mode of MINA53 inhibition by 5-carboxy pyrimidin-4-(3*H*)-ones in solution. Correlation of our structure–activity relationship (SAR) with crystallographic analyses implies likely differences in the precise binding modes adopted by different C-2-substituted 5-carboxy pyrimidin-4-(3*H*)-ones at different JmjC subfamily 2OG oxygenase active sites as predicted by the molecular modeling investigations. Thus, in part because Fe(II) chelation may not be essential for inhibition by them, C-2-substituted 5-carboxy pyrimidin-4-(3*H*)-ones represent promising scaffolds for the development of highly selective 2OG oxygenase inhibitors.

Overall, despite the likely limitations in potency and selectivity of the first-generation MINA53 inhibitors described here, our combined observations are promising with respect to their potential to target MINA53 in cells. Our findings also raise the possibility that such MINA53 inhibitors could be of therapeutic benefit for cancer patients, including in combination with established chemotherapies. It should be noted, however, that successful clinical translation will benefit from the functional roles of MINA53 and ribosomal hydroxylation being defined more clearly in cells. Such studies will support our understanding of the context in which both normal and tumor cells may be sensitive to MINA53 loss of function and help in defining those tumor types where MINA53 inhibition may be beneficial. The role of MINA53 in cancer is complex, and thus the therapeutic value of MINA53 as a medicinal chemistry target should still be regarded as undemonstrated. However, we hope that the small-molecule MINA53 inhibitors described here, together with further optimized derivatives, will be of value in defining the biological roles and therapeutic tractability of MINA53. Our results suggest that the inhibition of the ribosomal oxygenases and, maybe, other translation machinery modifying enzymes (or indeed differentially modified ribosomes) is an approach meriting further investigation.

## Experimental Section

### Chemistry

Melting points were determined using a Buchi 530 melting point apparatus. ^1^H NMR and ^13^C NMR spectra were recorded at 400 and 100 MHz, respectively, with a Bruker AC 400 spectrometer, with chemical shifts in δ (ppm) units relative to the internal reference tetramethylsilane. All compounds were analyzed by thin-layer chromatography (TLC), ^1^H NMR, and ^13^C NMR. TLC was performed on aluminum-backed silica gel plates (Merck DC, Alufolien Kieselgel 60 F_254_) with spots visualized by UV light. Yields of all reactions refer to the purified products. All chemicals were from Sigma-Aldrich srl, Milan (Italy), and were of the highest available purity. Mass spectra were recorded with an API-TOF Mariner by a Perspective Biosystem (Stratford, TX); samples were injected by a Harvard pump using a flow rate of 5–10 μL/min with electrospray ionization. Elemental analysis was used to determine the purity of compounds, which in all cases was >95%; all analytical results were within ±0.40% of the theoretical values (Table S3). The purity of compounds **7**–**10** was also determined by HPLC (UV detection at λ = 254 nm) and was found to be higher than 97% (Figures S20–S23). The HPLC system consisted of a Waters 2695 (Waters, Milford, MA) chromatograph equipped with an automatic injector and a column heater and coupled with a model 996 PDA detector (Waters, Milford, MA). The analytical controls were performed on an Xterra RP_18_ 3.5 μm (3.9 mm × 100 mm) column (Waters, Milford, MA) in gradient elution. Eluents: (A) H_2_O/CH_3_CN, 95/5 + 0.05% formic acid and (B) CH_3_CN/H_2_O, 95/5 + 0.05% formic acid. Gradient profile: start A/B 90/10, in 15 min 100% B, 20 min 100% B. Flow rate: 1.0 mL/min at room temperature. Samples **7** and **8** were dissolved in MeOH/DMSO 9/1, while samples **9** and **10** in MeOH at *c*: 1 mg/mL. Injection volume: 3 μL.

#### Synthesis of 2-(Methylthio)-3,4-dihydro-4-oxopyrimidine-5-carboxylic Acid (**2**)

Methyl iodide (11.6 mmol, 2 equiv) was added to a solution of commercial **1** (5.81 mmol, 1 equiv) in dry DMF (3 mL); the reaction mixture was stirred at room temperature for 26 h. Upon the conclusion of the reaction, the mixture was quenched with water (30 mL) and the resulting suspension was filtered under vacuum. The solid on the filter was washed with dried THF and then recrystallized from methanol to provide **2**. ^1^H NMR (400 MHz; DMSO-*d*_6_) δ 2.52 (s, 3H, SC*H*_3_), 8.50 (s, 1H, C*H* pyrimidine proton). ^13^C NMR (100 MHz, DMSO-*d*_6_) δ 13.69, 109.72, 156.29, 165.09, 166.40, 168.30. MS (ESI), *m*/*z*: 185 [M – H]^−^.

#### General Procedure for the Synthesis of Compounds **3**–**11**

Anhydrous potassium carbonate (12.8 mmol, 2.2 equiv) and the requisite (cyclo)alkyl-/arylalkyl bromide (6.4 mmol, 1.1 equiv) were added to a solution of **1** (5.81 mmol, 1 equiv) in dry DMF (4 mL). The resulting mixture was stirred at room temperature for 24 h. Upon the conclusion of the reaction, the mixture was poured into water (100 mL) and extracted with ethyl acetate (5 × 20 mL). Then, 12 N hydrochloric acid was added dropwise at 0 °C to the aqueous phase, and the resulting precipitate was isolated by filtration, washed over filter with water, and recrystallized from the appropriate solvent system to give **3–11**.

#### 2-(Butylthio)-3,4-dihydro-4-oxopyrimidine-5-carboxylic Acid (**3**)

^1^H NMR (400 MHz; CDCl_3_) δ 0.94 (t, 3H, S(CH_2_)_3_C*H*_3_), 1.45 (m, 2H, S(CH_2_)_2_C*H*_2_CH_3_), 1.68–1.75 (m, 2H, SCH_2_C*H*_2_CH_2_CH_3_), 3.30 (t, 3H, SC*H*_2_(CH_2_)_2_CH_3_), 8.82 (s, 1H, C*H* pyrimidine proton), 12.12–12.38 (br m, 2H, N*H* and COO*H*). ^13^C NMR (100 MHz, DMSO-*d*_6_) δ 13.90, 21.72, 30.31, 31.00, 109.84, 156.92, 165.06, 166.11, 167.80. MS (ESI), *m*/*z*: 227 [M – H]^−^.

#### 2-(Pentylthio)-3,4-dihydro-4-oxopyrimidine-5-carboxylic Acid (**4**)

^1^H NMR (400 MHz; DMSO-*d*_6_) δ 0.86 (t, 3H, S(CH_2_)_4_C*H*_3_), 1.26–1.36 (m, 4H, S CH_2_CH_2_(C*H*_2_)_2_CH_3_), 1.65 (m, 2H, SCH_2_C*H*_2_(CH_2_)_2_CH_3_), 3.17 (t, 2H, SC*H*_2_(CH_2_)_3_CH_3_), 8.51 (s, 1H, C*H* pyrimidine proton). ^13^C NMR (100 MHz, DMSO-*d*_6_) δ 14.25, 22.07, 28.62, 30.57, 30.70, 109.78, 156.24, 166.35, 167.85. MS (ESI), *m*/*z*: 241 [M – H]^−^.

#### 2-(Nonylthio)-3,4-dihydro-4-oxopyrimidine-5-carboxylic Acid (**5**)

^1^H NMR (400 MHz; DMSO-*d*_6_) δ 0.86 (t, 3H, S-(CH_2_)_8_C*H*_3_), 1.25 (br m, 10H, S(CH_2_)_3_(C*H*_2_)_5_CH_3_), 1.37 (m, 2H, S(CH_2_)_2_C*H*_2_(CH_2_)_5_CH_3_), 1.64–1.66 (m, 2H, SCH_2_C*H*_2_(CH_2_)_6_CH_3_), 3.19 (t, 2H, SC*H*_2_(CH_2_)_7_CH_3_), 8.52 (s, 1H, C*H* pyrimidine proton). ^13^C NMR (100 MHz, DMSO-*d*_6_) δ 14.41, 22.56, 28.5, 28.92, 28.94, 29.09, 29.30, 30.58, 31.73, 109.94, 156.74, 165.10, 165.81, 167.88. MS (ESI), *m*/*z*: 297 [M – H]^−^.

#### 2-((Cyclohexylmethyl)thio)-3,4-dihydro-4-oxopyrimidine-5-carboxylic Acid (**6**)

^1^H NMR (400 MHz; DMSO-*d*_6_) δ 0.92–1.26 (m, 5H, 5xC*H* cyclohexane), 1.57–1.79 (m, 6H, 6xC*H* cyclohexane), 3.12 (d, 2H, SC*H*_2_-cyclohexane), 8.51 (s, 1H, C*H* pyrimidine proton). ^13^C NMR (100 MHz, DMSO-*d*_6_) δ 25.86 (2C), 26.18, 32.27 (2C), 37.17, 37.27, 109.73, 155.53, 165.03, 167.05 167.86. MS (ESI), *m*/*z*: 267 [M – H]^−^.

#### 2-(Benzylthio)-3,4-dihydro-4-oxopyrimidine-5-carboxylic Acid (**7**)

^1^H NMR (400 MHz; DMSO-*d*_6_) δ 4.49 (s, 2H, SC*H*_2_Ph), 7.24–7.36 (m, 3H, phenyl protons), 7.43–7.45 (m, 2H, phenyl protons), 8.57 (s, 1H, C*H* pyrimidine proton). ^13^C NMR (100 MHz, DMSO-*d*_6_) δ 34.44, 108.88, 127.75, 128.96 (2C), 129.51 (2C), 137.70, 156.54, 166.61, 169.09, 169.74. MS (ESI) *m*/*z*: 261 [M – H]^−^.

#### 2-((2-Oxo-2-phenylethyl)thio)-3,4-dihydro-4-oxopyrimidine-5-carboxylic Acid (**8**)

^1^H NMR (400 MHz; DMSO-*d*_6_) δ 4.95 (s, 2H, SC*H*_2_COPh), 7.52–7.62 (m, 2H, phenyl protons), 7.69–7.73 (m, 1H, phenyl proton), 8.03–8.07 (m, 2H, phenyl protons), 8.45 (s, 1H, C*H* pyrimidine proton). ^13^C NMR (100 MHz, DMSO-*d*_6_) δ 39.19, 110.04, 128.83 (2C), 129.35 (2C), 134.25, 135.98, 156.29, 165.04, 167.14, 168.40, 192.85. MS (ESI), *m*/*z*: 289 [M – H]^−^.

#### 2-(Phenethylthio)-3,4-dihydro-4-oxopyrimidine-5-carboxylic Acid (**9**)

^1^H NMR (400 MHz; DMSO-*d*_6_) δ 2.98 (t, 2H, SCH_2_C*H*_2_Ph), 3.45 (t, 2H, SC*H*_2_CH_2_Ph), 7.23–7.25 (m, 1H, phenyl proton), 7.28–7.34 (m, 4H, phenyl protons), 8.56 (s, 1H, C*H* pyrimidine proton). ^13^C NMR (100 MHz, DMSO-*d*_6_) δ 31.93, 34.82, 110.36, 126.95, 128.89 (2C), 129.10 (2C), 140.05, 155.60, 165.10, 166.09, 167.58. MS (ESI), *m*/*z*: 275 [M – H]^−^.

#### 2-((3-Phenylpropyl)thio)-3,4-dihydro-4-oxopyrimidine-5-carboxylic Acid (**10**)

^1^H NMR (400 MHz; DMSO-*d*_6_) δ 1.94 (m, 2H, SCH_2_C*H*_2_CH_2_Ph), 2.66 (t, 2H, S(CH_2_)_2_C*H*_2_Ph), 3.16 (t, 2H, SC*H*_2_(CH_2_)_2_Ph), 7.13–7.27 (m, 5H, phenyl protons), 8.48 (s, 1H, C*H* pyrimidine proton), 13.02 (br s, 1H, O*H*). ^13^C NMR (100 MHz, DMSO-*d*_6_) δ 30.21, 30.54, 34.45, 109.86, 126.42, 128.79 (2C), 128.83 (2C), 141.37, 155.68, 165.08, 166.42, 167.71. MS (ESI), *m*/*z*: 289 [M – H]^−^.

#### 2-((4-Phenylbutyl)thio)-3,4-dihydro-4-oxopyrimidine-5-carboxylic Acid (**11**)

^1^H NMR (400 MHz; DMSO-*d*_6_) δ 1.66 (m, 4H, SCH_2_(C*H*_2_)_2_CH_2_Ph), 2.61 (m, 2H, S(CH_2_)_3_C*H*_2_Ph), 3.02 (t, 2H, SC*H*_2_(CH_2_)_3_Ph), 7.19–7.25 (m, 5H, phenyl protons), 8.60 (s, 1H, C*H* pyrimidine proton). ^13^C NMR (100 MHz, DMSO-*d*_6_) δ 28.39, 30.21, 30.54, 34.95, 111.33, 126.42, 128.79 (2C), 128.83 (2C), 141.74, 155.66, 165.08, 166.20, 167.71. MS (ESI), *m*/*z*: 303 [M – H]^−^.

#### Procedure for the Synthesis of Methyl 2-((3-phenylpropyl)thio)-3,4-dihydro-4-oxopyrimidine-5-carboxylate (**10′**)

A solution of **10** (290 mg, 1 mmol, 1.0 equiv) in aqueous methanol (10 mL) was treated with cesium carbonate (0.5 equiv, 0.5 mmol, 96 mg). The resulting solution was stirred at room temperature for 45 min and then evaporated at reduced pressure and co-evaporated with toluene (3 × 10 mL). The resulting white cesium salt was suspended in dry DMF (5 mL), cooled to 0 °C, and treated with methyl iodide (1.0 equiv, 1 mmol, 141.9 mg, 62 μL). After 1 h stirring at 0 °C, the solution was allowed to warm to room temperature and stirring was continued for a further 2 h before the solvent was removed under reduced pressure. The residue was then taken up into ethyl acetate (60 mL) and washed with brine (4 × 5 mL). The organic phase was then dried over anhydrous sodium sulfate, and the solvent was removed under reduced pressure. Finally, product **10′** was obtained as a pure white powder by silica gel column chromatography purification of the crude residue using the mixture of hexane:ethyl acetate:methanol 10:5:0.5 v/v/v as an eluent system. ^1^H NMR (400 MHz; DMSO-*d*_6_) δ 1.97 (m, 2H, SCH_2_C*H*_2_CH_2_Ph), 2.70 (t, 2H, S(CH_2_)_2_C*H*_2_Ph), 3.19 (t, 2H, SC*H*_2_(CH_2_)_2_Ph), 3.76 (s, 3H, COOC*H*_3_), 7.16–7.30 (m, 5H, phenyl protons), 8.51 (s, 1H, C*H* pyrimidine proton). ^13^C NMR (100 MHz, DMSO-*d*_6_) δ 30.21, 30.54, 34.45, 51.54, 109.86, 126.42, 128.79 (2C), 128.83 (2C), 141.37, 155.60, 165.08, 166.34, 167.71. MS (ESI), *m*/*z*: 305 [M+H]^+^.

#### Procedure for the Synthesis of (*Z*)-2-((3-Ethoxy-3-oxoprop-1-en-1-yl)thio)-3,4-dihydro-4-oxopyrimidine-5-carboxylic Acid (**12**)

Ethyl propiolate (3.48 mmol, 1.2 equiv) and a solution of tetrabutylammonium fluoride in THF (7.00 mmol, 2.4 equiv) were added to a solution of **1** (2.9 mmol, 1 equiv) in dry THF (9 mL). The resulting mixture was stirred at room temperature for 22 h. Upon the conclusion of the reaction, the resulting mixture was concentrated in vacuo and the crude reaction mixture was poured into a saturated sodium carbonate solution (20 mL) and then extracted with ethyl acetate (4 × 5 mL). Then, 12 N hydrochloric acid was added dropwise at 0 °C to the aqueous phase. The resulting precipitate was isolated by filtration, washed with water, and recrystallized from a mixture of toluene/acetonitrile to provide compound **12**. ^1^H NMR (400 MHz; DMSO-*d*_6_) δ 1.21 (t, 3H, COOCH_2_C*H*_3_), 4.10 (q, 2H, COOC*H*_2_CH_3_), 6.30 (d, 1H, SCH = C*H*-COOCH_2_CH_3_, *J*_cis_ = 8 Hz), 8.34 (d, 1H, SC*H* = CHCOOH, *J*_cis_ = 8 Hz), 8.57 (s, 1H, C*H* pyrimidine proton). ^13^C NMR (100 MHz, DMSO-*d*_6_) δ 15.05, 61.51, 110.96, 117.22, 139.83, 157.22, 160.03, 166.24, 166.79, 167.78. MS (ESI), *m*/*z*: 269 [M – H]^−^.

#### Procedure for the Synthesis of (*Z*)-2-((2-carboxyvinyl)thio)-3,4-dihydro-4-oxopyrimidine-5-carboxylic Acid (**13**)

Potassium hydroxide (2 N) (3.7 mmol, 10 equiv) was added dropwise to a solution of **12** (0.37 mmol, 1 equiv) in ethanol (3.5 mL). The resulting reaction mixture was stirred at room temperature for 24 h. Upon the completion of the reaction, the solvent was concentrated in vacuo and the resulting basic phase was diluted with water (5 mL) and extracted with ethyl acetate (5 × 1 mL). Then, 12 N hydrochloric acid was added dropwise at 0 °C to the aqueous phase, and the resulting white precipitate was isolated by filtration, washed over filter with water, and recrystallized from a mixture of acetonitrile/methanol to provide **13**. ^1^H NMR (400 MHz; DMSO-*d*_6_) δ 6.29 (d, 1H, SCH = C*H*COOH, *J*_cis_ = 8 Hz), 8.34 (d, 1H, SC*H* = CHCOOH, *J*_cis_ = 8 Hz), 8.63 (s, 1H, C*H* pyrimidine proton), 13.11 (br s, 1H, O*H*). ^13^C NMR (100 MHz, DMSO-*d*_6_) δ 102.18, 119.62, 151.12, 151.32, 164.44, 166.14, 168.23, 171.22. MS (ESI), *m*/*z*: 241 [M – H]^−^.

### Production of Recombinant NO66 and MINA53

Constructs for bacterial production of recombinant NO66 and MINA53 were obtained from the structural genomics consortium (SGC). In brief, DNA sequences encoding for NO66 (Ala167-Asn641 and Ser183-Asn641) and MINA53 (Met1-Val464 and Ala26-Val464) were subcloned into the pNIC28-Bsa4 and pNIC-CTHF vectors to enable the production of the recombinant protein with Tobacco Etch Virus (TEV) protease cleavable *N*- and *C*-terminal histidine tags, respectively. The plasmids were transformed using standard protocols^43^ into the BL21(DE3)-R3-pRARE2 cell line, a phage-resistant derivative of BL21(DE3) carrying a pRARE2 plasmid to enable expression of eukaryotic proteins that contain codons rarely used in *Escherichia coli*.^44^

Overnight cultures (100 mL) were prepared from glycerol stocks or freshly transformed cells using terrific broth (TB) supplemented with 100 μg/mL kanamycin. Then, 10 mL of overnight culture was added per 1 L TB supplemented with 100 μg/mL kanamycin and 4 mL of glycerol. The cultures were incubated (160 rpm, 37 °C) until an average OD600 of 2.5 was reached. The temperature was then reduced to 18 °C for 30 min, prior to induction with 0.1 mM IPTG (isopropyl-β-d-thiogalactopyranoside) at 18 °C overnight. The cells were harvested by centrifugation (3000*g*, 20 min), and the supernatant was discarded. The cell pellet was either frozen at −20 °C or used immediately. Cell pellets from a 1 L scale were resuspended in 50 mL of buffer containing 50 mM 4-(2-hydroxyethyl)-1-piperazineethanesulfonic acid (HEPES) (pH 8.0), 500 mM NaCl, 10 mM imidazole, 0.5 mM tris(2-carboxyethyl)phosphine (TCEP), and 5% (v/v) aqueous glycerol, supplemented with 1 μL of benzonase nuclease (Sigma-Aldrich) and 50 μL of protease inhibitors cocktail III (Merck).

The cells were disrupted by sonication followed by centrifugation (45 min, 23 800*g*), and the supernatant was collected. Proteins were purified by nickel affinity chromatography at 4 °C, using a gravity column and a stepwise gradient of imidazole. Fractions were analyzed by 4–12% sodium dodecyl sulfate polyacrylamide gel electrophoresis (SDS-PAGE). Fractions were pooled and concentrated using an Amicon 30 kDa cut-off concentrator to 5 mL and then filtered through a 0.22 μm poly(vinylidene fluoride) (PVDF) filter. The solutions were then injected manually onto a size-exclusion chromatography column (S200, Pharmacia) using an ÄKTA Xpress machine equilibrated with 10 mM HEPES (pH 7.5), 500 mM NaCl (or 150 mM NaCl for MINA53), 5% (v/v) aqueous glycerol, and 0.5 mM TCEP. The fractions from the S200 column were analyzed using 4–12% SDS-PAGE. Affinity tags were removed using the TEV protease (30 μg per mg of protein) with overnight incubation at 4 °C. The TEV protease and uncleaved recombinant protein were removed by Ni-Sepharose chromatography. The mass of the cleaved proteins was verified by electrospray mass ionization time-of-flight mass spectrometry (Agilent LC/MSD). The TEV cleaved proteins were concentrated using Amicon 30 kDa spin concentrators. Protein concentrations were determined using a NanoDrop machine (Thermo Scientific) using the absorption at 280 nm and the estimated extinction coefficient.

### MINA53 and NO66 Mass Spectrometry Activity Assay

Assays used an Rpl8 peptide substrate for NO66 (Asn205-Thr224, 20-mer; NPVEHPFGGGNHQHIGKPST) and an Rpl27a peptide substrate for MINA53 (Gly31-Pro49, 20-mer; GRGNAGGLHHHRINFDKYHP) synthesized by Peptide Synthetics (Fareham, Hampshire, U.K.). Ferrous ammonium sulfate (FAS) as its hexahydrate, l-ascorbic acid (l-AA), and 2OG were from commercial sources, and their solutions were prepared fresh daily. All assay samples were analyzed by solid-phase extraction mass spectrometry (SPE-MS) using a RapidFire RF360 high throughput sampling robot (Agilent) coupled to a 6530 accurate mass Q-TOF (Agilent). The assays for NO66 and MINA53 were performed using optimized buffers (Table S4). Time-course assays for NO66 and MINA53 at final assay concentrations for NO66 (0.3 mM) and MINA53 (0.15 mM) were carried out in 96 2 mL deep-well plates (Greiner Bio-one). Reactions were initiated by the addition of 0.25 mL of NO66 (1.5 mM) or MINA53 (0.75 mM) to 1.0 mL of substrate (12.5 mM 2OG, 125 mM l-AA, 12.5 mM FAS, 12.5 mM substrate). Hydroxylation (+16 Da) of substrates was monitored at room temperature in real-time by programming the RapidFire sampling robot to sample the enzyme reaction at two-minute intervals over a time course of 35 min to generate a progress curve. The samples were aspirated under vacuum onto a C4 SPE cartridge on a RapidFire RF360 high throughput sampling robot, and the C4 cartridge was washed for 5500 ms with an aqueous solvent (0.1% (v/v) formic acid in LC-MS grade water) to remove nonvolatile buffer salts. Substrates and hydroxylated products were eluted from the C4 SPE cartridge with an organic solvent (85% (v/v) acetonitrile in water, 0.1% (v/v) formic acid) for 6000 ms. MS analysis employed an Agilent 6530 accurate mass Q-TOF. The sample sipper was washed with deionized water and 100% acetonitrile, and the SPE cartridge was washed with aqueous wash buffer to remove cross-contamination between sample injections. We observed maximum signals for charge state *m*/*z* +4 with Rpl8 and *m*/*z* +5 with Rpl27a. The ion chromatography data for these two charge states were extracted for both hydroxylated and nonhydroxylated peptides, and peak areas were integrated using Agilent RapidFire Integrator v. 3.6. Integrated data were exported to Excel, and the percent conversion to the hydroxylated product was calculated using the following formula
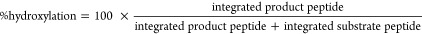
Enzyme progress curves were plotted in excel; nonlinear curve fitting was performed with the Michaelis–Menten equation for kinetics studies and sigmoidal dose–response curve with a variable slope for the determination of IC_50_ values.

### Determination of Enzyme Kinetic Parameters for NO66 and MINA53

Substrate *K*_M_ and *V*_max_ values were determined from the initial rate slope of progress curves generated with 1, 2, 4, 5, 6, 8, 10, 12, 15, and 20 μM concentrations of the Rpl27a peptide for MINA53, and 2, 4, 5, 6, 8, 10, 12, 15, 20, 30, 40, and 50 μM of the Rpl8 peptide for NO66. Assays were performed in a 96-well, 2 mL polypropylene plate (Greiner Bio-one). Reactions were initiated by the addition of 10 μL of 100× concentrated enzyme (30 μM for NO66 and 15 μM for MINA53) to 1000 μL of substrate (50 μM FAS, 100 μM l-AA, 50 μM 2OG, 1–50 μM Peptide). All reactions were performed using optimized buffer conditions (Table S4). The samples were aspirated from each well by a RapidFire RF365 high throughput sampling robot connected to an Agilent 6550 accurate mass Q-TOF every 3 min over a time course of 50 min. Ion chromatogram data for the Rpl27a (*m*/*z* +5 charge state) and Rpl8 substrates (*m*/*z* +4 charge state) were extracted, and the peak areas were integrated using RapidFire Integrator software (Agilent). The integrated peak areas were exported into Excel, and the percent hydroxylation was calculated. The initial rate slope for each peptide concentration was calculated using excel, and the data were fitted to the Michaelis–Menten equation using GraphPad prism 5.

*K*_M_ and *V*_max_ values for 2OG and Fe^2+^ were determined by generating progress curves at 8 concentrations (four replicates). The concentrations were prepared as an 8-point 2-fold dilution series with a starting concentration of 20 μM. Each time series was based on 6-time points (0, 1, 5, 10, 20, and 30 min for NO66 and 0, 1, 3, 5, 7, and 10 min for MINA53). Assays were performed in 96-well plates (Greiner Bio-one); reactions were initiated by the addition of 10 μL of concentrated enzyme solution (3000 nM of NO66 or 1500 nM of MINA53) in respective assay buffer to 40 μL of peptide solution (500 μM l-AA and 12.5 μM peptide) in respective assay buffer with an excess of 2OG (62.5 μM) and FAS (125 μM). Reactions were stopped by the addition of 5 μL of 10% (v/v) formic acid. The first four reaction data points (starting with the 1 min time point) were used for kinetic analysis.

### IC_50_ Determinations

Compounds for dose–response or single-concentration experiments were dispensed using an Echo 550 acoustic dispenser (Labcyte, Sunnyvale, CA) into 384-well HiBase plates (Greiner Bio-one) or 96-well plates (Greiner Bio-one) with the last two columns of the plate comprising 1% (v/v) DMSO control and 100 mM 2,4-PDCA (pyridine-2,4-dicarboxylate) control, respectively. The plate was backfilled to a total volume of 500 nL of DMSO. NO66 was diluted to 375 nM in 50 mM MES (pH 7.0) and 150 mM NaCl. MINA53 was diluted to 187.5 nM in 50 mM HEPES (pH 7.5) and 50 mM NaCl. Assays were performed in the following order: (1) the addition of 40 μL of enzyme solution to compounds in the desired plate format; (2) 15 min incubation at room temperature with compounds; (3) reaction initiation by the addition of 10 μL of peptide substrate solution [250 μM ferrous ammonium sulfate (FAS), 500 μM l-AA 25 μM 2OG, and 25 μM peptide in the respective assay buffer]; (4) 30 or 60 min assay incubation with MINA53 or NO66, respectively; and (5) reactions were stopped by the addition of 5 μL of 10% (v/v) formic acid. Reaction plates were either analyzed immediately using a RapidFire RF360 mass spectrometer (Agilent Scientific) or frozen at −20 °C. For the IC_50_ or single-concentration inhibition studies, the data were normalized to a 100 μM 2,4-PDCA positive control and the % inhibition was calculated in Microsoft Excel according to the formula
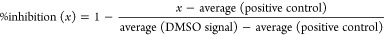
IC_50_s were determined from nonlinear regression curve fits using GraphPad Prism 5.0.

### AlphaScreen KDM Inhibition Assays and Counter Screen

Counter-screening for AlphaScreen interference was carried out using the AlphaScreen General IgG (Protein A) detection kit (Perkin Elmer). Inhibitors were preincubated with a 10 nM biotinylated substrate, 10 μM FAS, 10 μM 2OG, and 100 μM l-AA in AlphaScreen buffer for 20 min, then incubated with AlphaScreen beads with antibodies. Luminescence was detected after 1 h.

AlphaScreen inhibition assays were carried out as reported.^45,46^ Details on the individual assay conditions are in Table S5. Purified proteins were available at the SGC as reported.^47^ The AlphaScreen General IgG detection kit was from Perkin Elmer. Assays were carried out in 384-well Proxiplates plus from Perkin Elmer. HEPES buffer was purchased from Life Technologies, FAS (from Riedel-de Haen) was diluted in 20 mM HCl to 400 mM concentration and then to 1 mM in deionized water. Bovine serum albumin (BSA) was from Sigma-Aldrich (A7030). l-AA and 2OG were dissolved in deionized water and were from Sigma-Aldrich. FAS, 2OG, and l-AA were prepared each day freshly. Assays were performed at room temperature (25 °C); compounds were dissolved in DMSO. Compounds were dispensed using an Echo 550 Acoustic Dispenser (Labcyte). Then, 5 μL of 2× enzyme solution in assay buffer was added to the plates and incubated for 15 min at room temperature. Five microlitres of 2× substrate was prepared in assay buffer with l-AA, 2OG, and FAS, with the concentrations in Table S5, and added to the plate to start the reaction. Reactions were incubated as described in Table S5 and stopped by the addition of a stop solution (30 mM EDTA, 800 mM NaCl in assay buffer). Then, 5 μL of AlphaScreen beads previously incubated with the appropriate antibody (Table S5) was added using a multichannel pipette; plates were left to incubate for at least 2 h. Luminescence was detected using a BMG Labtech Pherastar FS plate reader. Data were normalized to a positive control (100 μM of 2,4-PDCA) and analyzed using GraphPad Prism. IC_50_ calculations were determined from nonlinear regression curve fitting using GraphPad Prism 5.0.

### RapidFire FIH Inhibition Assay^28^

The reported procedure was used.^28^ Tris(hydroxymethyl)aminomethane was from Fisher; all other reagents were from Sigma-Aldrich and of the highest available purity. FAS was prepared freshly every day as a 400 mM stock solution in 20 mM HCl, and this was then diluted to 1 mM in deionized water. l-AA (50 mM) and 2OG stock solutions (10 mM) were prepared freshly every day in deionized water. The synthetic “consensus” ankyrin repeat derived peptide (HLEVVKLLLEAGADVNAQDK-CONH_2_) was synthesized by GL Biochem (Shanghai) Ltd and dissolved to 1 mM in deionized water. Then, 20 μL of FIH (100 nM) in the assay buffer (50 mM Tris.Cl (pH 7.8), 50 mM NaCl) was preincubated for 15 min in the presence of the inhibitors and the enzyme reaction was initiated by the addition of 20 μL of substrate (200 μM l-AA, 20 μM Fe(II), 10 μM synthetic ankyrin peptide, and 20 μM 2OG). After 15 min, the reaction was quenched by the addition of 4 μL of 10% (v/v) aqueous formic acid and the reaction was transferred to a RapidFire RF360 high throughput sampling robot. The samples were aspirated under vacuum onto a C4 solid-phase extraction (SPE) cartridge. After an aqueous washing step (0.1% formic acid), to remove nonvolatile buffer components from the C4 SPE cartridge, peptides were eluted in an organic washing step (85% (v/v) aqueous acetonitrile, 0.1% formic acid) and injected into an Agilent 6530 Q-TOF mass spectrometer. Ion chromatogram data were extracted for the nonhydroxylated peptide substrate and the hydroxylated peptide product; peak area data for the extracted ion chromatograms were integrated using RapidFire Integrator software. Percentage conversion of the substrate to the product was calculated in Microsoft excel, and IC_50_ curves were generated using GraphPad Prism 5.0.

### Crystallization and Structure Determination of KDM5B

Purified truncated KDM5B (Phe26-Leu101-GlyGlyGlyGly-Ala374-Ile502) was prepared as previously reported.^33^ KDM5B was crystallized using the 3-drop sitting drop vapor diffusion method at 4 °C in 0.1 M HEPES (pH 7.5), 0.8 M potassium phosphate dibasic, 0.8 M sodium phosphate monobasic supplemented with 2 mM MnCl_2_. Crystallization drops (150 nL or 300 nL) were set up with a mosquito robot (TTP Labtech) using a protein concentration of 8–9 mg/mL in 2:1, 1:1, and 1:2 protein to precipitant ratios, with and without the addition of 20 nL of 1:100 (v/v) dilution of a seed stock. Then, 30–100 crystals were reproducibly grown in Swiss CI plates (SWISSCI, Neuheim, Switzerland) in the uniform conditions described above. Crystals were flash-frozen in liquid nitrogen at 100 K. Data were collected at Diamond Light Source beamline I03. Data were processed using Xia2,^48^ followed by molecular replacement and initial refinement in the DIMPLE pipeline^49^ based on the PDB ID: 5A1F. Iterative model building with COOT^50^ and refinement with PHENIX^51^ and BUSTER v2.10.1 resulted in final models. Data collection and refinement statistics are in Supporting Information Table S6.

### Molecular Modeling Studies

All molecular modeling details are reported in the Supporting Information.

### Cell Cultures, Treatment, and Analysis of Cell Viability

The effect of compounds on cell proliferation of different leukemia and lymphoma cell lines OCI-AML3 (DSMZ ACC-582), IMS-M2 (a gift from Professor B. Falini, University of Perugia), OCI-AML2 (DSMZ, ACC-99), MV4–11 (ATCC, CRL-9591), Kasumi-1 (ATCC, CRL-2724), Karpass299 (DSMZ, ACC-31), U937 (ATCC, CRL1593.2), HL-60 (ATCC, CCL-240), and NB4 (DSMZ, ACC-207) was evaluated using the WST-1 assay (Roche Diagnostic GmbH Germany). The cells were plated, in triplicate, in 384-viewplate (Perkin Elmer) at a density of 5 × 10^3^ cells/well in a final volume of 22.5 μL. The cells were incubated with increasing concentrations of compounds for 48 h at 37 °C, 5% CO_2_, then 2.5 μL of WST-1 reagent was added to each well for 1 h. For each cell line, dose–response curves were analyzed as nonlinear regression curves using GraphPad Prism (GraphPad Software Inc.) to obtain IC_50_ values.

Human commercially available established cancer cell lines HT-29 colon carcinoma (ATCC, HTB-38), U-87MG glioblastoma (ATCC, HTB-14), and MNK-45 gastric cancer (a gift from Dr. M. Broggini, Mario Negri Institute) were cultured in RPMI medium (Euroclone, Milan, IT) supplemented with 10% inactivated fetal bovine serum (HyClone, Thermo Scientific, South Logan, UT), 1% l-glutamine (Euroclone), and 100 μg/mL penicillin/streptomycin (Euroclone). HEK293T and HeLa cells were cultured in Dulbecco’s Modified Eagle Medium (DMEM, Gibco) supplemented with 10% (v/v) fetal bovine serum (FBS, Sigma) and 100 international units/mL (IU/mL) penicillin and 100 μg/mL streptomycin (Gibco). The cells were routinely tested for mycoplasma contamination and STR-authenticated.

Twenty-four hours following seeding, exponentially growing cells were treated with the different compounds at concentrations ranging from 10 to 100 μM for 72–120 h. Aqueous DMSO (0.5%, v/v) was used as a control (untreated). Cell viability was determined by the 3-(4,5-dimethylthiazol-2-yl)-2,5-diphenyltetrazolium bromide (MTT) colorimetric assay (Sigma-Aldrich). In summary, exponentially growing cancer cells were seeded in sextuplicate in 96-well culture plates (1.5 × 10^3^ cells/well). After 24 h, the compounds were added at concentrations ranging from 10 to 100 μM. The effect of compounds on cancer cell proliferation was assessed by measuring the MTT dye absorbance of the cells as reported.^52^ For combination treatments, the cells were treated with each of three compounds, either alone or in combination with doxorubicin. In particular, U-87MG cells were treated with doxorubicin alone or in combination with different MINA53 inhibitors for 24 h. The effect of different compounds or combinations (drug concentration ratio 1:20, doxorubicin: compound) on U-87MG cancer cell viability was assessed by measuring the MTT dye absorbance of cells. The interaction between doxorubicin (Doxo) and different compounds was evaluated based on the combination index (CI) using the CI equation through CalcuSyn software (Biosoft, Cambridge, U.K.). Synergism CI < 0.9, additivity 0.9 < CI < 1.1, antagonism CI > 1.1.

### Immunofluorescence (IF) Analysis and Automated Quantification

For immunofluorescence (IF) analysis, 50 × 10^4^ U-87MG cells were grown on sterile coverslips. Twenty-four hours after plating, the cells were treated with the different compounds (IOX-1 at 100 μM, GSK-J4 at 10 μM, CPI-455 at 25 μM, and both MINA53 inhibitors **9** and **10** at 50 μM) for 48 h. IF staining for different trimethylated lysine residues of histone H3 (H3K27me3, H3K9me3, H3K27me3) were carried out on PFA-fixed cells blocked with 20% goat serum in PBS (1 h, RT) and incubated overnight at 4 °C with primary rabbit polyclonal antibodies against H3K4me3 (Cell Signaling, C42D8, 1:400), H3K9me3 (Cell Signaling, D4W1U, 1:400), and H3K27me3 (Cell Signaling, C36B11, 1:400). Primary antibodies were detected using rabbit anti-IgG secondary antibodies conjugated with either Cy3 (Jackson Immunoresearch 711-165-152). DNA was stained using 0.05 μg/mL 4′,6-diamidino-2-phenylindole (DAPI), and after washing, the coverslips were mounted in Vectashield mounting media. The samples were acquired using a Nikon Eclipse 90i microscope equipped with air 40× and 100× (oil immersion; N.A. 1.3) objective and a Qicam Fast 1394 CCD camera (QImaging). Serial Z stacks of 0.4 μm thickness were acquired, taking care to cover the entire cell volume.

For quantification of fluorescence intensity signals, images of untreated or treated cells were analyzed and processed using open-source Cell Profiler 4.1.3 image analysis software (https://cellprofiler.org/) to measure fluorescence-integrated intensity values for antibody-binding relating to individual cells. A size threshold ranging from 40 to 100 pixels to exclude debris and staining noise was used. This setting identified nuclear DNA staining as a primary object. The antibody-binding relating to individual cells (secondary object) was identified on the primary input object using the propagation method.

### Western Blot and Flow Cytometric Analyses

Cell cycle distribution and apoptosis were assayed by staining floating and adherent cells with propidium iodide (50 mg/mL RNase A and 20 μg/mL propidium iodide) for 45 min. Flow cytometric analyses were performed using Epics XL apparatus (Beckman Coulter). Ten thousand events were collected from each sample, and data were processed using Flowing software.

For Western Blot experiments, the cells were lysed in JIES (100 mM NaCl, 20 mM Tris-HCl [pH 7.4], 5 mM MgCl_2_, 0.5% (v/v) NP-40) supplemented with 1× SIGMAFAST protease inhibitor cocktail (Sigma-Aldrich, S8830), or RIPA buffer; 40 μg of total protein was resolved in 4–12% gradient gels by SDS-PAGE. Proteins were transferred onto nitrocellulose or PVDF membranes and incubated with rabbit γ-H2AX (#9718, Cell Signaling Technologies, MA) anti-GAPDH (Sc47724, Santa Cruz Biotechnology, Santa Cruz, CA), rabbit anti-RPL27A (Abcam, ab74731), goat anti-RPL8 (Abcam, ab63941), horseradish peroxidase (HRP)-conjugated anti-HA (Roche, 12013819001), and HRP-conjugated anti-β-actin (Abcam, ab-49900). The membranes were subsequently probed with the appropriate horseradish peroxidase-conjugated secondary antibodies (Santa Cruz Biotechnology), and antigens on the membrane were revealed by enhanced chemiluminescence (RPN2209, GE Healthcare, Little Chalfont, U.K.).

### Immunoprecipitation

For immunoprecipitation, the cells were harvested on ice and lysed by rotation for 1 h at 4 °C in JIES buffer. The cell debris was subsequently pelleted by centrifugation (10 min at 21 910*g* at 4 °C), and the supernatant was incubated overnight at 4 °C with Pierce anti-HA agarose beads (Thermo Scientific, 26181). The next day, the beads were washed 6 times with JIES buffer before the immunoprecipitates were eluted for 15 min at 1400 rpm at room temperature using 1 mg/mL HA peptide (Thermo Scientific, 26184) dissolved in JIES buffer. The eluted samples were then prepared in 1× Laemmli loading buffer (6x: 125 mM Tris-HCl [pH 6.8], 6% (w/v) SDS, 50% (v/v) glycerol, 225 mM dithiothreitol (DTT), and 0.1% (w/v) bromophenol blue) and boiled for 5 min for detection by western blot.

### Plasmids

The coding sequences of MINA53 and NO66 were polymerase chain reaction (PCR) amplified, driven by the Phusion High-Fidelity DNA polymerase (New England Biolabs (NEB), M0530). PCR primers were designed to contain an N-terminal hemagglutinin (HA)-epitope tag and restriction sites for subsequent ligation into the pEF6 mammalian expression vector. Cloned constructs were confirmed by sequencing.

### Transfection

Plasmid DNA transfections were performed using a FuGENE 6 transfection reagent (Promega, E2691). Briefly, a transfection mix of DNA and transfection reagent at a ratio of 1:3 was made up in Opti-MEM (Gibco, 31985070) and incubated for 30 min at room temperature before the complexes were added to the cells. A total of 500 ng of DNA was used per mL of culturing medium. The cells were seeded 24 h prior to transfection, and cell treatments were performed 24 h post-transfection.

## Supporting Information Available

Elemental analyses, melting points, and yield data for compounds **2**–**13**; inhibitory activity data of compounds **1**–**13** against KAT enzymes; evaluation of the kinetic parameters and validation of the MS-based hydroxylation assays for MINA53 and NO66; AlphaScreen assay conditions; data collection and refinement statistics for the **8**-KDM5B complex structure; all molecular modeling details; effects of **1**, **9**, and **10** on cell viability, substrate trapping, and H2AX phosphorylation; and HPLC traces for compounds **7**–**10**. (PDF)
Molecular formula strings for the synthesized compounds (CSV)

## Accession Codes

The atomic coordinates and the experimental data for the KDM5B complex with compound **8** have been deposited to the PDB with accession code 5FZI and are publicly available.

## Abbreviations

AA: anacardic acid
ALCL: anaplastic large cell lymphoma
AML: acute myeloid leukemia
APL: acute promyelocytic leukemia
BD: best docked
CI: combination index
FAS: ferrous ammonium sulfate
FIH: factor inhibiting HIF
HIF: hypoxia-inducible factor
JmjC: Jumonji-C
KAT: lysine acetyltransferase
KDMs: lysine demethylases
l-AA: l-ascorbic acid
LR: lowest RMSD
Mdig: mineral dust-induced gene
MINA: MYC-induced nuclear antigen
MTT: 3-(4,5-dimethylthiazol-2-yl)-2,5-diphenyltetrazolium bromide
NOG: *N*-oxalylglycine
NO66: nucleolar protein 66
2OG: 2-oxoglutaric acid
2,4-PDCA: pyridine-2,4-dicarboxylate
PHD: prolyl hydroxylase domain
RIOX: ribosomal oxygenase
SGC: Structural Genomic Consortium
SPE: solid-phase extraction
