# Supplementary material for: First-in-Class Inhibitors of the Ribosomal Oxygenase MINA53
Source: J Med Chem. 2021 Nov 29;64(23):17031–50. doi: 10.1021/acs.jmedchem.1c00605 (PMC8667043; doi:10.1021/acs.jmedchem.1c00605)
Supplement: Supplementary file 1 — jm1c00605_si_001.pdf [file jm1c00605_si_001.pdf]

SUPPORTING INFORMATION

First-in-Class Inhibitors of the Ribosomal Oxygenase MINA53

Radosław P. Nowak,[†,‡] *Anthony Tumber,*[†,¥] *Eline Hendrix,*[≠] *Mohammad Salik Zeya Ansari,*[¶] *Manuela Sabatino,*[€] *Lorenzo Antonini,*[€] *Regina Andrijes,*[≠] *Eidarus Salah,*[¥] *Nicola Mautone,*[§] *Francesca Romana Pellegrini,*[¶¶] *Klemensas Simelis,*[¥] *Akane Kawamura,*[ç] *Catrine Johansson,*[†,¥] *Daniela Passeri,*[°] *Roberto Pellicciari,*[°] *Alessia Ciogli,*[§] *Donatella Del Bufalo,*[#] *Rino Ragno,*[€] *Mathew L. Coleman,**[*,≠] *Daniela Trisciuoglio,*[¶] *Antonello Mai,**[*,§] *Udo Oppermann,**[*,†] *Christopher J. Schofield,**[*,¥] *and Dante Rotili**[*,§]

[†] Botnar Research Centre, Nuffield Orthopaedic Centre, University of Oxford, Headington, OX3 7LD, UK.

[¥] Chemistry Research Laboratory, Department of Chemistry and the Ineos Oxford Institute for Antimicrobial Research, 12, Mansfield Road, University of Oxford, Oxford, UK, OX1 3TA, UK.

[≠] Institute of Cancer and Genomic Sciences, University of Birmingham, Edgbaston, Birmingham B15 2TT, UK.

[¶] Institute of Molecular Biology and Pathology (IMBP), National Research Council (CNR) c/o Department of Biology and Biotechnology "Charles Darwin" Sapienza University of Rome, Via degli Apuli 4, Rome 00185, Italy.

[€] Rome Center for Molecular Design, Department of Chemistry and Technology of Drugs, "Sapienza" University of Rome, Piazzale Aldo Moro 5, Rome 00185, Italy.

[§] Department of Chemistry and Technology of Drugs, "Sapienza" University of Rome, Piazzale Aldo Moro 5, Rome 00185, Italy.

[ç] Chemistry - School of Natural and Environmental Sciences, Newcastle University, Newcastle upon Tyne, NE1 7RU, UK.

[°] TES Pharma S.r.l. Via P. Togliatti 20, 06073 Corciano Perugia, Italy.

[#] Preclinical Models and New Therapeutic Agents Unit, IRCCS-Regina Elena National Cancer Institute, Via Elio Chianesi 53, Rome 00144, Italy.

*For C.J.S.: phone, +44 (0) 1865 275625; E-mail: christopher.schofield@chem.ox.ac.uk

*For D.R.: phone, +39-0649913237; E-mail: dante.rotili@uniroma1.it

*For U.O.: phone, +44 (0)1865 227308; E-mail: udo.oppermann@sgc.ox.ac.uk

*For A.M.: phone, +39 06 49913392; fax, +39 06 49693268; E-mail, antonello.mai@uniroma1.it.

*For M.C.: phone, +44 1214143943; E-mail, m.coleman@bham.ac.uk

**Contents:**	**Page(s):**
**Table S1.** Inhibitory activity of **1-13** at 100 μM against p300 and PCAF histone lysine acetyltransferase (KAT) enzymes.	p. S5
**KAT Inhibition Assays**	p. S6
**Table S2.** Melting point and yield data for compounds **2-13**.	p. S7
**Table S3.** Elemental analyses for compounds **2-13**.	p. S7
**Figure S1.** Enzyme progress curve, monitoring appearance of hydroxylated RPL8-His216OH and RPL27A-His39OH peptides for NO66 and MINA53, respectively.	p. S8
**Figure S2.** DMSO sensitivity of the MS hydroxylation assays for MINA53 and NO66.	p. S9
**Figure S3.** Determination of the K_M constant for Fe(II) with MINA53 (Met26-Val464) and NO66 (Ser183-Asn641).	p. S10
**Table S4.** Final optimized conditions for the MS MINA53 and NO66 assays.	p. S11
**Figure S4.** Optimization of MS hydroxylation assay conditions for NO66.	p. S12
**Figure S5.** Optimization of MS hydroxylation assay conditions for MINA53.	p. S13
**Figure S6.** Temperature-dependent activities of MINA53 (Met1-Val464) and NO66 (Ala167-Asn641).	p. S14
**Figure S7.** Dose response inhibition of hydroxylation activity with 2OG inhibitors in MINA53 and NO66 MS hydroxylation assays.	p. S15
**Table S5.** AlphaScreen assay conditions.	p. S16
**Figure S8.** Counter-screen of 2-(aryl)alkylthio-3,4-dihydro-4-oxopyrimidine-5-carboxylic acids for AlphaScreen signal interference.	p. S17
**Figure S9.** Overall fold of KDM5B complexed with **8**.	p. S17

**Figure S10.** Alignment between MINA53 bound RPL27A (PDB: 4BXF) and of KDM5B in complex with **8**.	p. S18
**Table S6.** Data collection and refinement statistics for the **8**-KDM5B complex structure.	p. S19
**Figure S11.** Tautomeric forms of the compounds **8** and **10**.	p. S20
**Figure S12.** Three-dimensional structures of (A) $\mathbf{8}_{Tauto-1}$, (B) $\mathbf{8}_{Tauto-2}$, (C) $\mathbf{8}_{Tauto-3}$, (D) $\mathbf{10}_{Tauto-1}$, (E) $\mathbf{10}_{Tauto-2}$ and (F) $\mathbf{10}_{Tauto-3}$ optimized at the M06/6-311++G** level of theory.	p. S21
**Table S7.** Docking assessment results obtained from random conformation cross-docking.	p. S22
**Table S8.** Total docking score obtained from the docking of the three tautomers with MINA53 and NO66.	p. S23
**Figure S13.** Best docked conformations for compound **8** on MINA53 (A) and NO66 (B), and compound **10** on MINA53 (C) and NO66 (D).	p. S24
**MD Investigations on the 8/KDM5B System**	p. S25
**Figure S14.** A) Ligand RMSD calculated along the trajectories; B) ligand RMSD KDE analysis.	p. S26
**Table S9.** MM/GBSA calculated binding free energy for compound **8** tautomers on KDM5B.	p. S26
**Figure S15.** MD C1 analysis of ligand RMSD with respect to pose C (x axis) and pose A (y axis). Points are colored according to calculated KDE density.	p. S27
**Table S10.** MM/GBSA calculated binding free energies for selected poses of compound **8** with MINA53 and NO66.	p. S28
**Table S11.** MM/GBSA calculated binding free energies for selected poses of compound **10** with MINA53 and NO66.	p. S29
**Figure S16.** Protein backbone and ligand RMSD for $\mathbf{8}_{Tauto-1}$/MINA53, $\mathbf{8}_{Tauto-3}$/NO66, $\mathbf{10}_{Tauto-1}$/MINA53, and $\mathbf{10}_{Tauto-1}$/NO66 simulations.	p. S30
**Computational Methods**	p. S31-32
**Table S12.** Inhibitory activity of **10′** against MINA53 and NO66.	p. S33
**Figure S17.** Effects of MINA53 inhibitors on HeLa and HEK293T cell viability.	p. S34
**Figure S18.** MINA53 substrate trapping with inhibitors **9** and **10** in cells.	p. S35
**Figure S19.** Western blot analysis and relative densitometric analysis of the phosphorylated form of H2AX (ser139) on U-87MG glioma cells exposed for	p. S36

the indicated times to **1** (negative control) or **9** and **10** (MINA53 inhibitors) at 10 μM.	
**Purity control by HPLC for compounds 7-10**	p. S37
**Figure S20.** HPLC traces for compound **7**.	p. S38
**Figure S21.** HPLC traces for compound **8**.	p. S39
**Figure S22.** HPLC traces for compound **9**.	p. S40
**Figure S23.** HPLC traces for compound **10**.	p. S41
**References**	p. S42

Table S1. Inhibitory activities of **1-13** (100μM) against the p300 and PCAF histone lysine acetyltransferases (KATs).[a]

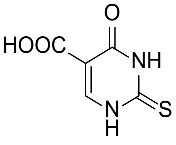

1

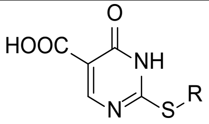

2-13

**Compd.**	**R**	**Residual Enzymatic Activity (%)**	
		**p300**	**PCAF**
**1**		98.7 ± 3.0	99.2 ± 2.7
**2**	Me	97.8 ± 3.0	100.2 ± 2.5
**3**	*n*-Bu	102.7 ± 2.0	101.3 ± 3.7
**4**	*n*-pentyl	97.7 ± 3.0	100.2 ± 2.9
**5**	*n*-nonyl	105.7 ± 3.5	104.2 ± 3.7
**6**	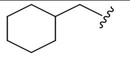	103.7 ± 4.1	99.2 ± 2.1
**7**	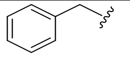	106.7 ± 3.8	101.2 ± 2.9
**8**	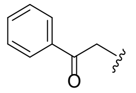	105.7 ± 3.5	103.2 ± 3.7
**9**	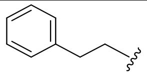	106.7 ± 4.8	102.0 ± 2.1
**10**	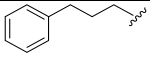	97.9 ± 2.0	101.2 ± 2.7
**11**	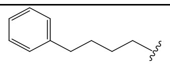	98.6 ± 2.2	100.1 ± 3.7
**12**	-CH=CHCOOEt	103.5 ± 4.1	102.6 ± 2.3
**13**	-CH=CHCOOH	107.3± 4.2	104.2 ± 4.1
**Anacardic Acid (AA)**		0.9 ± 0.01	4.7 ± 0.3

[a]Values are means ± SD of three separate experiments.

KAT Inhibition Assays[1]

The effects of **1-13** on the histone lysine acetyltransferase activity (KAT) of p300 and PCAF were determined using the HotSpot KAT assay (Reaction Biology Corporation, Malvern, PA, USA) according to the supplier's procedure. In brief, recombinant forms of catalytic domains of PCAF (aa 492-658) or p300 (aa 1284-1673) were incubated with histone H3 (5 μM) and [^{3}H]-acetyl-coenzyme A (3.08 μM, PerkinElmer) in the reaction buffer (50mMTriseHCl (pH 8.0), 50 mM NaCl, 0.1 mM EDTA, 1mM DTT, 1 mM PMSF, 1% (v/v) DMSO) for 1 h at 30 ºC in the presence or absence of various concentrations of the inhibitors. Histone H3 acetylation was assessed by liquid scintillation counting using a Tri-Carb 2800TR Liquid Scintillation Analyzer (PerkinElmer). Assays at fixed concentration doses were performed in triplicate and analyzed using Excel and GraphPad Prism 6.0 software (GraphPad Software Inc., San Diego, CA).

Table S2. Melting Point and Yield Data for Compounds **2-13**.

Compd	R	Mp (°C)	Recryst. system[a]	% Yield
**2**	-Me	228-231	A	78
**3**	*n*-Bu	106-108	B	65
**4**	*n*-pentyl	84-86	C	73
**5**	*n*-nonyl	90-92	C	53
**6**	-CH_2-*c*-hexyl	190-192	A	45
**7**	-CH_2Ph	196-198	D	71
**8**	-CH_2COPh	189-191	A	63
**9**	-$(CH_2)_2Ph$	156-158	E	65
**10**	-$(CH_2)_3Ph$	150-152	B	69
**10′**	-$(CH_2)_3Ph$	126-128	C	75
**11**	-$(CH_2)_4Ph$	146-148	B	49
**12**	-CH=CHCOOEt	153-155	D	79
**13**	-CH=CHCOOH	170-172	F	94

[a]A: methanol; B: toluene; C: cyclohexane/toluene; D: toluene/acetonitrile; E: acetonitrile; F: acetonitrile/methanol.

Table S3. Elemental Analysis for Compounds **2-13**.

Compd	Formula	Calculated, %				Found, %			
		C	H	N	S	C	H	N	S
**2**	$C_6H_6N_2O_3S$	38.71	3.25	15.05	17.22	38.86	3.27	15.00	17.18
**3**	$C_9H_{12}N_2O_3S$	47.36	5.30	12.27	14.05	47.50	5.32	12.20	14.00
**4**	$C_{10}H_{14}N_2O_3S$	49.57	5.82	11.56	13.23	49.71	5.84	11.48	13.18
**5**	$C_{14}H_{22}N_2O_3S$	56.35	7.43	9.39	10.74	56.51	7.45	9.34	10.68
**6**	$C_{12}H_{16}N_2O_3S$	53.71	6.01	10.44	11.95	53.86	6.03	10.37	11.89
**7**	$C_{12}H_{10}N_2O_3S$	54.95	3.84	10.68	12.22	55.11	3.85	10.59	12.17
**8**	$C_{13}H_{10}N_2O_4S$	53.79	3.47	9.65	11.04	53.94	3.49	9.58	10.96
**9**	$C_{13}H_{12}N_2O_3S$	56.51	4.38	10.14	11.60	56.65	4.40	10.08	11.55
**10**	$C_{14}H_{14}N_2O_3S$	57.92	4.86	9.65	11.04	58.08	4.88	9.58	10.96
**10′**	$C_{15}H_{16}N_2O_3S$	59.19	5.30	9.20	10.53	59.37	5.31	9.13	10.44
**11**	$C_{15}H_{16}N_2O_3S$	59.19	5.30	9.20	10.53	59.34	5.32	9.14	10.46
**12**	$C_{10}H_{10}N_2O_5S$	44.44	3.73	10.37	11.86	44.60	3.75	10.29	11.78
**13**	$C_8H_6N_2O_5S$	39.67	2.50	11.57	13.24	39.83	2.51	11.48	13.17

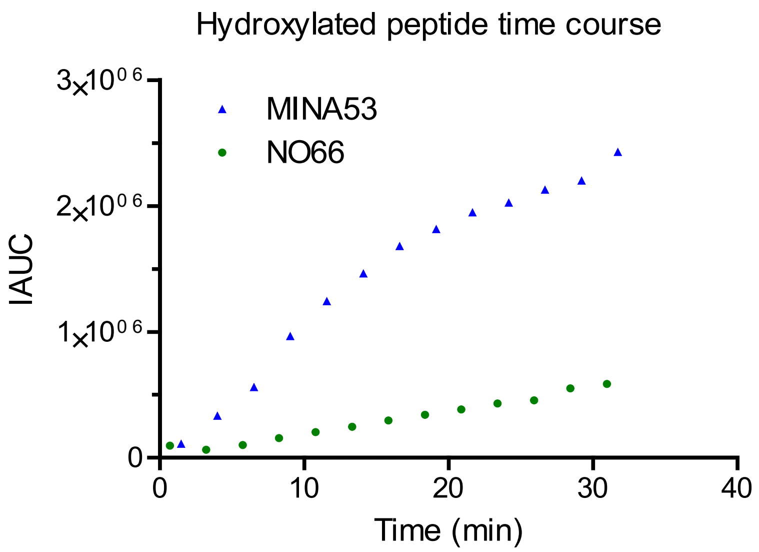

Figure S1. Progress curve, monitoring appearance of hydroxylated (*i.e.* a +16Da mass increment) RPL8-His216OH and RPL27A-His39OH peptides for NO66 (Gln116-Asn641) and MINA53 (Met1-Val464), respectively. Reactions were stopped by injection onto a RapidFire C4 cartridge. Reactions were conducted using final conditions specified in Table S4, n = 1. The MS signal represents an integrated extracted ion chromatogram (EIC) of the peptide based on the dominant charge state.

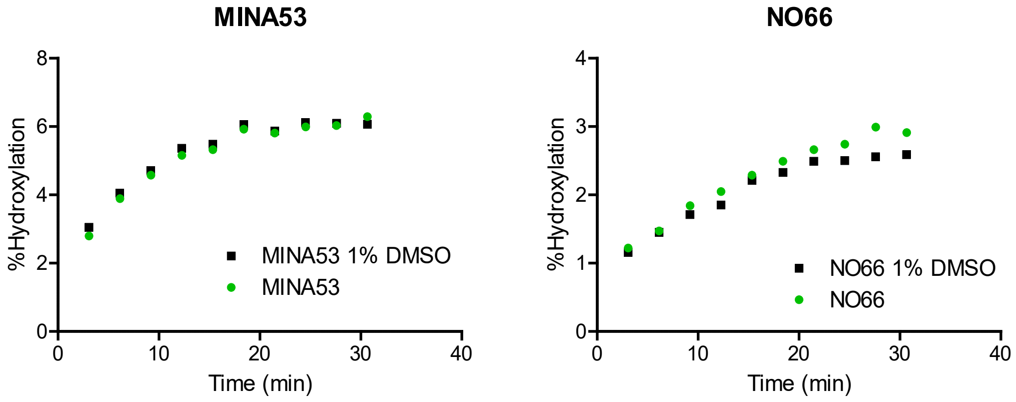

Figure S2. DMSO sensitivity of the RapidFire hydroxylation assays for MINA53 and NO66. Enzyme activity of MINA53 (M1-V464) or NO66 (Gln116-Asn641) is not inhibited by addition of 1% (v/v) aqueous DMSO, n = 1. NO66 (Gln116-Asn641) appears to be more sensitive to DMSO than MINA53, but tolerates 1% (v/v) DMSO well. Note that the data presented here came from an initial purification of both enzymes; subsequent optimized purifications led to more active material.

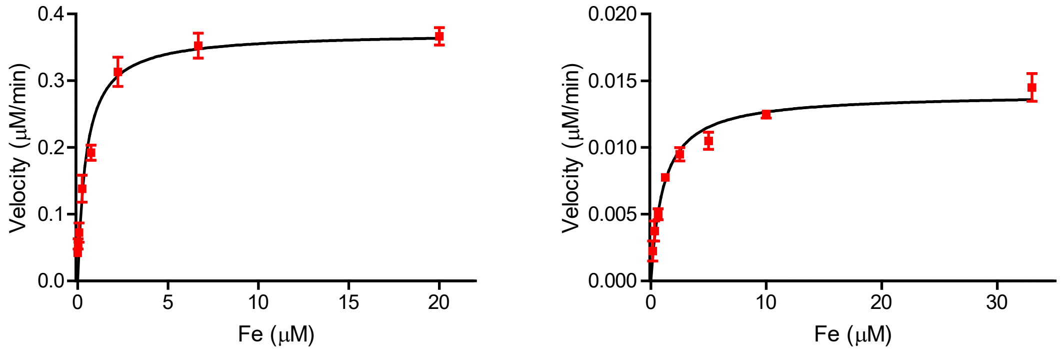

MINA53 Fe(II) K_M

K_M = 0.5 ± 0.2 μM

V_{max} = 0.37 ± 0.03 μM/min

NO66 Fe(II) K_M

K_M = 1.1 ± 0.3 μM

V_{max} = 0.014 ± 0.001 μM/min

Figure S3. Determination of K_M values for Fe(II) with MINA53 (Met26-Val464) and NO66 (Ser183-Asn641). See Table S4 for assay details. Values are means ± 95% confidence, n = 4.

Table S4. Final conditions for the RapidFire MINA53 and NO66 assays optimized for enzyme, substrate, 2OG, Fe(II) (FAS, ferrous ammonium sulphate), and *L*-AA (*L*-ascorbic acid) concentrations, buffer, pH, and temperature.[a]

	Final Assay Conditions
**Enzyme**	0.150 μM MINA53<br>0.300 μM NO66
**Substrate**	5 μM
**2OG**	2 μM
**Fe(II) (FAS)**	50 μM
***L*-ascorbic acid (LAA)**	100 μM
**Buffer**	50 mM MES (NO66), 50 mM HEPES + 50 mM NaCl (MINA53)
**pH**	7.0 (NO66), 7.5 (MINA53)
**Temperature**	Room Temperature

[a]See methods for detailed assay procedures.

Buffer	Salt	Concentration	pH 5.5	pH 6	pH 6.5	pH 7	pH 7.5	pH 8	pH 8.5	pH 9
MES	NaCl	0 mM	3%	11%	15%	30%				
		50 mM	2%	4%	12%	25%				
		100 mM	1%	4%	8%	24%				
		150 mM	1%	3%	6%	24%				
	KCl	50 mM	2%	4%	9%	24%				
		100 mM	1%	3%	9%	26%				
HEPES	NaCl	0 mM				27%	15%	14%		
		50 mM				18%	12%	12%		
		100 mM				14%	12%	11%		
		150 mM				13%	13%	10%		
Tris	NaCl	0 mM					3%	2%	2%	1%
		50 mM					4%	2%	1%	1%
		100 mM					5%	2%	5%	1%
		150 mM					6%	1%	1%	1%
	KCl	50 mM					5%	1%	1%	1%
		100 mM					5%	1%	1%	1%
BIS-TRIS	NaCl	0 mM		6%	6%	3%	1%			
		50 mM		6%	6%	3%	2%			
		100 mM		5%	5%	4%	2%			
		150 mM		4%	5%	4%	2%			
	KCl	50 mM		4%	7%	3%	1%			
		100 mM		4%	6%	3%	1%			

pH	5.4	5.6	5.8	6	6.2	6.4	6.6	6.8	7	7.2	7.4	7.5
Phosphate	1%	2%	3%	4%	4%	3%	3%	3%	4%	2%	2%	2%

Figure S4. Optimization of MS hydroxylation assay conditions for NO66. Percentage substrate turnover for NO66 (Ala167-Asn641) after 60 min incubation with 50 mM MES, HEPES, Tris, Bis-Tris or phosphate buffers with a range of NaCl or KCl salt concentrations. NO66 was used at a concentration of 300 nM, with 5 µM 2OG, 5 µM peptide substrate, 50 µM ferrous ammonium sulphate (FAS) and 100 µM *L*-ascorbic acid (*L*-AA), N=1.

Buffer	Salt	Concentration	pH 5.5	6	6.5	7	7.5	8	8.5	9
MES	NaCl	0 mM	3%	15%	24%	22%				
		50 mM	3%	11%	32%	27%				
		100 mM	3%	13%	26%					
		150 mM	2%	6%	17%	23%				
	KCl	50 mM	3%	11%	26%	36%				
		100 mM	2%	10%	26%	30%				
HEPES	NaCl	0 mM				36%	40%	39%		
		50 mM				39%	41%	38%		
		100 mM				38%	35%	32%		
		150 mM				31%	29%	29%		
TRIS	NaCl	0 mM					26%	24%	11%	7%
		50 mM					25%	22%	9%	5%
		100 mM					26%	21%	9%	6%
		150 mM					19%	21%	4%	2%
	KCl	50 mM					27%	21%	8%	3%
		100 mM					30%	19%	7%	2%
BIS-TRIS	NaCl	0 mM		16%	25%	21%	15%			
		50 mM		22%	36%	28%	19%			
		100 mM		16%	36%	33%	22%			
		150 mM		25%	33%	27%	16%			
	KCl	50 mM		22%	37%	27%	17%			
		100 mM		26%	38%	32%	18%			

pH	5.4	5.6	5.8	6	6.2	6.4	6.6	6.8	7	7.2	7.4	7.5
Phosphate	2%	8%	12%	15%	11%	13%	10%	10%	8%	6%	6%	5%

Figure S5. Optimization of MS hydroxylation assay conditions for MINA53. Activity (measured as peptide turnover) for MINA53 (Met1-Val464) with 50 mM MES, HEPES, Tris, Bis-Tris or phosphate buffers over a range of NaCl or KCl salt concentrations. Full-length recombinant MINA53 was used at 300 nM, with 5 µM 2OG, 5 µM substrate, 50 µM ferrous ammonium sulphate (FAS) and 100 µM *L*-ascorbic acid (*L*-AA), n = 1.

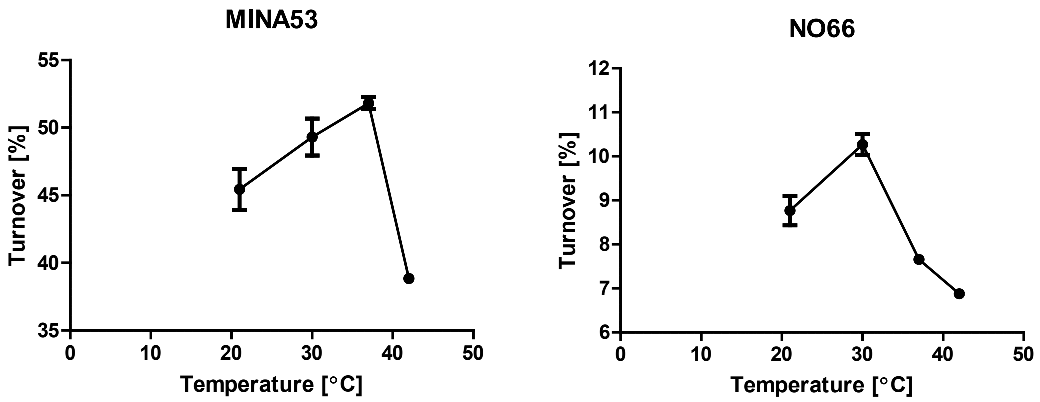

Figure S6. Temperature-dependent activities of MINA53 (Met1-Val464) and NO66 (Ala167-Asn641). Percentage turnover with 12 replicates per data point for NO66, and 8 replicates for MINA53 except for 21˚C where 5 replicates were used. Assays were performed in a water bath equilibrated to 21, 30, 37 and 42 ˚C; reactions were stopped after 30 min for MINA53 and after 60 min for NO66.

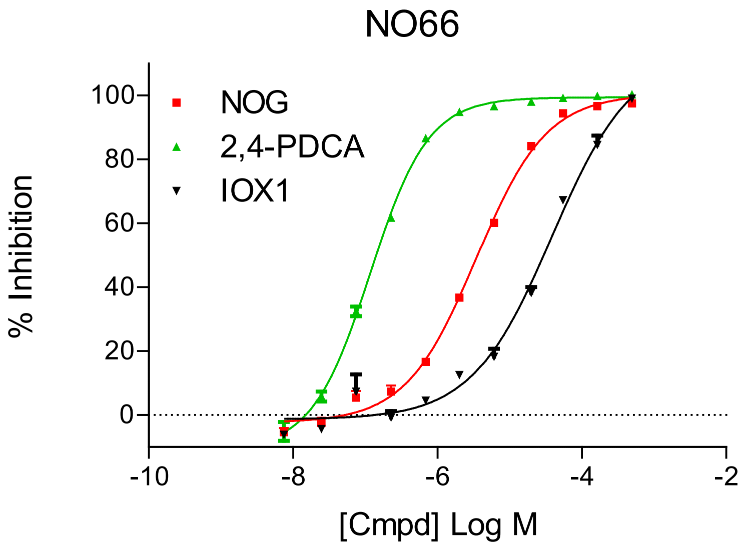

Compound	IC_{50} value
NOG	3.5 ± 0.5 μM
2-4 PDCA	0.11 ± 0.01 μM
IOX1	38.5 ± 18.0 μM
GSK-J1	30% inhibition at 100 μM

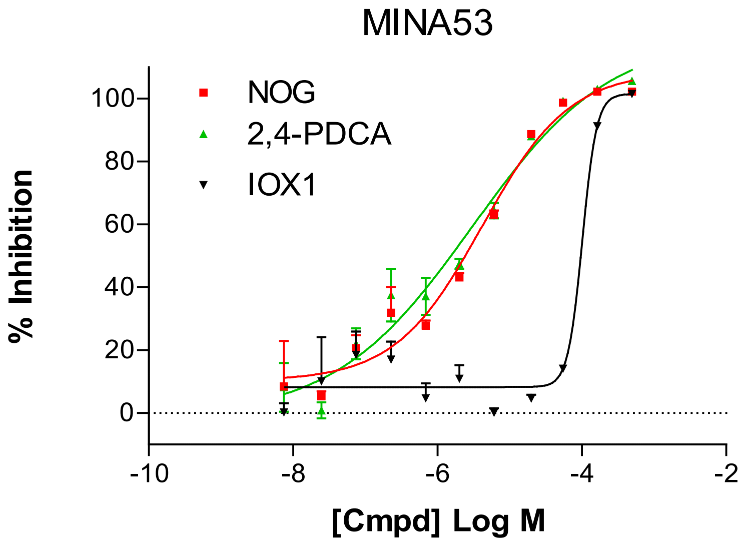

Compound	IC_{50} value
NOG	1.8 ± 0.7 μM
2,4-PDCA	1.3 ± 0.5 μM
IOX1	101.8 ± 35.1 μM
GSK-J1	No inhibition at 100 μM

Figure S7. NO66 and MINA53 hydroxylation inhibition assays. IC_{50} curves with MINA53 (Met1-Val464) and NO66 (Ser183-Asn641) for 2,4-PDCA (pyridine-2,4-dicarboxylate) and NOG (*N*-oxalylglycine), two established broad-spectrum inhibitors[2] of 2OG oxygenases, and IOX1.[3] The plot for GSK-J1 is not shown for clarity. Values are means ± 95% confidence, n = 3. Note, although these compounds are relatively broad spectrum 2OG oxygenase inhibitors, their potency against different 2OG oxygenases varies, as manifest in our data for MINA53 and NO66, where 2,4-PDCA and NOG are more potent than IOX-1, which is active against multiple JmjC KDMs.[3]

Table S5. AlphaScreen assay conditions.

**Enzyme**	**KDM2A (M1-P517) *N*-terminal His6-tagged**	**KDM5B (L882-S1761) *N*-terminal His6-tagged**	**KDM3B (L882-S1761) *N*-terminal His6-tagged**	**KDM 4A (M1-L359)**	**KDM4D (M1-G373) *N*-terminal His6-tagged**	**KDM6B (D1141-R1641)**
Expression system	Insect cells	Insect cells	Insect cells	*E. coli*	*E. coli*	*E. coli*
Assay Buffer	50 mM HEPES pH 7.5, 0.01% (v/v) Tween-20, 0.1% (w/v) BSA.<br>*80 nM Non-Biotinylated H3K36Me2 substrate was added to FBXL11 assay buffer to improve the antibody readout.					
2OG (μM)	10	5	5	10	10	10
FAS (μM)	10	10	10	1	1	10
LAA (μM)	100	100	100	100	100	100
Peptide (nM)	100	100	60	30	30	60
Protein Conc. (nM)	25	2	0.4	4	2	0.1
Peptide Description	H3K36(Me2) -21mer; biotin-SAPATGGVK(Me2)KPHRYRPGTVAL (Anaspec)	H3K4(Me3) -21mer; ARTK(3me)QTARKSTGGKAPRKQLA-GGK spacer-Biotin (Anaspec)	H3K9(Me2) -21mer; ARTKQTARK(2me)STGGKAPRKQLA-GGK spacer-Biotin (Anaspec)	H3K9(Me3) -21mer; ARTKQTAR-K(Me3)-STGGKAPRKQLA-GGK-Biotin (Anaspec)		H3K27(Me3)-21mer; Biotin-KAPRKQLATKAAR(Kme3)SAPATGG (Anaspec)
Antibody	Anti-Histone H3K36me1 antibody (Abcam AB9048)	Anti-H3K4me2 antibody (CST 9725S)	Anti H3K9me1 antibody (Abcam Ab8896)	Anti H3K9me2 antibody (Abcam Ab1220)		Anti H3K27me2 antibody (Millipore 07-452)
Solvent Conc. (%)	0.5	0.5	0.5	0.5	1	0.5
Reaction time (min)	30	20	5	20	8	10
Bead Donor (mg/ml)	0.02					
Bead Acceptor (mg/ml)	0.02					
Antibody Conc. (μg/ml)	0.0075	1 in 2500 (v/v)	0.05	0.05	0.05	0.4
Bead Incubation time (min)	120					

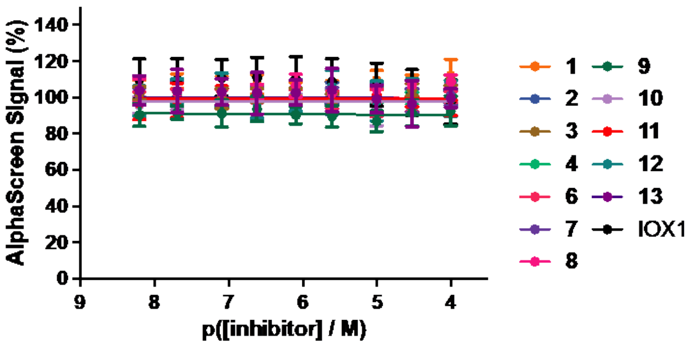

Figure S8. Counter-screen of 2-(aryl)alkylthio-3,4-dihydro-4-oxopyrimidine-5-carboxylic acids for AlphaScreen signal interference. Inhibitors were pre-incubated with the biotinylated-product and a product-specific antibody and added to a streptavidin-coated donor and Protein A-coated acceptor bead mixture. Data are normalised to a DMSO control (100%), with no biotin-substrate as the baseline; n= 3 +/- STD.

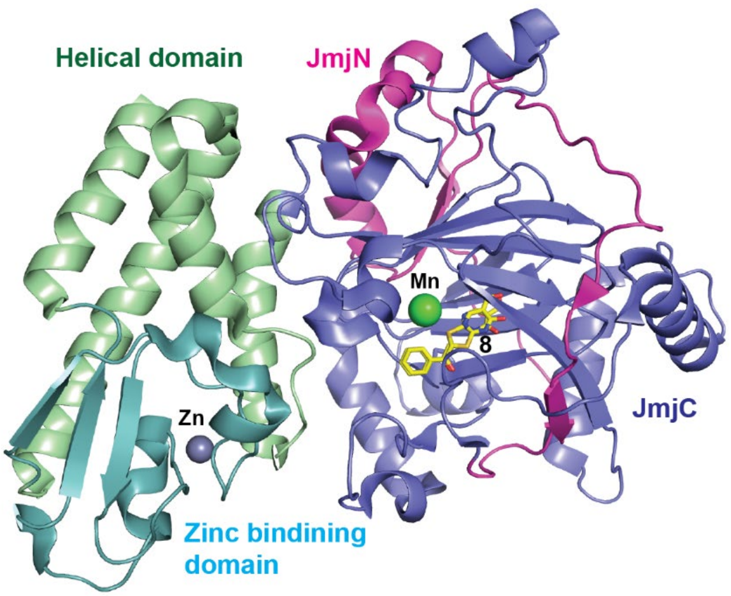

Figure S9. View from a structure of KDM5B in complex with 8 (PDB ID: 5FZI). **8** occupies the 2OG co-substrate binding pocket of the KDM5B JmjC domain.

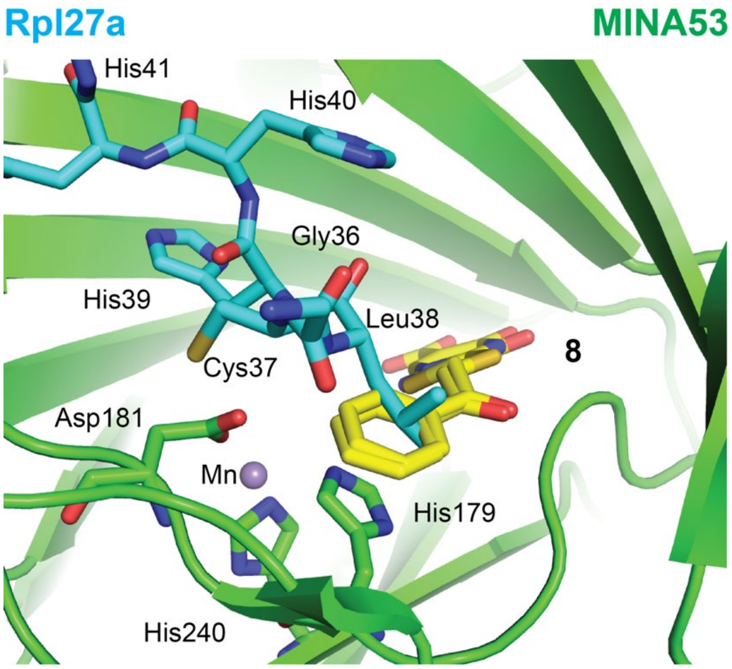

Figure S10. **Superimposition of views from structures of MINA53 in complex with RPL27A (PDB ID: 4BXF) and of KDM5B in complex with 8 (PDB ID: 5FZI).** Note that Leu38 of RPL27A is predicted to clash with the phenyl ring of **8**. The KDM5B protein is omitted from the view with only **8** shown for clarity. The overlay was performed on the catalytic metal coordinating triad (His400, Glu501 and His587 of KDM5B, and His179, Asp181 and His240 of MINA53).

**Table S6.** Data collection and refinement statistics for the **8**-KDM5B complex structure.	
	**8 – PDB 5FZI**
Wavelength (Å)	0.9763
Resolution range (Å)	95.6 - 1.95 (2.02 - 1.95)
Space group	$P6_522$
Unit cell a(Å),b(Å),c(Å) α β γ	141.97 141.97 152.03 90 90 120
Total reflections	1305566 (129987)
Unique reflections	66099 (6498)
Multiplicity	19.8 (20.0)
Completeness (%)	100.00 (100.00)
Mean I/sigma(I)	18.67 (1.72)
Wilson B-factor	38.20
R merge † (%)	0.1119 (2.216)
R-meas	0.1148
CC1/2	0.999 (0.585)
CC*	1 (0.859)
R-work‡	0.1869 (0.3030)
R-free§	0.2237 (0.3373)
Number of non-hydrogen atoms	4344
macromolecules	3791
ligands	132
water	421
Protein residues	461
RMS (bonds) ††	0.008
RMS (angles) ††	1.17
Ramachandran favoured (%)	98
Ramachandran outliers (%)	0
Clashscore	4.46
Average B-factor (^{2})	44.80
macromolecules	43.40
ligands	50.10
solvent	56.20

† $R_{\text{merge}} = \sum_{hkl} \sum_i |I_i(hkl) - \langle I(hkl) \rangle| / \sum_{hkl} \sum_i I_i(hkl)$, where $I_i(hkl)$ is the intensity of the ith measurement of reflection *hkl* and $I(hkl)$ is the mean value of $I_i(hkl)$ for all i measurements.

‡ $R_{\text{work}} = \sum_{hkl} \left| |F_{\text{obs}}| - |F_{\text{calc}}| \right| / \sum_{hkl} |F_{\text{obs}}|$, where F_{obs} is the observed structure factor and F_{calc} is the calculated structure factor.

§ R_{free} is the same as R_{cryst} except calculated with a subset (5%) of data that were excluded from the refinement calculations.

†† Engh Huber (1991).

Molecular Modeling

Compounds **8** and **10** can adopt different tautomeric forms in which the exchangeable hydrogen can be either on the pyrimidine's nitrogens (Tauto-1 and Tauto-2, Figure S11), or on the oxygen atom at the pyrimidine C-4 position (Tauto-3, Figure S11).

**R**	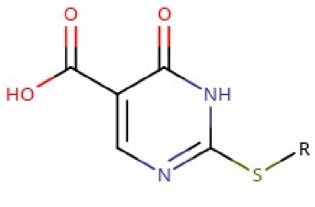	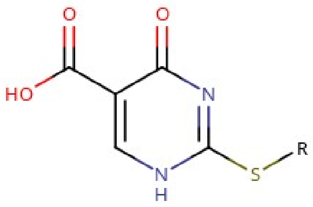	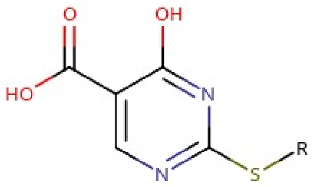
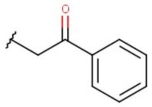	**8**$_{Tauto-1}$	**8**$_{Tauto-2}$	**8**$_{Tauto-3}$
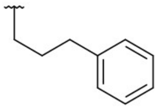	**10**$_{Tauto-1}$	**10**$_{Tauto-2}$	**10**$_{Tauto-3}$

Figure S11. Tautomeric forms of the compounds **8** and **10**.

As no experimental evidence is available on the preferred tautomeric forms, quantum mechanical calculations were done to evaluate their relative stabilities. Structure-based (SB) studies using docking simulations were applied to investigate likely ligand-protein interactions. To this, a docking assessment[4] was initially undertaken to select the best scoring function/docking algorithm in Smina[5] and Plants,[6] which are freely available. The best performing program was used to investigate the likely tautomeric forms of **8** and **10** in complex with MINA53 and NO66 using reported crystal structures. Molecular dynamics (MD) simulations were then run on the proposed docked poses in the complexes to investigate the basis of the experimentally observed activities and selectivies.

QM Calculations

QM calculations (M06/6-311++G**) on the lowest energy conformers of compound **8** tautomers indicated Tauto-1 as the favorite tautomer, followed by Tauto-3 (2.7 kcal/mol higher) and Tauto-2 (3.7 kcal/mol higher). Similarly, for compound **10**, the QM calculations indicated Tauto-1 as the

preferred tautomer, while Tauto-2 and Tauto-3 are energetically disfavored by 4.8 and 4.0 kcal/mol, respectively (Figure S12).

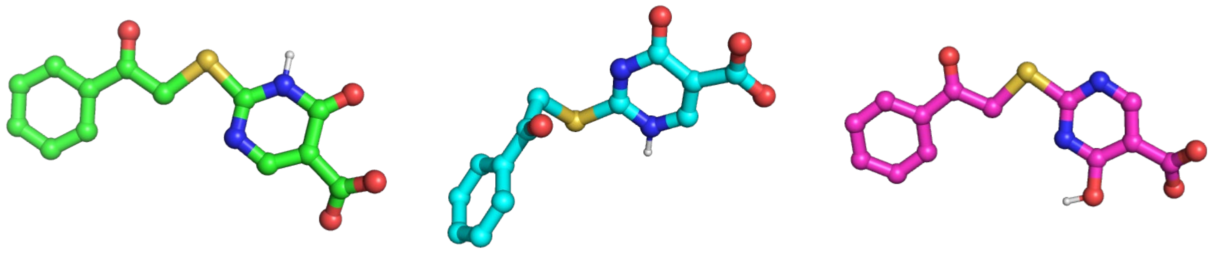

(A) **(B)** **(C)**

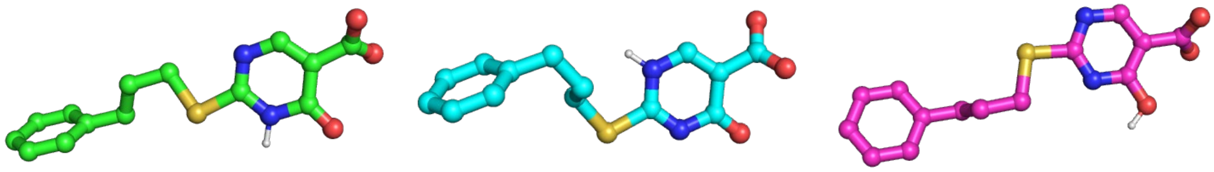

(D) **(E)** **(F)**

Figure S12. Structures of (A) **8**$_{\text{Tauto-1}}$, (B) **8**$_{\text{Tauto-2}}$, (C) **8**$_{\text{Tauto-3}}$, (D) **10**$_{\text{Tauto-1}}$, (E) **10**$_{\text{Tauto-2}}$ and (F) **10**$_{\text{Tauto-3}}$ optimized at the M06/6-311++G** level of theory.

Docking Assessment

X-ray structures of MINA53 and NO66 were used to evaluate the ability of a docking program to predict the correct geometry of protein-ligand complex (re-docking). The Plants and Smina software were investigated considering three scoring functions for each, Chemplp, Plp, Plp95 and Vina, Vinardo, Ad4 scoring, respectively. Experimental (EC) and randomized (RC) ligand conformation re-docking (RD) and cross-docking (CD) methods were used to assess the software in reproducing experimentally observed binding modes of co-crystallized inhibitors. The root mean square deviation (RMSD)[7] was calculated between the docked ligand conformation and the crystallographically observed one to investigate the docking accuracy (DA)[8,4]. Analysis of calculated docking accuracy percentage (DA%) revealed Plants with the scoring function Plp95 as the program showing the highest DA% value. (Table S7)

Table S7. Docking assessment results obtained from random conformation cross-docking.

	MINA53						NO66					
	Chemplp[a]	Plp[a]	Plp95[a]	Vina[a]	Vinardo[a]	Ad4[a]	Chemplp[a]	Plp[a]	Plp95[a]	Vina[a]	Vinardo[a]	Ad4[a]
Min[b]	1.17	1.12	1.20	0.91	0.83	1.64	1.49	1.33	1.61	1.66	1.64	2.25
Max[c]	1.65	1.39	1.59	1.45	1.20	2.67	3.65	3.95	3.64	3.64	3.62	3.56
Aver[d]	1.44	1.27	1.33	1.11	1.06	1.99	2.22	2.09	2.26	2.24	2.18	2.66
STD[e]	0.25	0.14	0.22	0.29	0.21	0.59	0.98	1.25	0.93	0.94	0.96	0.61
DA%[f]	100	100	100	100	100	83.3	62.5	75	100	75	75	37.5

[a]The scoring function names as implemented in the Plants and Smina docking programs.

[b]Minimum value of RMSD obtained for each docking software used.

[c]Maximum value of RMSD obtained for each docking software used.

[d]Average value of RMSD obtained for each docking software used.

[e]Standard deviation value.

[f]Docking accuracy as defined in the experimental section.

Molecular Docking of Compounds 8 and 10

To investigate the protein-tautomer interaction stabilities of **8** and **10**, a sort docking consensus was carried out as follows: each tautomer was docked, using Plants with the Plp95 scoring function, into MINA53 and NO66 structures available in PDB; the first five docked conformations were evaluated with a docking score (Table S8).

Although the docking score values for the three **8** and **10** tautomers docked into MINA 53 and NO66 are in a narrow range, it is possible to propose a preference of binding of $\mathbf{8}_{\text{Tauto-1}}$ for MINA53, as this combination gives the best docking score value of -117.95. For compound **10**, the best selectivity is shown by $\mathbf{10}_{\text{Tauto-1}}$ and MINA53, with a docking score of -120.75.

The best docked conformations for **8** and **10** with MINA53 were very similar (Figure S13 A and C), with both being predicted to interact via π-π stacking with Tyr167 and chelation of the Mn^{2+} ion (substituting for catalytic Fe^{2+}). By contrast with NO66, the best docked conformations were clearly different (Figure S13 B and D). However, with MINA53 the phenyl ring of **8** and with NO66 the pyrimidine ring of **10** are involved in a π-π stacking with one of the two histidine residues involved in Mn^{2+} ligation (Figure S13).

Table S8. Docking scores obtained from the docking simulation of the three tautomers of **8** and **10** with MINA53 and NO66.

	MINA53			NO66			
	2XDV	4BXF Chain A	4BXF Chain B	4CCK Chain A	4CCK Chain B	4CCK Chain C	4CCK Chain D
$\mathbf{8}_{\text{Tauto-1}}$	-99.87	-107.76	-117.95	-108.48	-110.88	-107.03	-109.08
$\mathbf{8}_{\text{Tauto-2}}$	-103.8	-108.07	-103.56	-109.40	-115.35	-107.66	-110.60
$\mathbf{8}_{\text{Tauto-3}}$	-101.59	-11.01	-104.54	-105.68	-113.83	-104.18	-106.51
$\mathbf{10}_{\text{Tauto-1}}$	-104.82	-112.91	-120.75	-115.84	-117.47	-113.40	-114.91
$\mathbf{10}_{\text{Tauto-2}}$	-107.87	-112.71	-109.44	-114.22	-116.26	-112.28	-112.62
$\mathbf{10}_{\text{Tauto-3}}$	-105.26	-110.72	-115.31	-113.05	-115.68	-111.16	-112.94

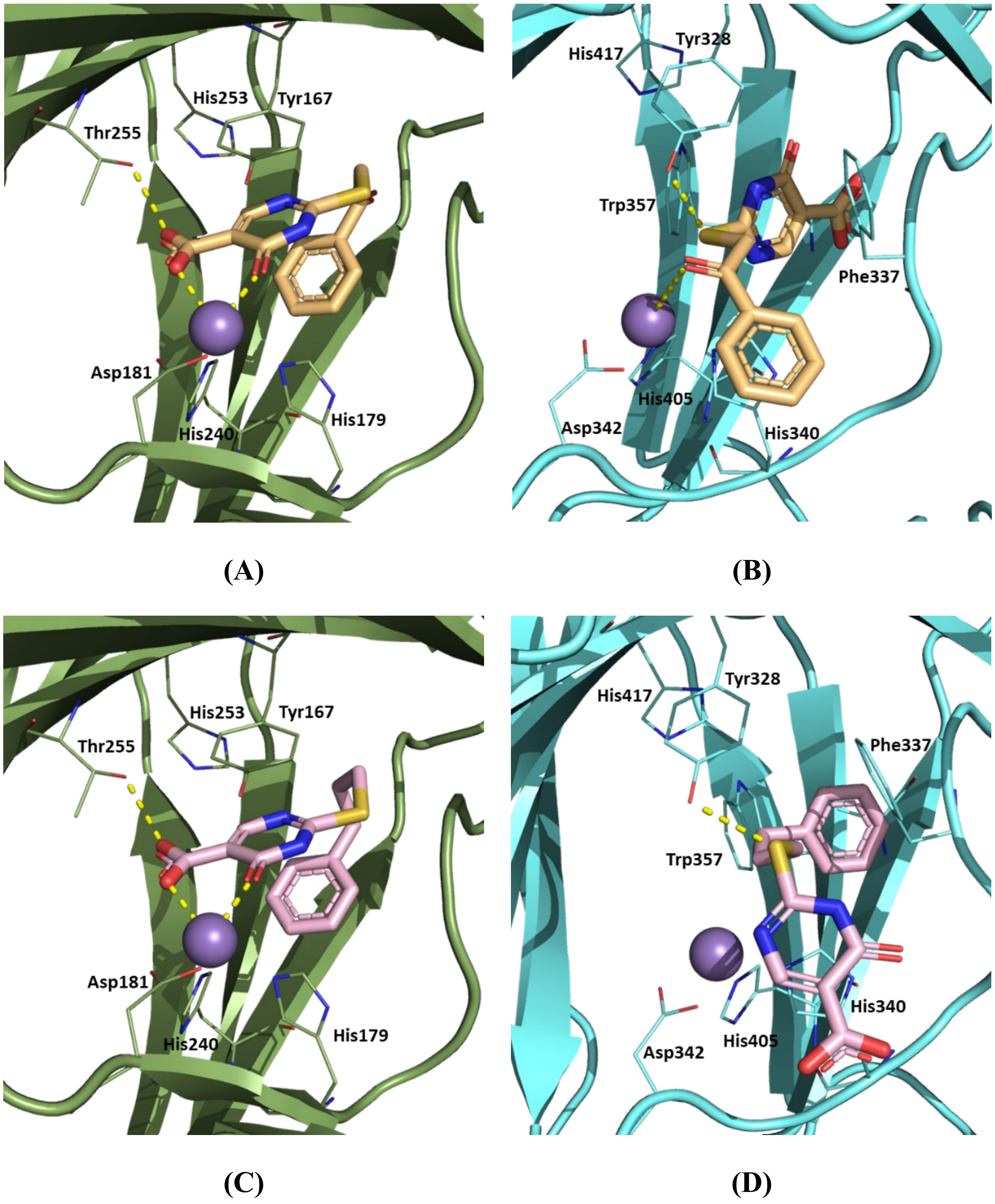

Figure S13. Best docked conformations for compound **8** with: (A) MINA53 (PDB ID: 4BXF) and (B) NO66 (PDB ID: 4CCK), and for compound **10** with (C) MINA53 (PDB ID: 4BXF) and (D) NO66 (PDB ID: 4CCK).

MD Investigations on the 8/KDM5B System

As from crystallographic data it is not possible to establish which tautomer of derivative **8** preferentially binds to KDM5B, MD simulations were performed. Starting from the reported KDM5B crystal structure (PDB ID: 5FZI) the three tautomers (**8**$_{Tauto\text{-}1}$, **8**$_{Tauto\text{-}2}$, and **8**$_{Tauto\text{-}3}$) were modeled using the experimentally observed binding mode as a template; these were labeled as conformations A and C (**8**$_{Pose\text{-}A}$ and **8**$_{Pose\text{-}C}$). A total of six different simulations (25 ns each) were performed with all combinations of the poses and tautomers: **8**$_{Pose\ A/Tauto\text{-}1}$, **8**$_{Pose\ A/Tauto\text{-}2}$, **8**$_{Pose\ A/Tauto\text{-}3}$, **8**$_{Pose\ C/Tauto\text{-}1}$, **8**$_{Pose\ C/Tauto\text{-}2}$, **8**$_{Pose\ C/Tauto\text{-}3}$. Crystallographic water molecules in the active site were retained in the MD simulations, as they are involved in establishing briding interactions between the ligand and the Mn^{+2} ion. Consistent with this, preliminary MD simulation runs without the crystallographic waters manifested large conformational changes in the inhibitor, or loss of binding during the equilibration phase. **8**$_{Pose\ A/Tauto\text{-}1}$ was the only combination showing some stability without the crystallographic waters; further in this case solvent waters were observed to move to occupy the space originally occupied by the deleted active site crystallographic waters (data not shown). Subsequent MD simulations thus were performed with the crystallographically observed water molecules.

The MD analyses of the **8** tautomers bound into KDM5B were accomplished by calculating the ligand root mean square deviation (RMSD) along the trajectories (Figure S14A). All trajectories returned an RMSD below 2 Å, confirming the stability of the complexes. A kernel density estimation (KDE) analysis on RMSD curves enabled analysis of the distribution of RMSD values (Figure S14B). The results indicate that **8**$_{Pose\ A/Tauto\text{-}1}$ is the most stable combination; this shows a narrow RMSD distribution centered at ~0.35 Å, the lowest value observed in all MD simulations. This analysis also indicates that for all **8** tautomers resolved conformation C is, in general, less stable than the A. Moreover, it should be noted that, for pose A, all the three tautomers could in principle bind KDM5B, but that **8**$_{Tauto\text{-}1}$ shows the best stability.

Trajectories were used to calculate ligand binding free energies using the molecular mechanics energies combined with the generalized Born and surface area continuum solvation (MM/GBSA) method.[9] The calculated free energies (Table S9) were in good agreement with the RMSD analysis: **8**$_{Tauto\text{-}1}$ is the **8** preferred tautomer, though with a small ΔΔG value. However, the binding free energy value calculated for **8**$_{Pose\ C/Tauto\text{-}1}$ was slightly lower than that for **8**$_{Pose\ A/Tauto\text{-}1}$. A visual inspection of the analyzed MD trajectory **8**$_{Pose\ C/Tauto\text{-}1}$ showed the ligand switching from alternative pose C to that of A during the first ns (Figure S15).

The joint interpretation of QM and MD simulation results clearly shows that **8**$_{Tauto\text{-}1}$ is the preferred tautomer and that experimental alternative pose A is the preferred for binding with KDM5B.

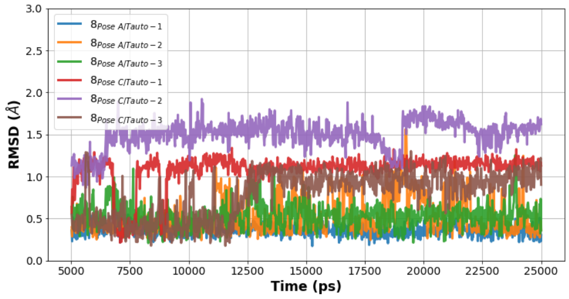

(A)

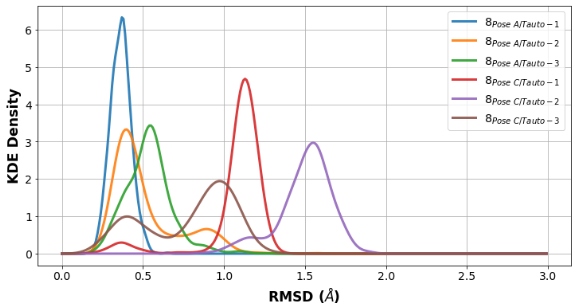

(B)

Figure S14. A) Ligand RMSD calculated along the trajectories **8**$_{\text{Pose A/Tauto-1}}$, **8**$_{\text{Pose A/Tauto-2}}$, **8**$_{\text{Pose A/Tauto-3}}$, **8**$_{\text{Pose C/Tauto-1}}$, **8**$_{\text{Pose C/Tauto-2}}$, **8**$_{\text{Pose C/Tauto-3}}$; B) ligand RMSD KDE analysis.

Table S9. MM/GBSA calculated binding free energy for compound **8** tautomers on KDM5B.

	**Tauto-1**		**Tauto-2**		**Tauto-3**	
	**ΔG (GB) (kcal/mol)**	**Std**	**ΔG (GB) (kcal/mol)**	**Std**	**ΔG (GB) (kcal/mol)**	**Std**
**Pose A**	-21.79	4.44	-21.29	1.93	-12.89	1.37
**Pose C**	-22.32	2.82	-19.07	1.84	-16.43	3.01

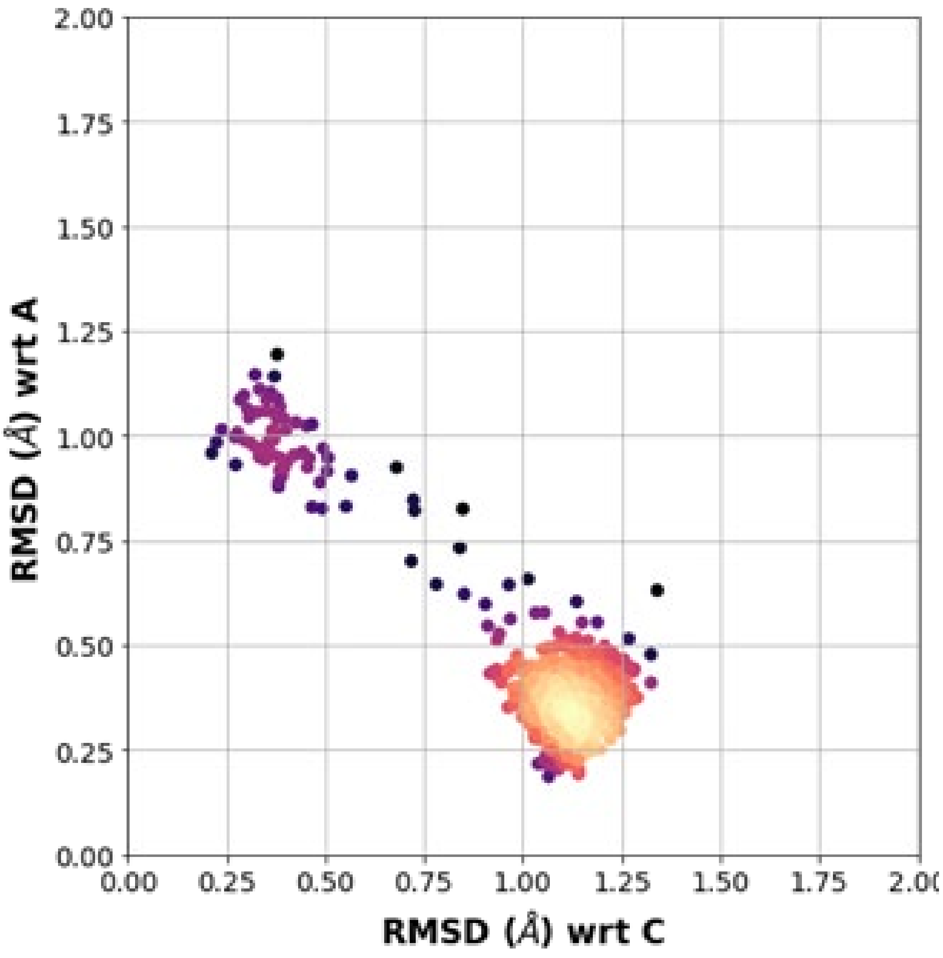

Figure S15. 8$_{\text{Pose C/Tauto-1}}$ MD analysis of ligand RMSD with respect to pose C (x axis) and pose A (y axis). Points are colored according to the calculated KDE density.

Molecular Dynamics Investigations of 8 into MINA53 and NO66

To investigate the inhibitory activity of **8** against MINA53 and NO66, molecular docking simulations coupled with MD simulations were performed as described above for KDM5B. The tautomers of **8** were docked with the MINA53 and NO66 crystal structures (see molecular docking section). Three binding poses were selected for MINA53 and NO66 for MD-based investigations: the best docked (BD, the pose characterized by the lowest score), and the poses presenting the lowest RMSD (LR) with respect to the crystalized alternative conformations (PDB ID: 5FZI) A (LRA) and C (LRC). A total of six 25 ns MD simulations were performed for **8**.

Binding poses obtained from docking were complexed with their respective locks (MINA53 key/lock systems: BD/4BXF, LRA/2XDV, LRC/2XDV; NO66 key/lock systems: BD/4CCK, LRA/4CCK, LRC/4CCK) and subjected to MD simulation. The resulting MD trajectories were analyzed and MM/GBSA binding free energies were calculated. The calculated ΔGs (Table S10) are in agreement with the observed activity profiles.

The lowest energy binding modes for **8** on MINA53 (BD, $\mathbf{8}_{\text{Tauto-1}}$) and NO66 (LRC, $\mathbf{8}_{\text{Tauto-3}}$) were visually inspected using the most representative frame of the simulation. A KDE analysis was performed on the first two components of the PCA calculated from ligand cartesian coordinates collected along the MD simulation. The frame with highest density, taken as the most representative, was then visually inspected.

Table S10. MM/GBSA calculated binding free energies for compound **8** selected poses on MINA53 and NO66.

	**MINA53**		**NO66**	
	**ΔG (GB) (kcal/mol)**	**Std**	**ΔG (GB) (kcal/mol)**	**Std**
**BD**	-136.49	4.18	-41.53	1.87
**LRA**	-88.67	5.16	-32.00	2.71
**LRC**	-88.25	8.51	-44.98	3.67

Molecular Dynamics Investigations of 10 into MINA53 and NO66

Calculations to investigate the activity of **10** versus MINA53 and NO66 used the same procedure as done for **8**. **10** was docked into the MINA53 and NO66 structures. Three poses for each protein were selected for analysis, based on best docked (BD) and lowest RMSD (LR) poses with respect to the crystallographically observed (PDB ID: 5FZI) alternative conformations A (LRA) and C (LRC) for **8**. The key/lock systems for calculations with **10** and MINA53 were: BD/4BXF, LRA/4BXF, LRC/2XDV; for calculations on NO66: BD/4CCK, LRA/4CCK, LRC/4CCK. A total of six MD simulations (25 ns each) were performed for **10**. The MM/GBSA results are consistent with experimental observations (Table S11), i.e. predict a higher affinity for **10** for MINA53 compared to NO66. The best binding mode identified by calculations for **10** with NO66 was LRA ($\mathbf{10}_{\text{Tauto-1}}$), while the best with MINA53 was BD ($\mathbf{10}_{\text{Tauto-1}}$). The BD and LRA calculated binding free energies with MINA53 had large negative ΔG values principally due to Mn^{2+} chelation.

As for **8**, for **10** the most representative frame of the simulation was extracted through KDE analysis performed on the first two components of the PCA calculated from ligand cartesian coordinates collected along the MD simulation. The frame with the highest density, taken as the most representative one, was then visually inspected.

Table S11. MM/GBSA calculated binding free energies for compound **10** selected poses on MINA53 and NO66.

	**MINA53**		**NO66**	
	**ΔG (GB)**	**Std**	**ΔG (GB)**	**Std**
**BD**	-146.70	2.42	-22.61	3.50
**LRA**	-139.03	5.06	-50.79	6.64
**LRC**	-15.72	4.32	-48.69	6.00

A RMSD analysis was conducted on both protein backbone and ligand of complexes **8**$_{\text{Tauto-1}}$/MINA53 (BD/4BXF), **8**$_{\text{Tauto-3}}$/NO66 (LRC/4CCK), **10**$_{\text{Tauto-1}}$/MINA53 (BD/4BXF), and **10**$_{\text{Tauto-1}}$/NO66 (LRA/4CCK) trajectories (Figure S16). While the backbone RMSDs displayed a comparable stability for all the investigated systems (Figure S16A and B), the ligand RMSD timeseries (Figure S16C) and KDE distributions (Figure S16D) clearly evidenced the higher stability of compounds **8**$_{\text{Tauto-1}}$ and **10**$_{\text{Tauto-1}}$ binding modes to MINA53 over those proposed for NO66, in agreement with the MM/GBSA calculations.

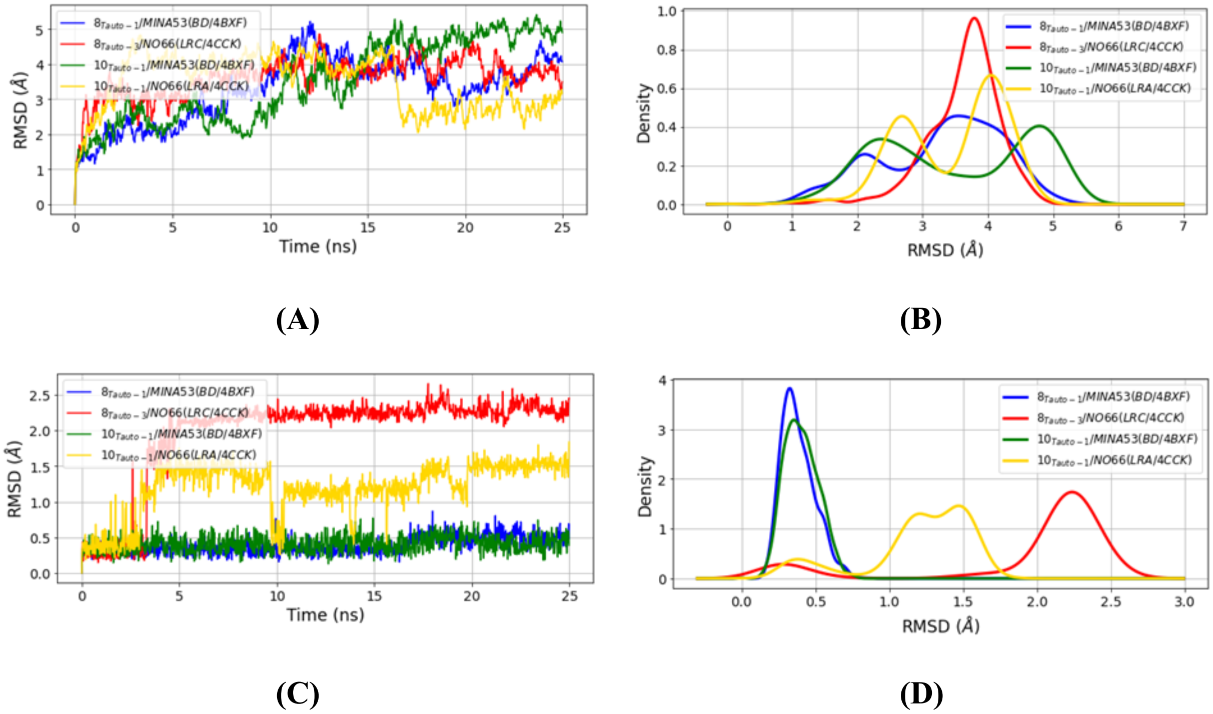

Figure S16. A) Protein backbone RMSD; B) protein backbone RMSD KDE analysis; C) ligand RMSD calculated along the trajectories of complexes **8**$_{\text{Tauto-1}}$/MINA53 (BD/4BXF), **8**$_{\text{Tauto-3}}$/NO66 (LRC/4CCK), **10**$_{\text{Tauto-1}}$/MINA53 (BD/4BXF), and **10**$_{\text{Tauto-1}}$/NO66 (LRA/4CCK); D) ligand RMSD KDE analysis.

Computational Methods

Crystal Data. X-ray crystal structures of ribosomal histidinyl hydroxylases (MINA53 and NO66) were downloaded from Protein Data Bank (PDB), i.e. two structures for MINA53, 2XDV (in complex with NOG) and 4BXF (in complex with 2OG), and one for NO66, 4CCK (in complex with NOG). 4BXF and 4CCK have 2 and 4 protein chains, respectively, each complexed with the proper ligand. These multi-chains PDB were loaded through UCSF Chimera v1.14, visually inspected, then split into a single chain and treated as single protein. Missing residues were built through homology modeling using Modeller v.10.1. Crystallographically observed solvent molecules were deleted, and missing hydrogens were added; residues side chains were ionized (protonated/deprotonated) at physiological pH (7.4). Geometric optimization was done by means the OpenMM tool using an in-house Python script. The minimized complexes were SB aligned on the alpha carbon atoms by means of UCSF Chimera MatchMaker module using the PDB: 2xdv structure as a reference.

Tautomeric structures. The three tautomeric forms of the **8** and **10** were built using Marvin Sketch software, then processed depending to the computational approach. For docking studies, the hydrogens at pH 7.4 were added and a random three-dimensional conformation was produced with Marvin sketch. A genetic algorithm-based conformational search using Obonformer was then used to optimize this 3-D conformation.

Molecular docking settings. ***Plants settings***. The docking of proteins with ligands was performed using Plants v1.2 version with three different scoring functions at the default speed (SPEED1). The docking tools generated 10 conformations for each docked ligand. The docking binding site was centered at the molecule's mean center and enlarged to a radius of 12 Å. Docking was performed using three different scoring functions: Chemplp, Plp and Plp95. ***Autodock Vina settings.*** Intermediary steps, such as pdbqt files for protein and ligand preparation, were completed using obabel software. The box was created by mean the Vina graphical interface module in UCSF Chimera. For each calculation, ten poses were obtained and ranked according to the scoring-functions.

QM Calculations. Starting from the three tautomeric forms of **8** and **10** represented as SMILES, a first three-dimensional geometry was obtained; the six structures were then subjected to a genetic algorithm-based conformational search returning the 30 lowest energy conformers from the last generation, optimized for RMSD diversity. This first manipulation was accomplished using OpenBabel (v. 2.4.0).[10] The resulting conformer structures were optimized at the semiempirical quantum mechanical level of theory PM6.[11] Each tautomer's lowest energy conformer was then subjected to further geometric optimization at a higher level of theory using the Minnesota DFT functional M06[12] equipped with the Pople split-valence triple-zeta basis set with polarization and diffuse functions on heavy atoms and hydrogens, 6-311++G**[13] Harmonic frequencies calculation were accomplished at the end of the geometry optimizations to evaluate zero-point vibrational energies, thermochemical corrections and to verify the nature of the stationary point. PCM solvation model was used for all QM calculations: PM6 and DFT ones. QM calculations were performed using the General Atomic and Molecular Electronic Structure System (GAMESS) software.[14]

MD Simulations. Protein structures were prepared as reported in crystal structure section: solvent molecules and crystallization residues were removed; gaps were filled by means of Modeller[15] and residue protonation states were defined using PROPKA3.[16] Ligand general amber force field 2 (GAFF2)[17] parameters were calculated by means of antechamber[18] at the semiempirical AM1-BCC level of theory.[19] The ff14SB force field[20] was used for proteins, while Li/Merz parameters were used for ions.[2121, 22] The complexes were solvated in an orthorhombic box using the four-point OPC water model[23] setting to 1.5 nm the distance between the box boundaries and the solute. Na^+ and Cl^- ions were added to neutralize the system. Complete parameters and topology files were obtained using tLeap. All parametrization procedures were accomplished using the AmberTools 20 suite.[24] The MD integration timestep was set to 2 fs, the hydrogen bond length was constrained using the LINCS algorithm,[25] long range electrostatics were treated using the particle mesh Ewald (PME)[26] method, temperature was imposed using a velocity-rescale thermostat.[27] MD simulations started with a 50000 step minimization applying harmonic position restraints to backbone atoms (k=1000 kJ/mol/nm^2) followed by a second unconstrained minimization. Systems were then heated gradually to the target temperature (300.15 K) during 1 ns constrained (k=100 kJ/mol/nm^2) simulation in an NVT ensemble. A second constrained equilibration (k=10 kJ/mol/ nm^2) occurred in an NPT ensemble where pressure control (1 atm) is achieved by means of a Berendsen barostat.[28] Finally, 25 ns production simulations in an NPT ensemble using a Parrinello-Rahman barostat[29] to control pressure (1 atm). MD simulations were performed with GROMACS (v. 2019.6).[30] Each simulation analysis was performed after the system had reached equilibration (i.e. stabilization of the backbone RMSD), so the first 5 ns of the trajectories were discarded. Binding free energy calculations were performed with the endpoint generalized Born and surface area continuum solvation (MM/GBSA) method[9] by means of the MMPBSA.py[31] Python script delivered with the AmberTools 18 suite. The entropy contribution was neglected, since the benefits coming from its calculation remain controversial and normal model calculations are computationally expensive.[32] The GB method used for MM/GBSA calculations was GB-Neck2.[33] This GB variant has the same form as the GBn method,[34] but uses a different parameter set. MD analysis were performed using the MDTraj[35] Python library. Statistical analysis such as KDE and PCA were performed with scipy[36] and the sckit-learn[37] Python library, respectively.

**Table S12.** Inhibitory activities of **10′** against MINA53 and NO66.			
**Compd.**	**Structure**	**MINA53 IC_{50} (μM)**	**NO66 IC_{50} (μM)**
**10′**	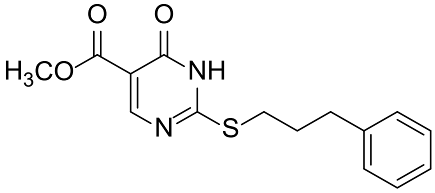	>100	20.1
**2,4-PDCA**	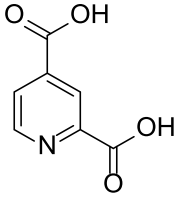	3.6	0.168

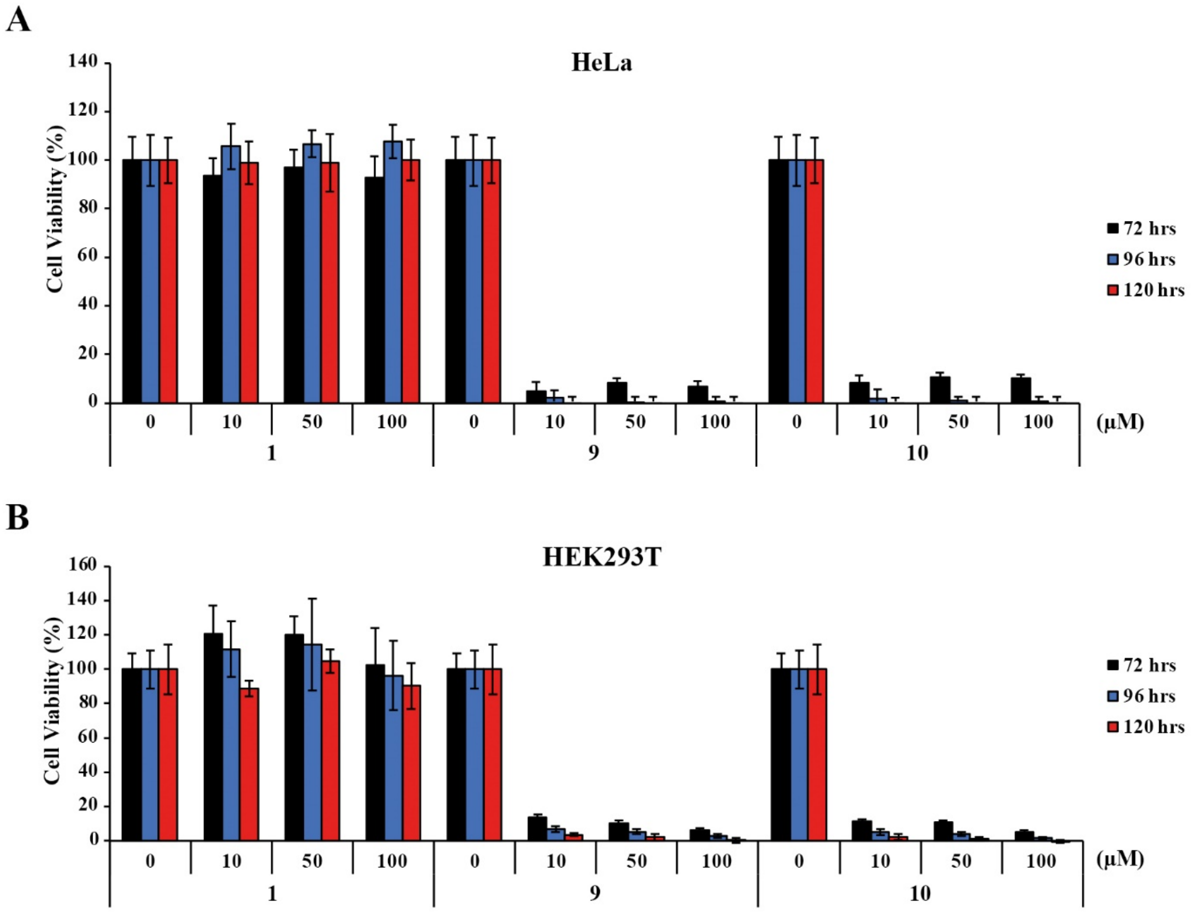

Figure S17. Antiproliferative effects of MINA53 inhibitors on HeLa (A) and HEK293T (B) cells as determined by the MTT method after exposure for 72, 96, and 120 h to the negative control **1**, and the MINA53 inhibitors **9** and **10**. The results are reported as (viability of drug-treated cells/viability of control cells) × 100 and represent the mean ± SD of two independent experiments performed in triplicate.

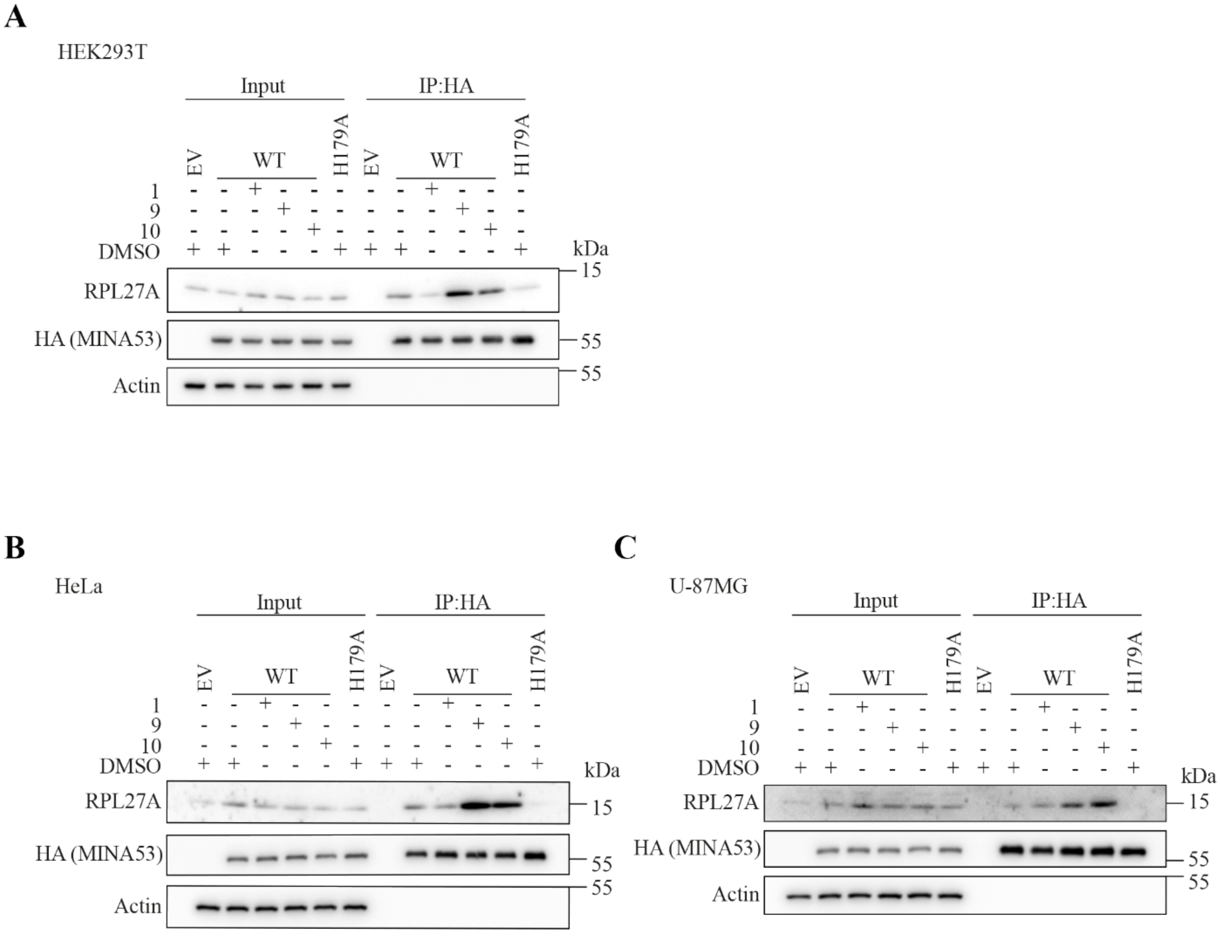

Figure S18. **MINA53 substrate trapping with inhibitors 9 and 10 in cells.** HEK293T (A), HeLa (B) and U-87MG (C) cells transiently expressing an empty (EV), wild-type (WT) or catalytically inactive (H179A) HA-MINA53 vector were incubated with 100 μM **1**, **9**, **10** or 0.5% (v/v) DMSO control for 18 h prior to anti-HA immunoprecipitation (IP). Anti-HA IPs were immunoblotted for RPL27A (17 kDa) and HA. β-Actin was used as loading control. Evidence for MINA53 substrate trapping is observed in all cell lines following incubation with **9** and **10**, but not **1.** Note that the H179A mutant served as a control likely lacking or having reduced substrate binding capability.

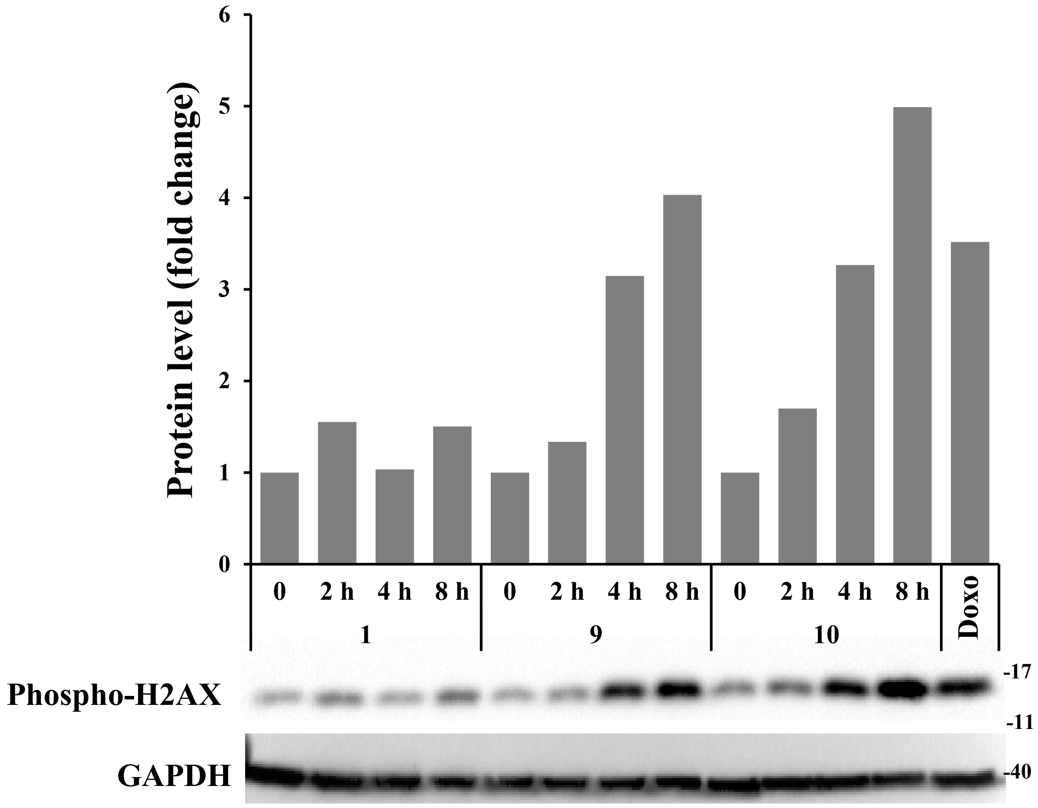

Figure S19. Western blot analysis and relative densitometric analysis of the phosphorylated form of H2AX (ser139) on U-87MG glioma cells exposed for the indicated times to **1** (negative control), **9** and **10** (MINA53 inhibitors) at 10 μM. Doxorubicin was used as a positive control (0.5 μM, 8 h). GAPDH expression was used as a control loading. Results are expressed as fold changes over untreated cells.

Purity control by HPLC of compounds 7-10

The purity of compounds **7-10** was analyzed by HPLC. The HPLC system consisted of a Waters 2695 (Waters, Milford, MA, USA) chromatograph equipped with an automatic injector, column heater and coupled with a model 996 PDA detector (Waters, Milford, MA, USA). The analytical controls were performed on Xterra RP_{18} 3.5 µm (3.9 x 100 mm) column (Waters, Milford, MA, USA) in gradient elution. Eluents: A) H_2O/CH_3CN, 95/5 + 0.05 % formic acid, B) CH_3CN/H_2O, 95/5 + 0.05 % formic acid. Gradient profile: start A/B 90/10, in 15 min 100% B, 20 min 100% B. Flow rate: 1.0 mL/min, T: room temperature.

Samples **7** and **8** were dissolved in MeOH/DMSO 9/1, samples **9** and **10** in MeOH at c: 1 mg/mL. Injection volume: 3 µL. By analysing the HPLC traces at 254 nm, a chemical purity > 97 % was recorded for compounds **7-10**. Specifically, for compound **7** the chemical purity was 97.76% (retention time 6.34 min), for compound **8** was 99.75% (retention time 5.36 min), for compound **9** was 98.67% (retention time 6.58 min), and finally for compound **10** the purity was 99.54% (retention time 7.21 min). In addition, chromatographic traces acquired at three wavelengths (214, 254, and 280 nm, respectively) for compounds **7**, **8**, **9**, and **10** were reported in Figures S20, S21, S22, and S23, respectively.

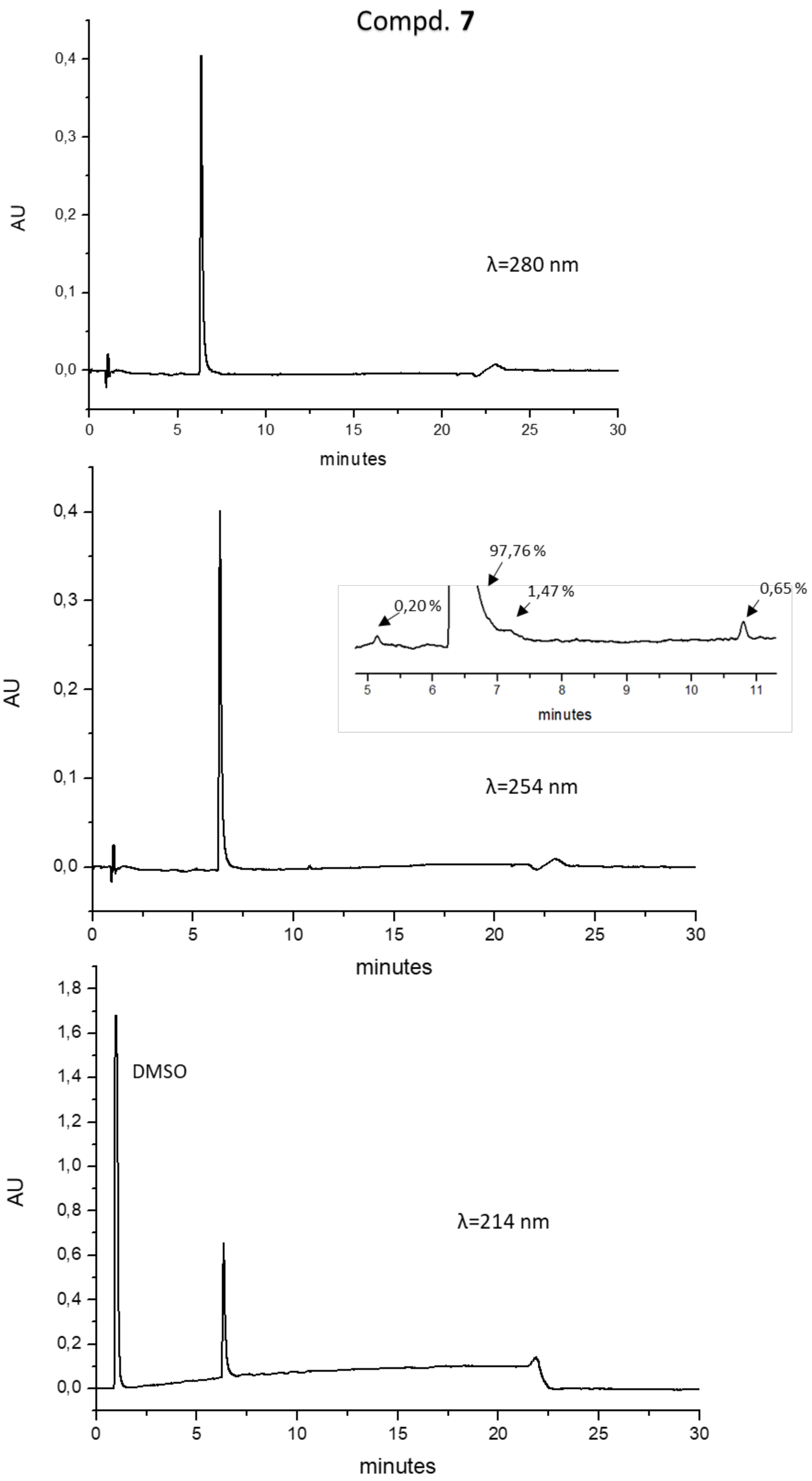

Figure S20. HPLC traces for compound **7**.

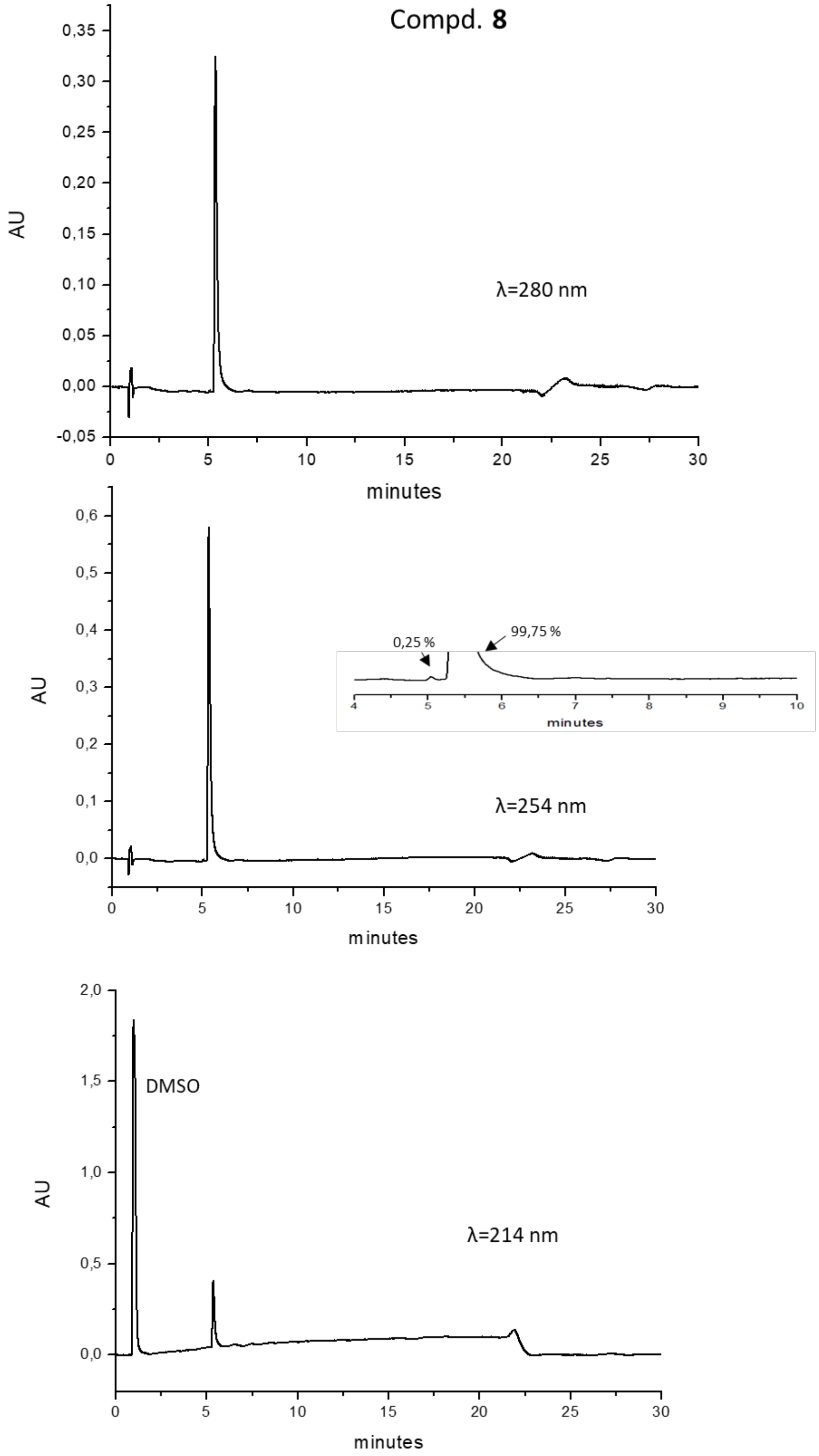

Figure S21. HPLC traces for compound **8**.

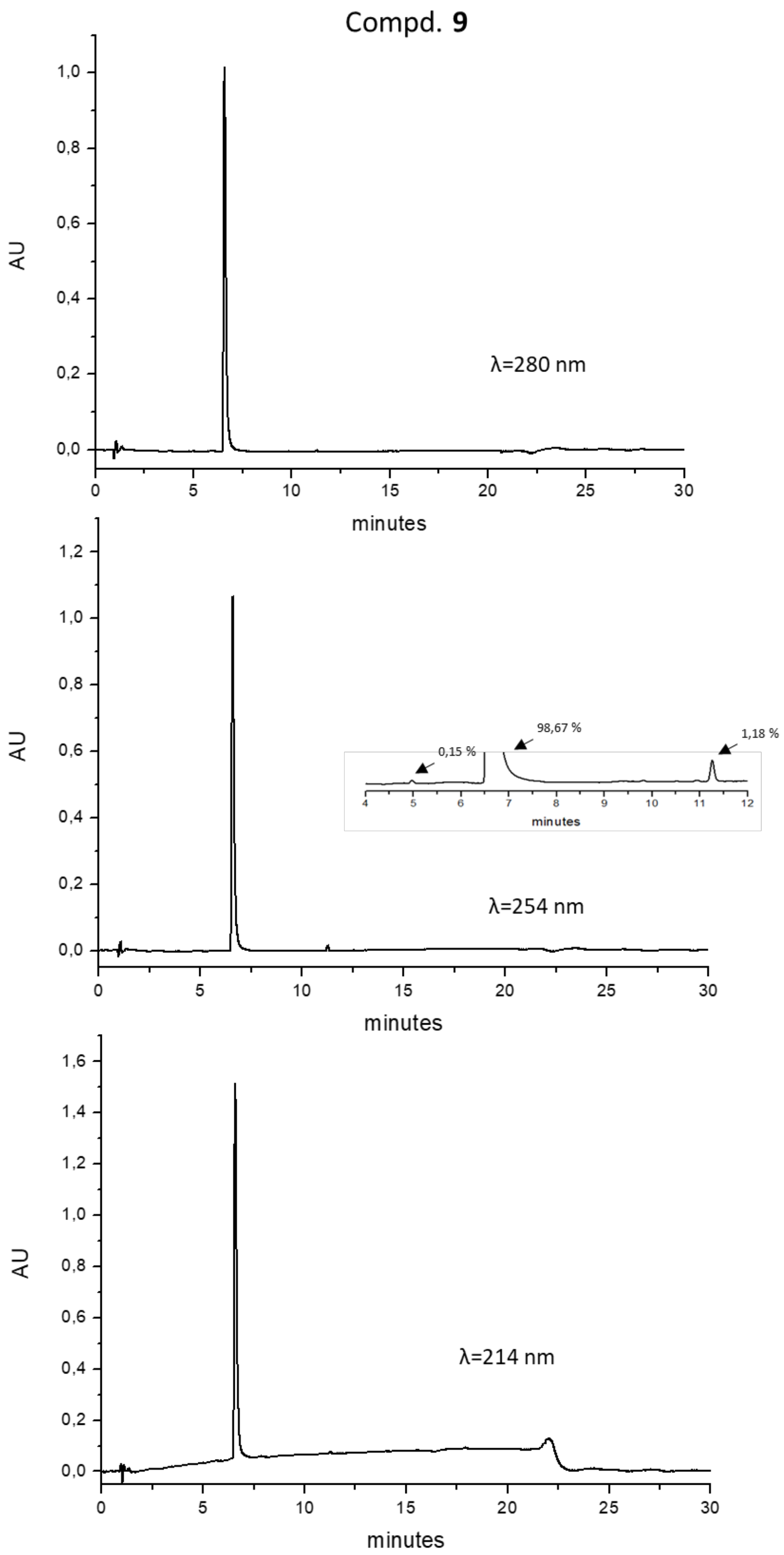

Figure S22. HPLC traces for compound **9**.

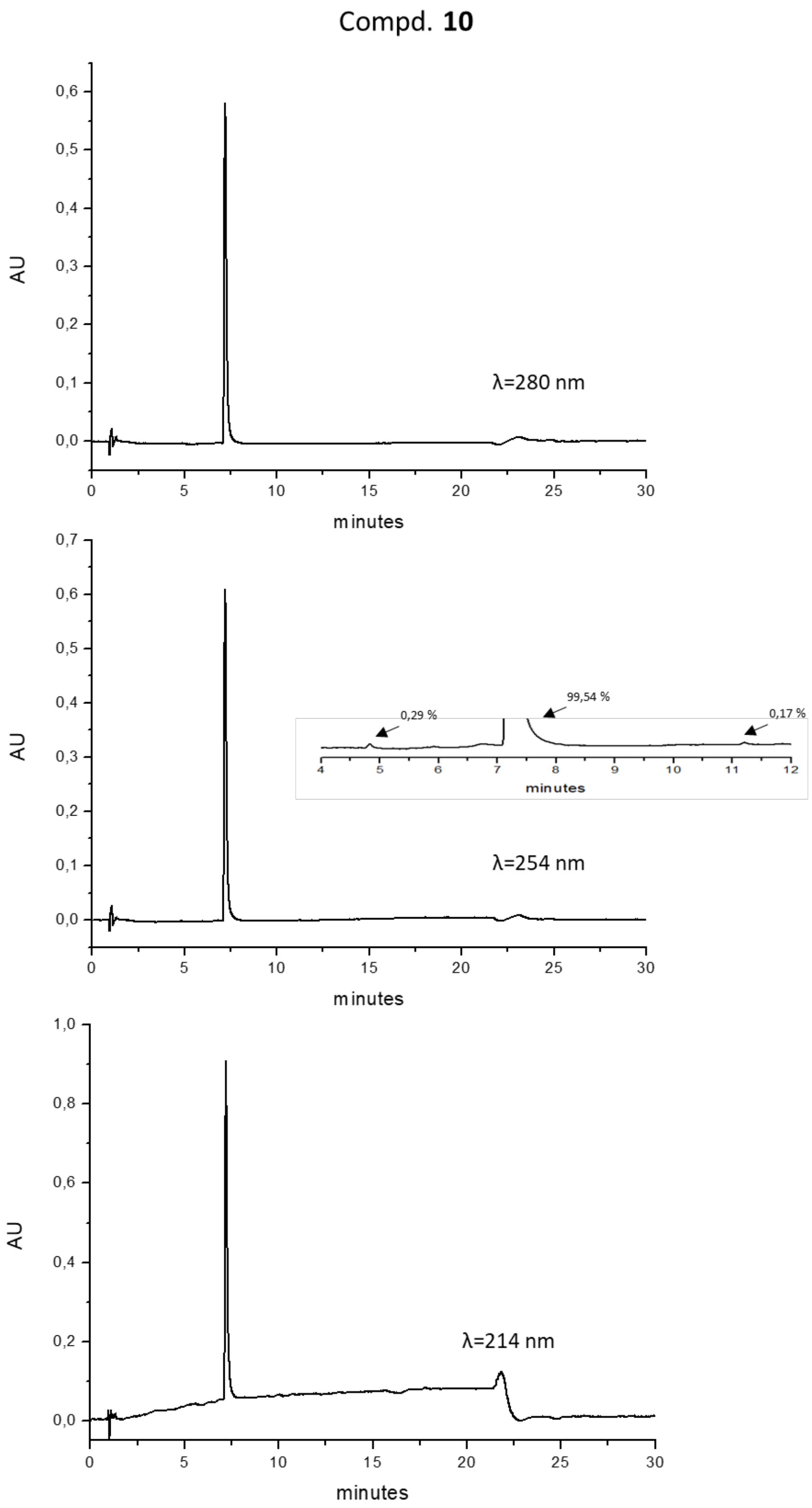

Figure S23. HPLC traces for compound **10**.

References

1. Carradori, S.; Rotili, D.; De Monte, C.; Lenoci, A.; D'Ascenzio, M.; Rodriguez, V.; Filetici, P.; Miceli, M.; Nebbioso, A.; Altucci, L.; Secci, D.; Mai, A., Evaluation of a large library of (thiazol-2-yl)hydrazones and analogues as histone acetyltransferase inhibitors: enzyme and cellular studies. *Eur J Med Chem* **2014,** *80*, 569-78.
2. Rose, N. R.; Woon, E. C.; Tumber, A.; Walport, L. J.; Chowdhury, R.; Li, X. S.; King, O. N.; Lejeune, C.; Ng, S. S.; Krojer, T.; Chan, M. C.; Rydzik, A. M.; Hopkinson, R. J.; Che, K. H.; Daniel, M.; Strain-Damerell, C.; Gileadi, C.; Kochan, G.; Leung, I. K.; Dunford, J.; Yeoh, K. K.; Ratcliffe, P. J.; Burgess-Brown, N.; von Delft, F.; Muller, S.; Marsden, B.; Brennan, P. E.; McDonough, M. A.; Oppermann, U.; Klose, R. J.; Schofield, C. J.; Kawamura, A., Plant growth regulator daminozide is a selective inhibitor of human KDM2/7 histone demethylases. *J Med Chem* **2012,** *55* (14), 6639-43.
3. Hopkinson, R. J.; Tumber, A.; Yapp, C.; Chowdhury, R.; Aik, W.; Che, K. H.; Li, X. S.; Kristensen, J. B. L.; King, O. N. F.; Chan, M. C.; Yeoh, K. K.; Choi, H.; Walport, L. J.; Thinnes, C. C.; Bush, J. T.; Lejeune, C.; Rydzik, A. M.; Rose, N. R.; Bagg, E. A.; McDonough, M. A.; Krojer, T.; Yue, W. W.; Ng, S. S.; Olsen, L.; Brennan, P. E.; Oppermann, U.; Muller-Knapp, S.; Klose, R. J.; Ratcliffe, P. J.; Schofield, C. J.; Kawamura, A., 5-Carboxy-8-hydroxyquinoline is a Broad Spectrum 2-Oxoglutarate Oxygenase Inhibitor which Causes Iron Translocation. *Chem Sci* **2013,** *4* (8), 3110-3117.
4. Sabatino, M.; Rotili, D.; Patsilinakos, A.; Forgione, M.; Tomaselli, D.; Alby, F.; Arimondo, P. B.; Mai, A.; Ragno, R., Disruptor of telomeric silencing 1-like (DOT1L): disclosing a new class of non-nucleoside inhibitors by means of ligand-based and structure-based approaches. *J Comput Aided Mol Des* **2018,** *32* (3), 435-458.
5. Trott, O.; Olson, A. J., AutoDock Vina: improving the speed and accuracy of docking with a new scoring function, efficient optimization, and multithreading. *J Comput Chem* **2010,** *31* (2), 455-61.
6. Korb, O.; Stutzle, T.; Exner, T. E., Empirical scoring functions for advanced protein-ligand docking with PLANTS. *J Chem Inf Model* **2009,** *49* (1), 84-96.
7. Ragno, R.; Ballante, F.; Pirolli, A.; Wickersham, R. B., 3rd; Patsilinakos, A.; Hesse, S.; Perspicace, E.; Kirsch, G., Vascular endothelial growth factor receptor-2 (VEGFR-2) inhibitors: development and validation of predictive 3-D QSAR models through extensive ligand- and structure-based approaches. *J Comput Aided Mol Des* **2015,** *29* (8), 757-76.
8. Caroli, A.; Ballante, F.; Wickersham, R. B., 3rd; Corelli, F.; Ragno, R., Hsp90 inhibitors, part 2: combining ligand-based and structure-based approaches for virtual screening application. *J Chem Inf Model* **2014,** *54* (3), 970-7.
9. Kollman, P. A.; Massova, I.; Reyes, C.; Kuhn, B.; Huo, S.; Chong, L.; Lee, M.; Lee, T.; Duan, Y.; Wang, W.; Donini, O.; Cieplak, P.; Srinivasan, J.; Case, D. A.; Cheatham, T. E., 3rd, Calculating structures and free energies of complex molecules: combining molecular mechanics and continuum models. *Acc Chem Res* **2000,** *33* (12), 889-97.
10. O'Boyle, N. M.; Banck, M.; James, C. A.; Morley, C.; Vandermeersch, T.; Hutchison, G. R., Open Babel: An open chemical toolbox. *J Cheminform* **2011,** *3*, 33.
11. Stewart, J. J., Optimization of parameters for semiempirical methods VI: more modifications to the NDDO approximations and re-optimization of parameters. *J Mol Model* **2013,** *19* (1), 1-32.
12. Zhao, Y.; Truhlar, D. G., The M06 suite of density functionals for main group thermochemistry, thermochemical kinetics, noncovalent interactions, excited states, and transition elements: two new functionals and systematic testing of four M06-class functionals and 12 other functionals. *Theoretical Chemistry Accounts* **2008,** *120* (1), 215-241.
13. Krishnan, R.; Binkley, J. S.; Seeger, R.; Pople, J. A., Self-consistent molecular orbital methods. XX. A basis set for correlated wave functions. *The Journal of Chemical Physics* **1980,** *72* (1), 650-654.

14. Barca, G. M. J.; Bertoni, C.; Carrington, L.; Datta, D.; De Silva, N.; Deustua, J. E.; Fedorov, D. G.; Gour, J. R.; Gunina, A. O.; Guidez, E.; Harville, T.; Irle, S.; Ivanic, J.; Kowalski, K.; Leang, S. S.; Li, H.; Li, W.; Lutz, J. J.; Magoulas, I.; Mato, J.; Mironov, V.; Nakata, H.; Pham, B. Q.; Piecuch, P.; Poole, D.; Pruitt, S. R.; Rendell, A. P.; Roskop, L. B.; Ruedenberg, K.; Sattasathuchana, T.; Schmidt, M. W.; Shen, J.; Slipchenko, L.; Sosonkina, M.; Sundriyal, V.; Tiwari, A.; Galvez Vallejo, J. L.; Westheimer, B.; Wloch, M.; Xu, P.; Zahariev, F.; Gordon, M. S., Recent developments in the general atomic and molecular electronic structure system. *J Chem Phys* **2020,** *152* (15), 154102.
15. Sali, A.; Blundell, T. L., Comparative protein modelling by satisfaction of spatial restraints. *J Mol Biol* **1993,** *234* (3), 779-815.
16. Olsson, M. H.; Sondergaard, C. R.; Rostkowski, M.; Jensen, J. H., PROPKA3: Consistent Treatment of Internal and Surface Residues in Empirical pKa Predictions. *J Chem Theory Comput* **2011,** *7* (2), 525-37.
17. Wang, J.; Wolf, R. M.; Caldwell, J. W.; Kollman, P. A.; Case, D. A., Development and testing of a general amber force field. *Journal of Computational Chemistry* **2004,** *25* (9), 1157-1174.
18. Wang, J.; Wang, W.; Kollman, P. A.; Case, D. A., Automatic atom type and bond type perception in molecular mechanical calculations. *J Mol Graph Model* **2006,** *25* (2), 247-60.
19. Jakalian, A.; Jack, D. B.; Bayly, C. I., Fast, efficient generation of high-quality atomic charges. AM1-BCC model: II. Parameterization and validation. *J Comput Chem* **2002,** *23* (16), 1623-41.
20. Maier, J. A.; Martinez, C.; Kasavajhala, K.; Wickstrom, L.; Hauser, K. E.; Simmerling, C., ff14SB: Improving the Accuracy of Protein Side Chain and Backbone Parameters from ff99SB. *J Chem Theory Comput* **2015,** *11* (8), 3696-713.
21. Li, P.; Roberts, B. P.; Chakravorty, D. K.; Merz, K. M., Jr., Rational Design of Particle Mesh Ewald Compatible Lennard-Jones Parameters for +2 Metal Cations in Explicit Solvent. *J Chem Theory Comput* **2013,** *9* (6), 2733-2748.
22. Li, P.; Merz, K. M., Taking into Account the Ion-Induced Dipole Interaction in the Nonbonded Model of Ions. *Journal of Chemical Theory and Computation* **2014,** *10* (1), 289-297.
23. Izadi, S.; Anandakrishnan, R.; Onufriev, A. V., Building Water Models: A Different Approach. *J Phys Chem Lett* **2014,** *5* (21), 3863-3871.
24. Case, D. A.; Ben-Shalom, I. Y.; Brozell, S. R.; Cerutti, D. S. a., Amber 2020. 2020.
25. Hess, B., P-LINCS: A Parallel Linear Constraint Solver for Molecular Simulation. *Journal of Chemical Theory and Computation* **2008,** *4* (1), 116-122.
26. Darden, T.; York, D.; Pedersen, L., Particle mesh Ewald: An N·log(N) method for Ewald sums in large systems. *The Journal of Chemical Physics* **1993,** *98* (12), 10089-10092.
27. Bussi, G.; Donadio, D.; Parrinello, M., Canonical sampling through velocity rescaling. *The Journal of Chemical Physics* **2007,** *126* (1), 014101.
28. Berendsen, H. J. C.; Postma, J. P. M.; van Gunsteren, W. F.; DiNola, A.; Haak, J. R., Molecular dynamics with coupling to an external bath. *The Journal of Chemical Physics* **1984,** *81* (8), 3684-3690.
29. Parrinello, M.; Rahman, A., Polymorphic transitions in single crystals: A new molecular dynamics method. *Journal of Applied Physics* **1981,** *52* (12), 7182-7190.
30. Abraham, M. J.; Murtola, T.; Schulz, R.; Páll, S.; Smith, J. C.; Hess, B.; Lindahl, E., GROMACS: High performance molecular simulations through multi-level parallelism from laptops to supercomputers. *SoftwareX* **2015,** *1-2*, 19-25.
31. Miller, B. R., 3rd; McGee, T. D., Jr.; Swails, J. M.; Homeyer, N.; Gohlke, H.; Roitberg, A. E., MMPBSA.py: An Efficient Program for End-State Free Energy Calculations. *J Chem Theory Comput* **2012,** *8* (9), 3314-21.
32. Mishra, S. K.; Koca, J., Assessing the Performance of MM/PBSA, MM/GBSA, and QM-MM/GBSA Approaches on Protein/Carbohydrate Complexes: Effect of Implicit Solvent Models, QM Methods, and Entropic Contributions. *J Phys Chem B* **2018,** *122* (34), 8113-8121.

33. Nguyen, H.; Roe, D. R.; Simmerling, C., Improved Generalized Born Solvent Model Parameters for Protein Simulations. *Journal of Chemical Theory and Computation* **2013,** *9* (4), 2020-2034.
34. Mongan, J.; Simmerling, C.; McCammon, J. A.; Case, D. A.; Onufriev, A., Generalized Born model with a simple, robust molecular volume correction. *J Chem Theory Comput* **2007,** *3* (1), 156-169.
35. McGibbon, Robert T.; Beauchamp, Kyle A.; Harrigan, Matthew P.; Klein, C.; Swails, Jason M.; Hernández, Carlos X.; Schwantes, Christian R.; Wang, L.-P.; Lane, Thomas J.; Pande, Vijay S., MDTraj: A Modern Open Library for the Analysis of Molecular Dynamics Trajectories. *Biophysical Journal* **2015,** *109* (8), 1528-1532.
36. Virtanen, P.; Gommers, R.; Oliphant, T. E.; Haberland, M.; Reddy, T.; Cournapeau, D.; Burovski, E.; Peterson, P.; Weckesser, W.; Bright, J.; van der Walt, S. J.; Brett, M.; Wilson, J.; Millman, K. J.; Mayorov, N.; Nelson, A. R. J.; Jones, E.; Kern, R.; Larson, E.; Carey, C. J.; Polat, I.; Feng, Y.; Moore, E. W.; VanderPlas, J.; Laxalde, D.; Perktold, J.; Cimrman, R.; Henriksen, I.; Quintero, E. A.; Harris, C. R.; Archibald, A. M.; Ribeiro, A. H.; Pedregosa, F.; van Mulbregt, P.; SciPy, C., SciPy 1.0: fundamental algorithms for scientific computing in Python. *Nat Methods* **2020,** *17* (3), 261-272.
37. Pedregosa, F.; Varoquaux, G.; Gramfort, A.; Michel, V.; Thirion, B.; Grisel, O.; Blondel, M.; Prettenhofer, P.; Weiss, R.; Dubourg, V.; Vanderplas, J.; Passos, A.; Cournapeau, D.; Brucher, M.; Perrot, M.; Duchesnay, É., Scikit-learn: Machine Learning in Python. *J. Mach. Learn. Res.* **2011,** *12* (null), 2825–2830.
